# Targeted Degradation of XIAP is Sufficient and Specific to Induce Apoptosis in MYCN-overexpressing High-risk Neuroblastoma

**DOI:** 10.1158/2767-9764.CRC-23-0082

**Published:** 2023-11-22

**Authors:** Zhang'E Choo, Xiaoying Koh, Megan Rui En Wong, Ruth Minothini Ashokan, Nurul Suhana Binte Ali Ahamed, CongBao Kang, Chik Hong Kuick, Kenneth Tou En Chang, Sarit Larisch, Amos Hong Pheng Loh, Zhi Xiong Chen

**Affiliations:** 1 Department of Physiology, Yong Loo Lin School of Medicine, National University of Singapore, Singapore.; 2 NUS Centre for Cancer Research, Yong Loo Lin School of Medicine, National University of Singapore, Singapore.; 3 Experimental Drug Development Centre, A*STAR, Singapore.; 4 VIVA-KKH Paediatric Brain and Solid Tumour Programme, Children's Blood and Cancer Centre, KK Women's and Children's Hospital, Singapore.; 5 Department of Pathology and Laboratory Medicine, KK Women's and Children's Hospital, Singapore.; 6 Duke NUS Medical School, Singapore.; 7 Cell Death and Cancer Research Laboratory, Department of Human Biology and Medical Sciences, University of Haifa, Haifa, Israel.; 8 Department of Paediatric Surgery, KK Women's and Children's Hospital, Singapore.; 9 National University Cancer Institute, Singapore, National University Health System, Singapore.

## Abstract

XIAP, the most potent mammalian inhibitor of apoptosis protein (IAP), critically restricts developmental culling of sympathetic neuronal progenitors, and is correspondingly overexpressed in most *MYCN*-amplified neuroblastoma tumors. Because apoptosis-related protein in the TGFβ signaling pathway (ARTS) is the only XIAP antagonist that directly binds and degrades XIAP, we evaluated the preclinical effectiveness and tolerability of XIAP antagonism as a novel targeting strategy for neuroblastoma. We found that antagonism of XIAP, but not other IAPs, triggered apoptotic death in neuroblastoma cells. XIAP silencing induced apoptosis while overexpression conferred protection from drug-induced apoptosis. From a screen of IAP inhibitors, first-in-class ARTS mimetic A4 was most effective against high-risk and high XIAP-expressing neuroblastoma cells, and least toxic toward normal liver- and bone marrow–derived cells, compared with pan-IAP antagonists. On target engagement assays and nuclear magnetic resonance spectroscopy, A4 was observed to degrade rather than inhibit XIAP, catalyzing rapid degradation of XIAP through the ubiquitin-proteasome pathway. In *MYCN*-amplified neuroblastoma patient-derived xenografts, A4 significantly prolonged survival as a single agent, and demonstrated synergism with standard-of-care agents to reduce their effective required doses 3- to 6-fold. Engagement and degradation of XIAP by ARTS mimetics is a novel targeting strategy for neuroblastoma that may be especially effective against *MYCN*-amplified disease with intrinsically high XIAP expression. First-in-class ARTS mimetic A4 demonstrates preclinical efficacy and warrants further development and study.

**Significance:**

XIAP degradation is sufficient to kill *MYCN*-amplified neuroblastoma which overexpresses and relies on XIAP as a brake against cell death, without affecting normal cells.

## Introduction

Neuroblastoma, a malignant embryonal tumor of the sympathetic nervous system, is the leading cause of death in children less than 5 years old and accounts for a disproportionate 15% of all childhood cancer mortalities (1, 2). High-risk disease is associated with limited response to existing treatment options and poor survival outcomes despite intensive multimodal therapy (3). Therefore, new treatment strategies are urgently needed for high-risk neuroblastoma.

Developmental apoptosis of neuronal precursors is crucial in determining the final number of terminally differentiated cells. Aberrant developmental apoptosis is implicated in the development of embryonal nervous system tumors including neuroblastoma (4, 5). The putative cellular origin of neuroblastoma is thought to be primitive sympathetic neural precursors of sympathoadrenal lineage which have failed to undergo apoptosis in response to developmentally-timed trophic factor withdrawal signals, and thus persist as tumor-initiating cells (6, 7).

XIAP, the best-characterized member of the inhibitor of apoptosis (IAP) family, is an important regulator of neuronal culling during neural crest development and neuroectodermal differentiation (8, 9). Reduced levels of XIAP, rather than other IAPs, is necessary for developmental apoptosis to proceed, and its upregulation has been found to be associated with chemotherapy resistance and unfavorable outcome in relapsed and advanced stage neuroblastoma (10, 11). Greater overexpression of XIAP over other IAPs has also been noted in other neuroectodermal cancers such as melanoma and neuroendocrine tumors (12, 13). Furthermore, we previously showed that loss of XIAP-intrinsic antagonist, XAF1, results in failure of caspase-mediated cell death in neuroblastoma, and is associated with poorer survival and disease outcomes (14). Therefore, we surmise that neuroblastoma, particularly high-risk tumors, are addicted to and depend on XIAP for survival, and that targeting XIAP rather than other IAPs can yield specific and effective apoptotic cell death.

Several XIAP-binding proteins have been identified and these include second mitochondria-derived activator of caspase/direct inhibitor of apoptosis protein-binding protein with low propidium iodide (PI; SMAC/DIABLO), high temperature requirement protein A2 (Omi/HtrA2), XAF1, and apoptosis-related protein in the TGFβ signaling pathway (ARTS) (15–18). Based on the known binding structure of these proteins to XIAP, several small-molecule mimetics have been developed for use as potential treatment agents. For instance, SMAC mimetics, which are pan-IAP antagonists, have significant therapeutic value either alone or in combination with other chemotherapeutics for treating a variety of cancers (19). These compounds inhibit both IAPs, though with higher affinity and potency for c-IAPs than XIAP (20). In contrast, ARTS mimetics are a newly-described class of molecules that specifically target XIAP (21), but there have been limited studies on the effectiveness of targeting XIAP in neuroblastoma. In this study, we evaluated the effectiveness and therapeutic value of different IAP antagonists on neuroblastoma and present a new XIAP-specific targeting strategy using ARTS mimetics. We also demonstrated that XIAP degradation, rather than inhibition, is a promising novel treatment approach for high-risk neuroblastoma.

## Materials and Methods

### Cell Lines and Cell Culture

Human neuroblastoma cell lines (SK-N-SH, SK-N-AS, NB1, CHP212, NLF) were cultured in RPMI1640, KELLY in RPMI1640 with 25 mmol/L HEPES, BE(2)-C in DMEM/F12 (1:1) with 15 mmol/L HEPES and IMR-32 in MEM/EBSS with 1% non-essential amino acids and 1% sodium pyruvate. Human noncancerous normal cell lines, HS5 (bone marrow origin) and THLE3 (liver origin) were cultured in DMEM/High Glucose with 1% sodium pyruvate and 1% sodium bicarbonate, and Bronchial Epithelial Cell Growth Medium, respectively. All media were supplemented with 10% FBS. Patient-derived neuroblastoma cells (NBL27-0218A, NBL16-0118, NBL01-1116, NBL07-0317, NBL01-1116) were generated and cultured as described previously (22). SK-N-SH, SK-N-AS, CHP212, BE(2)-C, and THLE3 cell lines were obtained from the ATCC. NB1 cells were obtained from the Japanese Collection of Research Bioresources, NLF cells were obtained from Kerafast, and KELLY cells were obtained from Sigma-Aldrich. All cell lines were cultured less than 20 passages from thawed vials and maintained at 37°C in 5% CO_2_ humidified incubator, and mostly subcultured twice a week. Short tandem repeat genotyping of commercial cell lines and tumor-cell line pairs was performed using PowerPlex 21 (Promega, catalog no. DC8902) using 1 ng of DNA. PCR products were resolved in an Applied Biosystems SeqStudio genetic analyzer and compared with Cellosaurus and ATCC references where available. *Mycoplasma* testing was performed using MycoSEQ Myco Scan detection kit (Life Technologies, catalog no. 4482224).

### Animal Models

Neuroblastoma patient-derived xenografts (PDX) were generated from *MYCN*-amplified tumor samples of a patient with stage III high-risk undifferentiated neuroblastoma recruited with waiver of written parental consent and child assent, under SingHealth Duke NUS CIRB protocol 2014/2079 (Modeling, Analysis, and Translational Therapeutics for Tumors of Childhood), and implanted orthotopically in the retroperitoneal space of NOD/SCID mice. All experiments were performed under the approval of the Institutional Animal Care and Use Committee (SingHealth Duke NUS IACUC #1066). Histologic morphology of PDX tumors were compared with the original patient tumors at each passage to establish their congruence, and also confirmed with supporting IHC stains with neuroblastoma markers PHOX2B, TH and GD2 Synthase.

### Test Compounds Preparation

All IAP antagonists were provided in powder and reconstituted in DMSO (MP Biomedicals). A4 and B3 were obtained from Carmel – Haifa University Economic Corporation Ltd., CUDC-427 was obtained from Curis, Inc., LCL161 was obtained from Novartis Pte Ltd., Debio1143 was obtained from Debiopharm International SA, and BV6 was obtained from Genentech, Inc., A4, B3, and BV6 were stored in −80°C after reconstitution into stock concentration of 40 mmol/L (A4 and B3) and 20 mmol/L (BV6), respectively. LCL161, CUDC-427, and Debio1143 were stored in −20°C after reconstitution into stock concentration of 40 mmol/L.

### Cell Viability Assays

Cell viability was measured in real time using RealTime-Glo MT Cell Viability Assay as according to manufacturer's instructions from Promega. Cells were seeded at 1 × 10^4^ cells per well and treated with a range of doses of indicated antagonists, followed by continuous bioluminescence reading every 24 hours for 3 days using luminescent plate reader, Varioskan (SkanIT software). IC_50_ was determined using GraphPad Prism software.

### Apoptotic Assays

The determination of caspase-3/7 levels as an indication for apoptosis activity was performed using a Caspase-Glo 3/7 assay kit from Promega according to manufacturer's instructions. Cells were seeded at 1 × 10^4^ cells per well and treated with 10 µmol/L of IAP antagonists for various indicated timings, followed by bioluminescence measurement using luminescent plate reader, Tecan Infinite200 Pro. The activity of apoptosis was also measured by flow cytometry using the Dead Cell Apoptosis Kit with Annexin V FITC and PI (Thermo Fisher Scientific) according to manufacturer's instructions. Cells harvested from lentiviral infection were subjected to Annexin V (2.5 µL) and PI (40 ng/mL, 0.5 µL) staining, followed by flow cytometric analysis using BD LSRFortessa cell analyzer with excitation/emission spectra of 494 nm/518 nm for FITC and 535 nm/617 nm for PI.

### Nuclear Magnetic Resonance Spectroscopy on XIAP

Uniformly ^13^C,^15^N-labeled XIAP of 0.8–1.0 mmol/L concentration was used for nuclear magnetic resonance (NMR) studies. NMR experiments were carried out at 298 K (25°C) on a Bruker Avance spectrometer with a proton frequency of 600 or 700 MHz and equipped with a cryoprobe. Sequence-specific assignment was obtained on the basis of the following experiments including ^1^H-^15^N-HSQC, HNCA, HNCACB, HN(CO)CA, HN(CO)CACB, and HNCO. Spectra were acquired using standard pulses from Topspin (version 2.1). To determine XIAP interaction with small molecule A4, ^1^H-^15^N-HSQC experiment was performed using 50 mmol/L of A4 compound dissolved in DMSO and 0.5 mmol/L of ^15^N-labeled XIAP. The ^1^H-^15^N-HSQC spectra of XIAP in the absence and presence of different amounts of A4 compounds were acquired, processed, and analyzed. The collected data were processed with NMRPipe (23) and analyzed with NMRView (24).

### Drug Interactions Assay

Drug interactions between XIAP-specific antagonist A4 and vincristine or topotecan were determined using the combination index (CI) by Chou and Talalay which was established on the basis of the mass-action law principle to derive the median-effect equation (25). A total of 1 × 10^4^ cells were seeded in 96-well plate and treated with increasing doses of A4 and vincristine/topotecan (individually or in combination) at a fixed ratio according to the IC_50_ values of the individual drugs for 48 hours [A4:vincristine; BE(2)-C 1:1 (0–100 µmol/L A4: 0–100 nmol/L Vin), KELLY 1:0.5 (0–100 µmol/L A4: 0–50 nmol/L Vin) and A4:topotecan; BE(2)-C 1:6 (0–100 µmol/L A4: 0–600 nmol/L Topo), KELLY 1:16 (0–100 µmol/L A4: 0–1600 nmol/L)]. Cell viability was measured and CI and dose reduction index (DRI) values were quantified using the CompuSyn software by Chou–Talalay. Areas under the dose–response curves were calculated using the trapezoidal method and compared using paired *t* test.

### *In Vivo* Experiments

Tumor samples collected from neuroblastoma PDXs were infected with luciferase lentivirus. Tumor analysis was performed by intraperitoneal injection of d-luciferin solution (150 mg/kg; PerkinElmer) into the mice and imaged with an IVIS spectrum. Bioluminescent signals were then quantified using Living Imaging 4.4 (Caliper Life Sciences). After 4 weeks of tumor implantation into retroperitoneal space of mice, tumor-bearing mice were randomly assigned to receive either vehicle (DMSO; 1 mL/kg) or XIAP-specific antagonist A4 (10 mg/kg; *n* = 9 per group). All agents were administered by intraperitoneal injection twice weekly for consecutive 3 weeks. General health state of the mice were monitored weekly and were euthanized upon reaching humane endpoints. Survival data were recorded and Kaplan–Meier curves were plotted using GraphPad Prism software.

### Data Availability

All data generated or analyzed during this study are included in the article and its Supplementary Data files, and are available upon request. All models used in the article are available upon request.

## Results

### XIAP is Overexpressed in High-risk Neuroblastoma and Induces Apoptosis When Knocked Down

**FIGURE 1:**
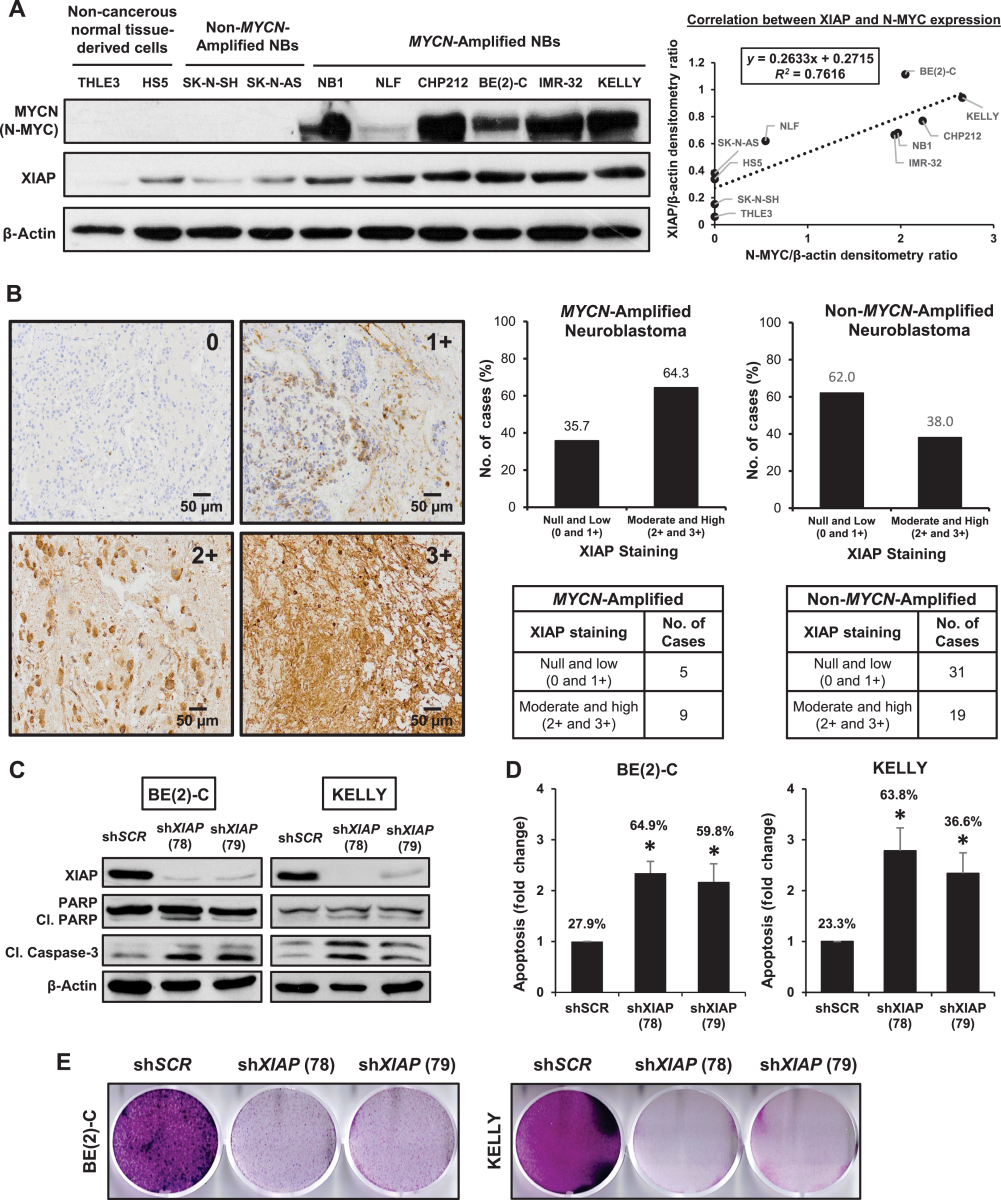
XIAP is overexpressed in high-risk neuroblastoma and induces apoptosis when knocked down. **A,** Left, Immunoblot analysis of N-Myc and XIAP expressions in eight human neuroblastoma cell lines and noncancerous normal tissue-derived cell lines (THLE3: liver-derived cells; HS5: bone marrow–derived cells). β-actin is used as internal control. Right, Scatterplot of corresponding densitometry ratio for N-Myc and XIAP expression relative to β-actin with linear trend fit (*R*^2^ correlation = 0.76). **B,** Left, Representative images of XIAP IHC staining in a tissue microarray (*n* = 64; 20X), indicating staining intensity of XIAP with 0 = null, 1+ = low, 2+ = moderate, 3+ = high. Right, Graphical distribution of XIAP staining in *MYCN*-amplified neuroblastoma cases (*n* = 14) and non–*MYCN*-amplified neuroblastoma cases (*n* = 50; Supplementary Data S1). **C,** Immunoblot analysis of BE(2)-C and KELLY cell lines transduced with lentivirus encoding shRNAs targeting XIAP (sh*XIAP* 78 and 79) or nontargeting control virus (sh*SCR*). β-actin is used as internal control. Control virus sh*SCR* served as negative control. **D,** Graphical representation showing fold change of apoptotic-positive BE(2)-C and KELLY cells transduced with shRNAs targeting XIAP (sh*XIAP* 78 and 79) relative to cells transduced with nontargeting control (sh*SCR*; data were expressed as mean ± SD of three independent experiments; *, *P* < 0.05). Apoptotic population was evaluated by flow cytometry analysis of cells subjected to AV/PI staining (mean percentages of Annexin V-positive cells are indicated). Control virus sh*SCR* served as negative control. **E,** Crystal violet staining to determine cell viability and colony formation after lentiviral-induced silencing of XIAP and selected with 1 µg/mL (BE(2)-C) or 500 ng/mL puromycin (KELLY) for several weeks. Control virus sh*SCR* served as negative control. Purple stained cells represent viable cells.

During development, reduction in N-Myc and XIAP expression is necessary for nerve growth factor withdrawal-mediated apoptosis in sympathoadrenal progenitor cells, failure of which prolongs survival of these cells and promote tumorigenesis (10, 26). We hypothesized that *MYCN*-amplified neuroblastomas would have concomitant high XIAP expression. To investigate, we screened N-Myc and XIAP expression across a panel of neuroblastoma, and normal liver (THLE3)-and bone marrow (HS5)-derived cell lines. Liver and bone marrow are common metastatic sites for neuroblastoma (Fig. 1A).

In general, the majority of neuroblastoma cell lines expressed higher XIAP protein levels compared with noncancerous normal tissue-derived cell lines. Specifically, the expression of XIAP was higher in *MYCN*-amplified neuroblastoma cells compared with non–*MYCN*-amplified cells (positive correlation with *R*^2^ = 0.76; Fig. 1A; Supplementary Table S1). This was supported by examining a tissue microarray of neuroblastoma patient samples where 64.3% of patients with *MYCN* amplification expressed moderate (2+) to high (3+) XIAP whereas the majority of non–*MYCN*-amplified patients had null (0) to low (1+) XIAP expression (Fig. 1B; Supplementary Data S1). This suggested that XIAP is highly expressed in most neuroblastoma tumors and is positively correlated to *MYCN* amplification status.

Because XIAP reduction is necessary to make sympathetic neuronal progenitors vulnerable to developmental apoptosis (10), we investigated how removal of high endogenous XIAP expression would impact apoptosis in neuroblastoma cells. Using two independent lentiviral short hairpin RNAs (shRNA) targeting XIAP transcripts (sh*XIAP* 78 and 79), endogenous XIAP expression was knocked down (Fig. 1C). In contrast to nontargeting shRNA (sh*SCR*) controls, silencing of XIAP in high XIAP-expressing neuroblastoma cell lines BE(2)-C and KELLY significantly induced apoptosis, indicated by increased cleavage of PARP and caspase-3 via Western blot analysis, and significant increase in apoptotic cells quantified via flow cytometric analysis (Fig. 1C and D). Furthermore, silencing of XIAP decreased colony formation over time (Fig. 1E). Consistent with previous findings, these results demonstrated that XIAP expression is necessary for survival of neuroblastoma cells.

### Specific Loss of XIAP Expression, not c-IAP1, is Required for Mediating Apoptosis in High-risk Neuroblastoma Cells

To investigate the efficacy of targeting XIAP as a treatment strategy for neuroblastoma, six small-molecule antagonists, representing a variety of chemical structures and IAP-targeting specificity, were evaluated (Supplementary Table S2; refs. 21, 27–30).

**FIGURE 2:**
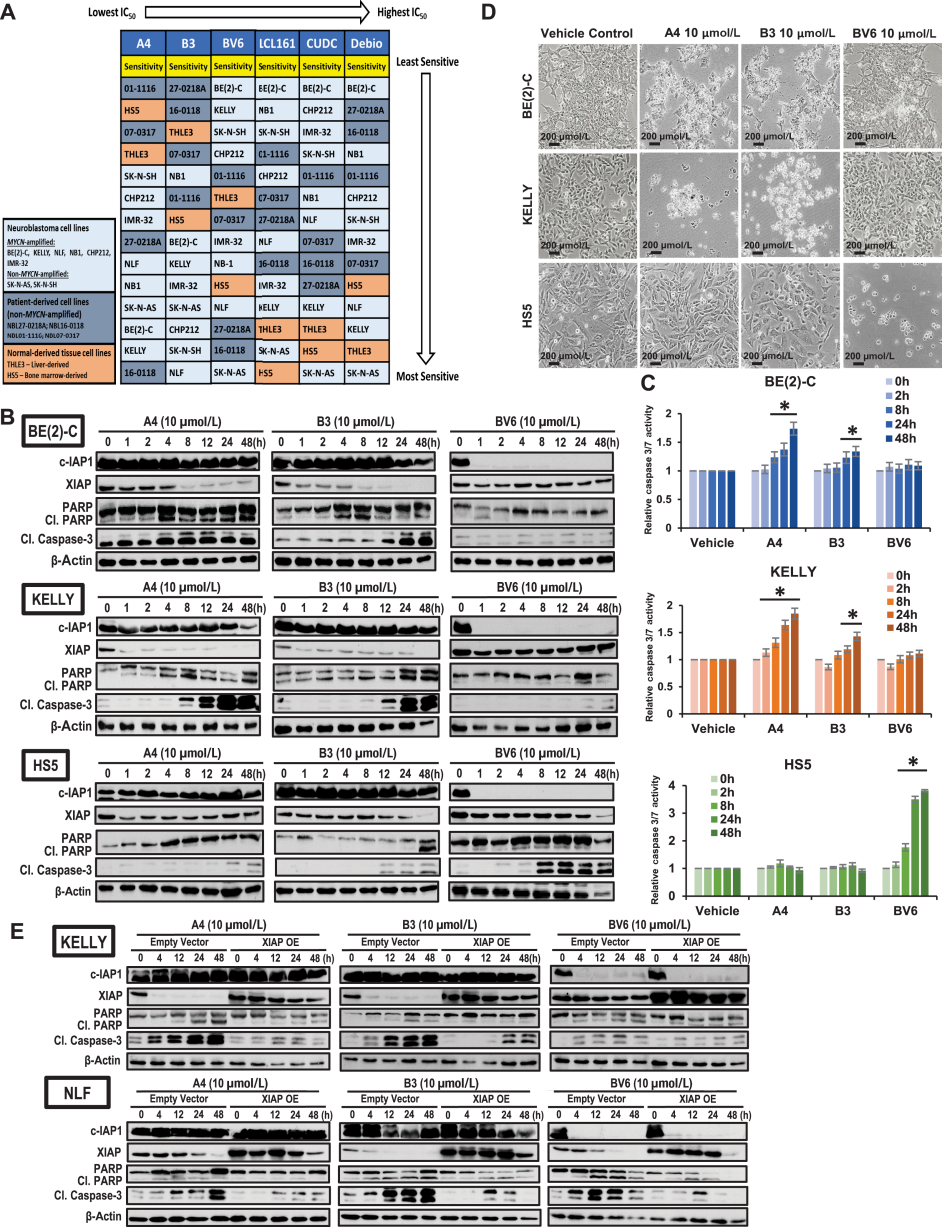
Specific loss of XIAP expression, not c-IAP1, is required for mediating apoptosis in high-risk neuroblastoma cells. **A,** Summary of IAP antagonists’ efficacy in killing neuroblastoma cells. The IAP antagonists were ranked on the basis of the average potency of killing (IC_50_) and specificity of killing toward only neuroblastoma cells: (i) A4, (ii) B3, (iii) BV6, (iv) LCL161, (v) CUDC-427, (vi) Debio 1143. **B,** Immunoblot analysis of c-IAP1, XIAP, PARP, and cleaved caspase-3 expression in neuroblastoma cell lines, BE(2)-C and KELLY, and noncancerous normal tissue cell line, HS5, after treatment with 10 µmol/L of either A4, B3, or BV6 at indicated timings (three independent replicates). **C,** Graphical representation showing the fold change of caspase-3/7 activity relative to vehicle controls after treatment with 10 µmol/L of either A4, B3, or BV6 at indicated timings (data were expressed as mean ± SD of three independent experiments conducted in duplicates; *, *P* < 0.05). **D,** Morphology of cells 48 hours posttreatment with 10 µmol/L of either A4, B3, or BV6. Images were taken using a light microscope with 10X magnification enlarged (scale: 1 cm: 200 µm). **E,** Immunoblot analysis of c-IAP1, XIAP, PARP, and cleaved caspase-3 expression in neuroblastoma cell lines, KELLY and NLF transfected with plasmid encoding either XIAP or empty vector, followed by treatment with 10 µmol/L of A4, B3, or BV6 at indicated timings (three independent replicates). β-actin is used as internal control.

To determine their efficacy in neuroblastoma, a panel of neuroblastoma cell lines including neuroblastoma patient-derived cell lines and noncancerous normal tissue-derived cell lines were treated with increasing doses of each antagonist (0–100 µmol/L), followed by real-time measurement of cell viability every 24 for 72 hours. Among the six IAP antagonists, XIAP-specific antagonists A4 and B3 showed highest potency against neuroblastoma cells across the panel of cell lines with the lowest IC_50_ achieved (2–15 µmol/L; Supplementary Table S2; Fig. 2A). Intriguingly, the remaining four pan-IAP antagonists showed poorer average efficacy, ranked by mean IC_50_: BV6 with IC_50_ of 3 to 20 µmol/L, LCL161 and CUDC-427 with IC_50_ 20 to 60 µmol/L, and Debio 1143 with IC_50_ 50 to >100 µmol/L (Supplementary Table S2; Fig. 2A). Among the four pan-IAP antagonists with the same inhibitory mechanism, BV6 was the most potent in suppressing neuroblastoma cells. Though the overall IC_50_ range of A4, B3, and BV6 on neuroblastoma cells were comparable despite the different modes of action, their relative effects on noncancerous cells have not been previously studied.

To evaluate this, noncancerous normal tissue-derived cell lines THLE3 and HS5 were utilized to evaluate toxicity and tolerability. XIAP-specific antagonists A4 and B3 were the least toxic to noncancerous cells and were the most discriminatory in killing neuroblastoma cells (Supplementary Table S2; Fig. 2A). In contrast, the pan-IAP antagonists especially LCL161, CUDC-427, and Debio 1143, were more toxic toward noncancerous cells than neuroblastoma cells and thus, deemed unsuitable for use in neuroblastoma treatment. On the basis of their overall potency and specificity toward neuroblastoma cells over noncancerous normal cells, A4, B3, and BV6 were selected as the more efficacious antagonists to be studied further in subsequent experiments (Fig. 2A).

Interestingly, high-risk and *MYCN*-amplified neuroblastoma cell lines BE(2)-C and KELLY expressing high XIAP levels, were generally sensitive to XIAP-specific antagonists A4 and B3 but were highly resistant to pan-IAP antagonist BV6 (Figs. 1A and 2A). This suggests that specific targeting of XIAP could be a potential treatment strategy for high-risk neuroblastoma. To further investigate this, we studied the impact of A4, B3, and BV6 on the expression of XIAP and c-IAP1 and subsequent downstream effects, using BE(2)-C and KELLY as representative cell lines of high-risk neuroblastoma and HS5 as control for toxicity and tolerability.

BE(2)-C and KELLY cells treated with A4 or B3 showed loss of XIAP expression over time with intact c-IAP1 expression (Fig. 2B). Correspondingly, apoptosis was induced, indicated by increasing cleavage of PARP and caspase-3 as well as increased caspase-3/7 activity (Fig. 2B and C). In contrast, BE(2)-C and KELLY cells treated with BV6 showed loss of c-IAP1 expression with no change in XIAP expression (Fig. 2B). Unsurprisingly, BV6 induced minimal or no apoptosis in BE(2)-C and KELLY cells despite the loss of c-IAP1 expression (Fig. 2B and C). This suggested that targeting of XIAP, and not c-IAP1, is required for the killing of high-risk neuroblastoma. Consistent with our cell viability findings, evaluation of cell morphologic changes confirmed that BE(2)-C and KELLY cells were sensitive to cell killing by A4 and B3 but resistant to BV6 treatment (Fig. 2D).

In contrast to neuroblastoma cells, normal tissue-derived HS5 cells treated with A4 or B3 showed minimal change in XIAP expression with c-IAP1 remaining intact (Fig. 2B) and minimal apoptosis observed (Fig. 2B), possibly due to lower expression and lesser dependency on endogenous XIAP, and thus, higher tolerability toward A4 and B3. Indeed, BE(2)-C, KELLY, and HS5 cells treated with BV6 showed loss of c-IAP1 expression with no changes in XIAP expression (Fig. 2B). However, unlike BE(2)-C and KELLY, BV6 induced significant apoptosis in HS5 cells (Fig. 2B and C). Likewise, cell morphologic changes showed that BV6 was toxic to HS5 cells but not A4 and B3 (Fig. 2D). These results suggest an important role of c-IAP1 in maintaining survival of normal cells but not neuroblastoma cells, with neuroblastoma cells exhibiting specific addiction to XIAP for their survival. The dependence of neuroblastoma cells on XIAP, render them more sensitive to XIAP-targeting. Taken together, our findings demonstrated that the loss of XIAP expression mediated by XIAP-specific antagonists A4 and B3—not pan-IAP antagonist BV6—is highly selective and is necessary and sufficient to induce apoptosis in high-risk neuroblastoma cells.

To investigate whether XIAP is indeed a target of A4- and B3-mediated apoptosis, XIAP was overexpressed in KELLY cells to evaluate whether this could blunt apoptosis induced by A4 or B3 treatment (Fig. 2E; Supplementary Fig. S1A). Decreased levels of cleaved PARP and cleaved caspase-3 in treated cells overexpressing XIAP suggest that A4 and B3 are indeed dependent on targeting XIAP to induce apoptosis (Fig. 2E). XIAP-overexpressing KELLY cells treated with BV6 did not undergo apoptosis, similar to control cells (Fig. 2E; Supplementary Fig. S1A). This is unsurprising because XIAP is less sensitive to BV6 (Fig. 2B). This renders the high XIAP-expressing cells resistant to BV6, with or without XIAP overexpression. Intriguingly, NLF, a low XIAP-expressing, *MYCN*-amplified neuroblastoma cell line, was responsive to all three antagonists which could be mitigated by XIAP overexpression (Fig. 2E). More tellingly, SK-N-AS, a non–*MYCN*-amplified neuroblastoma cell line with corresponding low XIAP expression was also responsive to all three antagonists (Supplementary Fig. S1B). This suggests that BV6 is able to reduce XIAP expression but with less potency than A4 and B3, and may explain why certain neuroblastoma cell lines remain sensitive to BV6 while lines with high XIAP and MYCN expression only respond to A4 and B3. Taken together, our results strongly suggest XIAP as a viable target for the suppression of high-risk, resistant neuroblastoma.

### Binding and Degradation of XIAP by A4 is Necessary for Targeting High-risk Neuroblastoma Cells

**FIGURE 3:**
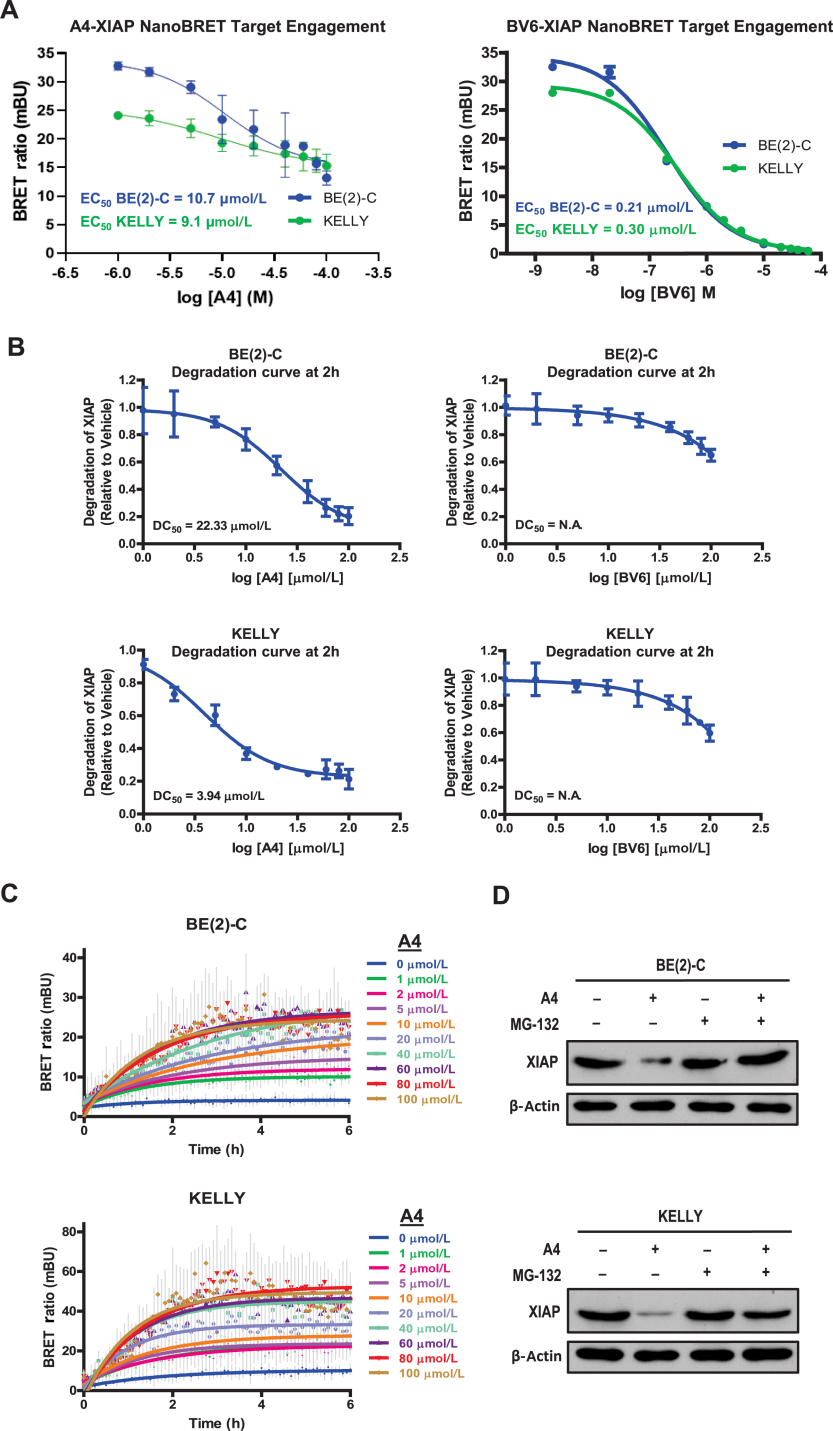
Binding and degradation of XIAP by A4 is necessary for targeting high-risk neuroblastoma cells. **A,** BRET response curves which indicate direct binding/target engagement of either XIAP-specific antagonist A4 or pan-IAP antagonist BV6 to XIAP in neuroblastoma cells. Cells were transfected with XIAP NanoLuc fusions and treated with fixed NanoBRET tracer and various concentrations of unlabeled A4 or BV6 as a competitive compound. BRET signal was measured at 2 hours after treatment using a Tecan Infinite 200 Pro equipped with NanoBRET dual-filters (donor 460 nm and acceptor 618 nm) and determined via the formula (BRET ratio = values at 618 nm/values at 460 nm). Raw BRET ratios were then converted to milliBRET units (mBU) and plotted to determine apparent intracellular affinity of A4 or BV6 to XIAP. Values were expressed mean ± SD of three independent experiments conducted in duplicates. **B,** Degradation profiles of endogenous XIAP in response to A4 or BV6 treatment of neuroblastoma cells, BE(2)-C (top) and KELLY (bottom). Cells were treated with increasing dose of A4 or BV6 and luminescence was measured continuously in real time for 2 hours. Luminescence values were normalized to DMSO vehicle control and degradation profiles were generated using GraphPad Prism. Values were expressed as mean ± SD of three independent experiments conducted in duplicates. **C,** BRET response curves which indicate the binding of ubiquitin to XIAP upon treatment with XIAP-specific antagonist A4 in neuroblastoma cells. Neuroblastoma cells with endogenous luciferase-tagged XIAP were transfected with Halo-tag ubiquitin and treated with fluorescent Halo-tag ligand followed by the addition of various concentrations of A4. BRET signal was measured every 5 minutes for 6 hours after treatment using a Tecan equipped with NanoBRET dual-filters (donor 460 nm and acceptor 618 nm) and determined via the formula (BRET ratio = values at 618 nm/values at 460 nm). Raw BRET ratios were then converted to milliBRET units (mBU) and plotted to determine the binding of ubiquitin to XIAP. Values were expressed mean ± SD of three independent experiments. **D,** Immunoblot analysis of XIAP expression upon A4 treatment with (+) or without (−) 10 µmol/L proteasomal inhibitor MG-132 in neuroblastoma cells, BE(2)-C and KELLY. Cells were pretreated with or without MG-132 for 2 hours prior the addition of A4 for 4 hours. β-actin is used as internal control.

Having demonstrated significantly selective responses of neuroblastoma cells to A4 and B3 versus BV6, we hypothesized that these antagonists act by different mechanisms of action. To better understand the mechanisms by which these compounds target XIAP in high-risk neuroblastoma, binding interactions between the antagonists and XIAP were first determined in BE(2)-C and KELLY cells using a NanoBRET quantitative target engagement assay. We decided to focus on A4 for further investigation given its superior efficacy. As shown in Fig. 3A, a dose-dependent decrease in BRET ratio was observed with increasing A4 or BV6 concentrations. This indicates a competitive displacement of the labeled fluorescent tracer from XIAP by A4 or BV6 which suggests direct interaction between A4 and BV6 with XIAP. It was worth noting that BV6 bound more strongly to XIAP than A4 with the following apparent intracellular affinities determined—0.21 µmol/L (BV6) versus 10.7 µmol/L (A4) in BE(2)-C and 0.30 µmol/L (BV6) versus 9.1 µmol/L (A4) in KELLY (Fig. 3A). As the BRET signal was not completely abolished even at saturating A4 concentrations, NMR was conducted to further examine the binding interaction between A4 and XIAP (Supplementary Fig. S2A and S2B). Consistent with the above findings, NMR analysis on ^1^H-^15^N-HSQC spectra of XIAP in the absence or presence of A4 showed a direct interaction between A4 and XIAP with chemical shift perturbations observed in the spectra (Supplementary Fig. S2A and S2B).

Next, to determine whether direct binding of the antagonists to XIAP could lead to changes in XIAP expression as seen in Fig. 2, a highly sensitive method to quantify the degradation of XIAP was performed. Neuroblastoma cells containing endogenous luciferase-tagged XIAP were generated using CRISPR/Cas9 gene editing to knock-in a bioluminescent tag at endogenous loci of XIAP (Supplementary Fig. S3A–S3D). Following treatment of BE(2)-C and KELLY cells with A4 or BV6, the degradation of XIAP under endogenous condition was monitored (Fig. 3B). Real-time monitoring of endogenous XIAP levels in BE(2)-C and KELLY cells revealed a rapid degradation of XIAP upon A4 treatment, taking place within 10–15 minutes posttreatment (Fig. 3B; Supplementary Fig. S4A). The extent of degradation occured in a dose-dependent manner with a quantitative 2-hour DC_50_ of 22.3 µmol/L in BE(2)-C and 3.9 µmol/L in KELLY (Fig. 3B; Supplementary Fig. S4A). In contrast, 2 hours’ exposure to BV6 resulted in minimal degradation of XIAP and changes in XIAP expression was only apparent at higher doses of BV6 and at a later time (Fig. 3B; Supplementary Fig. S4B).

As rapid degradation of proteins is often catalyzed by the ubiquitin-proteasome system (UPS), we sought to determine whether the UPS is involved in the rapid degradation of XIAP upon treatment with A4. Using a NanoBRET quantitative ubiquitination assay to examine the binding of ubiquitin to XIAP upon treatment with A4, neuroblastoma cells were observed to exhibit a clear dose-dependent increase in BRET ratio, indicating an increase binding of fluorescent-labeled ubiquitin to the endogenous luminescent-tagged XIAP (Fig. 3C). To determine whether ubiquitination of XIAP leads to its proteasomal degradation, MG-132 was used to inhibit the proteasome. Compared with A4 treatment alone, addition of MG-132 was able to prevent A4-mediated degradation of XIAP (Fig. 3D). This suggested that removal of XIAP by A4 is indeed mediated by proteasomal degradation. On the other hand, treatment with BV6 did not degrade XIAP and addition of MG-132 did not lead to appreciable changes in XIAP (Supplementary Fig. S4C). Taken together, these results demonstrated different mechanisms of XIAP targeting by A4 and BV6—the former binding and degrading XIAP via UPS and the latter binding XIAP without degradation. This suggests that binding and degradation of XIAP is necessary for inducing apoptosis in high-risk neuroblastoma cells.

### Treatment with XIAP-specific Antagonist A4 Prolongs and Improves Overall Survival in High-risk Neuroblastoma PDXs

Having shown that A4 was the most potent compound in suppressing high-risk neuroblastoma *in vitro,* we sought to investigate the effect of targeting XIAP by A4 *in vivo* using PDX models of neuroblastoma. Human primary tumor cells derived from patients with *MYCN*-amplified high-risk neuroblastoma were implanted orthotopically in the retroperitoneal periadrenal space of mice, which modeled the typical anatomic site and microenvironment of human disease. Four weeks after tumor implantation, PDXs were then subjected to A4 treatment for pharmacokinetic and survival analysis.

**FIGURE 4:**
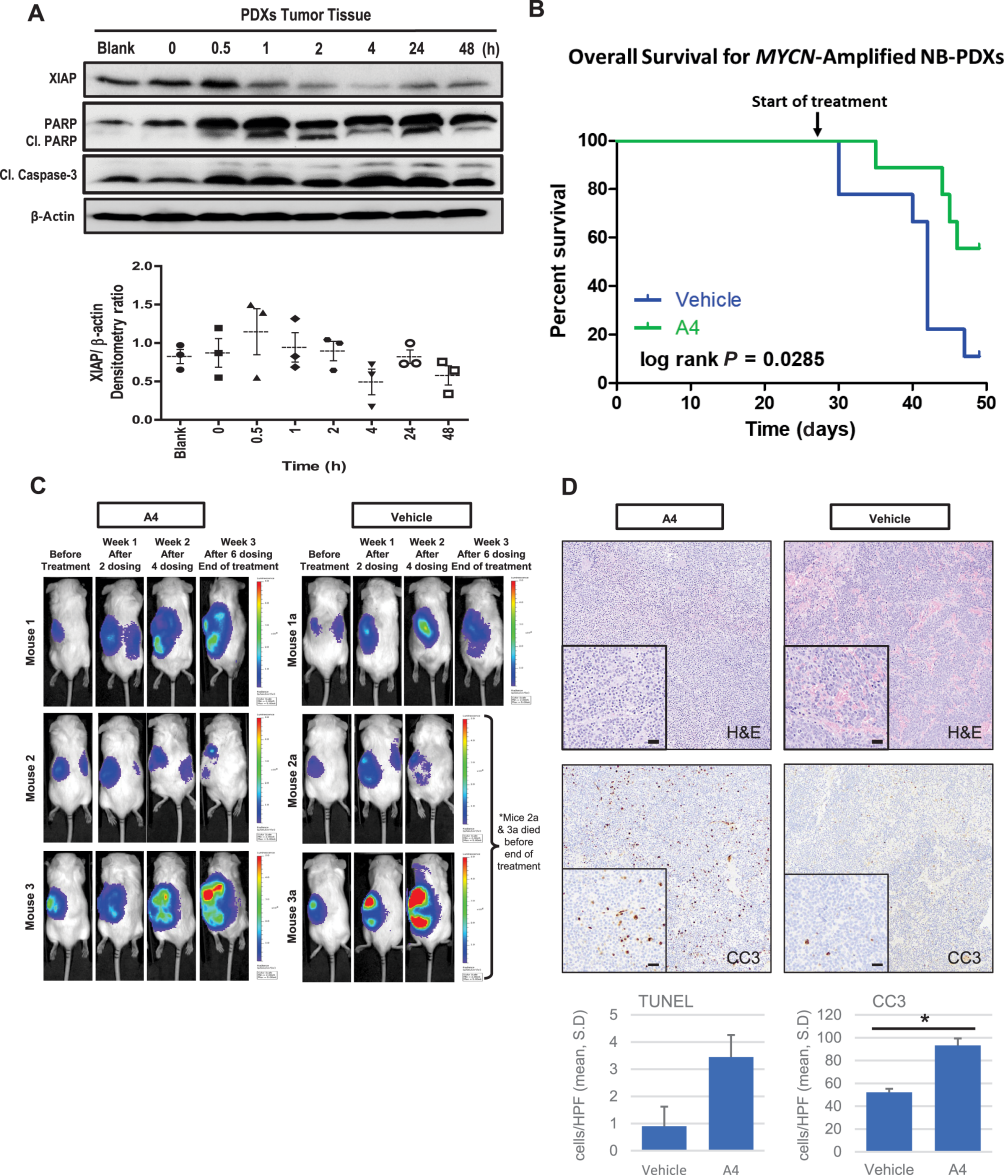
Treatment with XIAP-specific antagonist A4 prolongs and improves overall survival in high-risk neuroblastoma PDXs. **A,** Top, Immunoblot analysis of XIAP, cleaved PARP and cleaved caspase-3 expression in tumors harvested from treated neuroblastoma PDXs. Neuroblastoma PDXs were treated with 10 mg/kg A4 via intraperitoneal injection. Tumors were harvested over the following time courses of 0, 0.5, 1, 2, 4, 24, and 48 hours after dosing. β-actin is used as internal control. Bottom, Graphical representation of densitometry ratio for XIAP expression normalized to β-actin in tumors harvested from neuroblastoma PDXs as described in A (Supplementary Data S2). Values were expressed as mean ± SD with *n* = 3/timepoint. **B,** Kaplan–Meier survival plot of *MYCN*-amplified neuroblastoma PDXs (*n* = 9/treatment group). Blue line indicates percentage survival of vehicle-treated mice (control) and green line is the percentage survival of A4-treated mice (10 mg/kg). The mice were monitored over a period of 7 weeks, including 4 weeks of stabilizing implantation and subsequent 3 weeks of treatment period. log-rank (Mantel–Cox test) derived from Kaplan–Meier survival plot was used for statistical comparisons between groups with *, *P* < 0.05. **C,** Bioluminescence imaging of tumor growth in two representative neuroblastoma PDXs. **D,** Top, Representative photomicrographs of A4-treated and vehicle control tumors demonstrating increased intratumoral hemorrhage in the latter [pink eosinophilic areas, hematoxylin and eosin (H&E), 100X and 200X (inset)], and increased staining for cleaved caspase 3 in the former [CC3, 100X, and 200X (inset)]. Bottom, Average cells per HPF with positive staining on TUNEL assay and CC3 IHC in A4-treated and vehicle control tumors, showing significantly increased CC3 staining in A4-treated tumors (*n* = 5 tumors per group, average of 5 HPF per tumor, *P* = 0.03, paired *t* test; scale bar: 50 µm).

Pharmacokinetic analysis of 10 mg/kg A4 delivered via intraperitoneal injection showed good exposure of A4 in mouse plasma and tumor with a maximum modeled concentration (Cmax) of 600 and 4,000 nmol/L, respectively (Supplementary Fig. S5A and S5B). Tumors harvested from neuroblastoma PDXs after exposure to 10 mg/kg A4 at serial timepoints over 48 hours showed an eventual reduction of XIAP tissue expression and increased apoptosis indicated by PARP and caspase-3 cleavage, consistent with *in vitro* findings (Fig. 4A; Supplementary Fig. S6A and S6B). Also, mice receiving 10 mg/kg A4 showed no substantial weight loss or adverse effects with twice-weekly dosing for up to 3 weeks (Supplementary Data S3; Supplementary Fig. S6C). This suggested that 10 mg/kg could be a clinically suitable and sufficient dose for evaluating the potential anticancer effect of A4 in neuroblastoma PDX models.

To determine the clinical potential of A4 on high-risk neuroblastoma, PDXs generated after 4 weeks of tumor implantation were randomly distributed into treatment (10 mg/kg A4) and vehicle (DMSO; 1 mL/kg) groups, and treated for 3 weeks. Kaplan–Meier curves of *MYCN*-amplified neuroblastoma PDXs showed a significantly longer overall survival in the treatment group, with more than 50% of mice alive at the end of the treatment period (Fig. 4B). In contrast, majority of the mice in the vehicle control group did not survive (Fig. 4B). Despite the prolonged overall survival in the treatment group, there was no observable difference in tumor size between treatment and vehicle control groups (Fig. 4C). Histologic examination revealed that vehicle control tumors had substantially greater areas of intratumoral hemorrhage indicative of rapid tumor cell proliferation, while A4-treated tumors showed increased positive staining for cleaved caspase 3 and TUNEL IHC (Fig. 4D). This suggested that A4 as a single agent *in vivo* can extend survival at the current dose, with the potential to be a novel anti-neuroblastoma drug that could work well in combination with existing therapies.

### XIAP-specific Antagonist A4 Works Synergistically with and Promotes Effective Dose Reduction of Vincristine and Topotecan *In Vitro*

For treatment of high-risk neuroblastoma, current standard-of-care utilizes intensive multimodal therapy involving combinations of multiple cytotoxic agents. Because treatment with A4 as a single agent did not substantially suppress tumor growth despite prolonging and improving survival in neuroblastoma PDXs, we sought to evaluate the synergistic potential of A4 in combination with current standard-of-care cytotoxic agents, vincristine and topotecan, for the treatment of high-risk neuroblastoma. To analyze potential drug interactions, treatment of BE(2)-C and KELLY cells with A4 in combination with either vincristine or topotecan was examined. Using Chou–Talalay method, the drug interaction effect/type determined by the drug combination (CI) index is as follows: CI <1 (synergistic effect); CI = 1 (additive effect); CI >1 (antagonistic effect). The effective dose reduction of each drug when used in combination determined by the DRI is as follows: DRI<1 (unfavorable dose reduction); DRI > 1 (favorable dose reduction and potential toxicity reduction; ref. 25).

**FIGURE 5:**
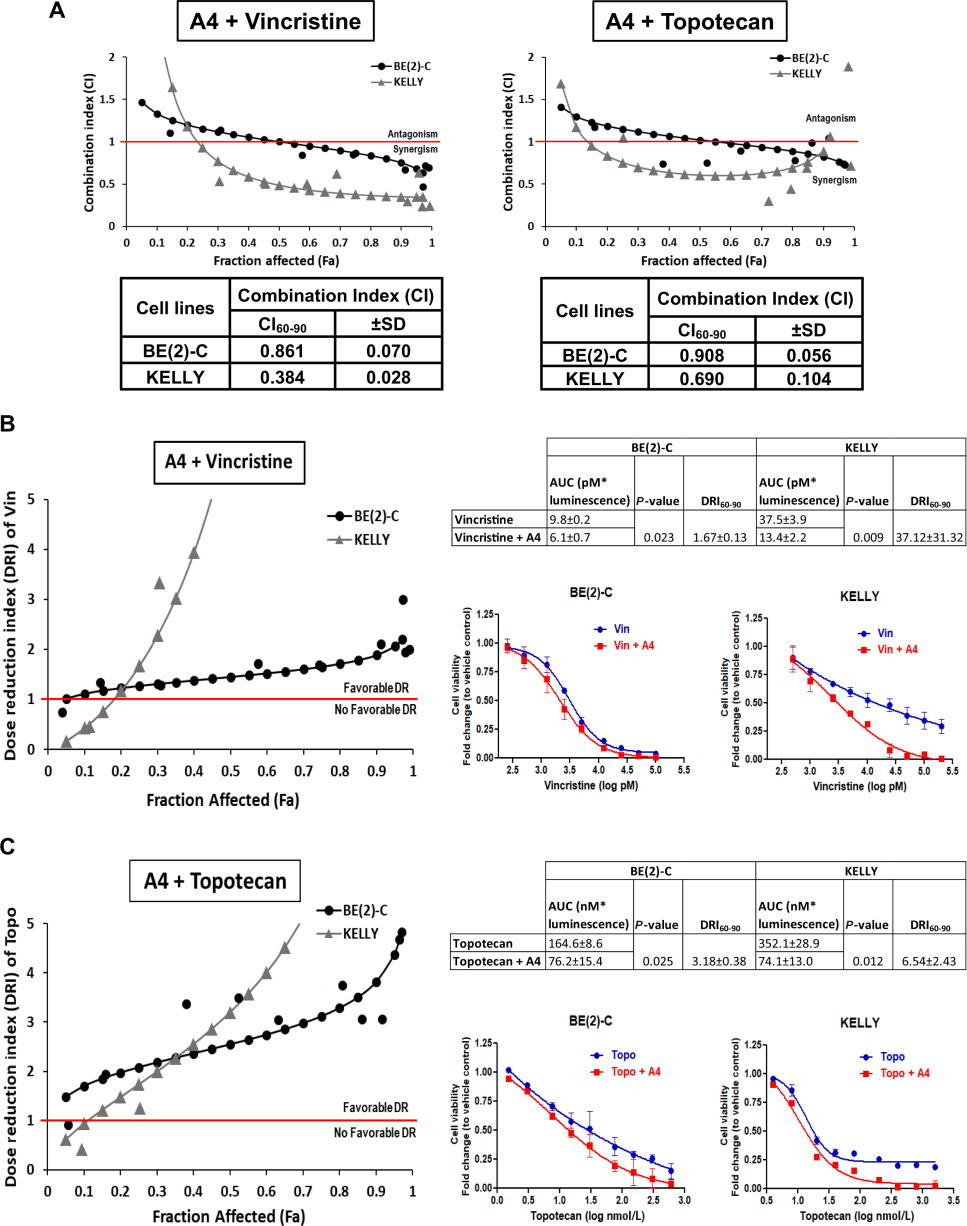
XIAP-specific antagonist A4 works synergistically with and promotes effective dose reduction of vincristine and topotecan *in vitro*. **A,** CI demonstrating synergism between A4 with vincristine and topotecan. Increasing doses of A4 with either vincristine or topotecan were added to neuroblastoma cell lines at a fixed ratio based on the IC_50_ values of the individual drugs for 48 hours [A4:vincristine; BE(2)-C 1:1 (0–100 µmol/L A4: 0–100 nmol/L Vin), KELLY 1:0.5 (0–100 µmol/L A4: 0–50 nmol/L Vin) and A4:Topotecan; BE(2)-C 1:6 (0–100 µmol/L A4: 0–600 nmol/L Topo), KELLY 1:16 (0–100 µmol/L A4: 0–1,600 nmol/L Topo)], respectively. CI was generated using CompuSyn software by Chou–Talalay (25). CI_60–90_ represents the average CI at 60%–90% cell death. CI <1 denotes synergistic effect; CI = 1 denotes addictive effect and CI >1 denotes antagonistic effect. DRI demonstrating the fold differences of A4 effectively reducing the dose of vincristine (**B**) or topotecan (**C**) when used in combination with these agents. Increasing doses of A4 with either vincristine or topotecan were added to neuroblastoma cell lines at a fixed ratio based on the IC_50_ values of the individual drugs for 48 hours (A4:vincristine; BE(2)-C 1:1, KELLY 1:0.5 and A4:Topotecan; BE(2)-C 1:6, KELLY 1:16), respectively. DRI was generated using CompuSyn software by Chou–Talalay. DRI_60–90_ represents the average DRI at 60%–90% cell death. DRI <1 denotes unfavorable dose reduction and DRI >1 denotes favorable dose reduction. Right, Dose–response curves of vincristine(B) and topotecan (C) treated alone or in combination with A4 in BE(2)-C and KELLY neuroblastoma cells, with corresponding comparison of mean areas under the dose–response curves with versus without addition of A4 (*t* test).

Combination of A4 and vincristine revealed a synergistic interaction in BE(2)-C and KELLY, with an average CI at 60%–90% cell death (CI_60–90_) of 0.861 ± 0.070 and 0.384 ± 0.028, respectively (Fig. 5A). Similarly, a synergistic interaction between A4 and topotecan was also detected in the same cell lines, although with a lower average CI_60–90_ of 0.908 ± 0.056 in BE(2)-C and 0.690 ± 0.104 in KELLY (Fig. 5A). Together, these findings showed that A4 works synergistically when used in combination with either vincristine or topotecan in neuroblastoma cells.

Furthermore, when used in combination with these cytotoxic agents, A4 promoted an effective dose reduction of vincristine, with an average DRI at 60%–90% cell death (DRI_60–90_) of 1.674 ± 0.129 in BE(2)-C and 37.120 ± 31.315 in KELLY (Fig. 5B). This was depicted by the left-shift in dose–response curves, indicating a reduction of vincristine dose in cells treated with A4 and vincristine, compared with vincristine alone (Fig. 5B). Areas under the dose–response curves were significantly reduced with A4 and vincristine compared with vincristine alone, in both BE(2)-C and KELLY cell lines (*P* = 0.0023 and 0.009, respectively). Similarly, when A4 was used in combination with topotecan, it promoted an effective dose reduction of topotecan with an average DRI_60–90_ of 3.183 ± 0.378 in BE(2)-C and 6.537 ± 2.430 in KELLY (Fig. 5C). Compared with cells treated with topotecan alone, a left-shift in dose–response curves of cells treated with A4 and topotecan was also observed. Areas under the dose–response curves were significantly reduced with A4 and topotecan compared with topotecan alone, in both BE(2)-C and KELLY cell lines (*P* = 0.0025 and 0.0012, respectively). Interestingly, vincristine or topotecan promoted an effective dose reduction of A4 in BE(2)-C and KELLY as well (Supplementary Fig. S7A and S7B). In contrast, only additive effect was observed for vincristine in SK-N-AS, a non–*MYCN*-amplified and low XIAP-expressing cell line, representative of low-risk disease (Supplementary Fig. S7A and S7B). This is unsurprising, given SK-N-AS moderately lesser sensitivity to A4 than BE(2)-C and KELLY. In most cases, low-risk disease does not require chemotherapy. Taken together, XIAP-specific antagonist A4 not only prolongs and improves the overall survival of high-risk neuroblastoma PDXs as a single agent, but also shows translational potential to augment the efficacy of cytotoxic agents in killing high-risk neuroblastoma cells when used in combination.

## Discussion

Neuroblastoma is one of several neural crest–derived cancers that share a common developmental biology requiring XIAP. Reduced XIAP expression is necessary for developmental apoptosis to initiate. Hence, tumor-initiating cells of prenatal embryonal cancers including neuroblastoma which fail to undergo developmental apoptosis are often found to have high XIAP expression, suggesting a dependency on XIAP for survival (10, 31). Despite the key role played by XIAP in neural crest development, the translational potential of exploiting XIAP antagonism as a treatment strategy for neuroblastoma has not been investigated.

In the current study, we showed that aggressive, high-risk *MYCN-*amplified neuroblastoma cells tend to express a higher level of XIAP. Interestingly, such an association of XIAP expression with high-risk features of disease had also been observed in previous studies. For instance, the upregulation of XIAP expression, but not other apoptotic regulators, has been associated with high-risk biological features and poor survival in acute myeloid leukemia as well as other neuroectodermal cancers like melanoma, gastrointestinal, and pulmonary neuroendocrine tumors—all highly aggressive malignancies with poor prognoses (12, 13, 32, 33). We also demonstrated that the silencing of XIAP in high XIAP-expressing and *MYCN*-amplified neuroblastoma cells resulted in significant apoptosis, suggesting the addiction and dependency on XIAP for survival, thus highlighting the potential of targeting XIAP as a treatment strategy for high-risk neuroblastoma.

We tested several small-molecule compounds targeting pan-IAPs or XIAP alone. SMAC mimetics with pan-IAP targeting activity have been widely studied and tested in many cancers while XIAP-specific targeting is a new treatment strategy not previously explored in neuroblastoma. We found that the ARTS mimetic A4, an XIAP-specific antagonist, demonstrated the best efficacy across a panel of commercial and patient-derived neuroblastoma cell lines. It was especially effective against high XIAP-expressing cell lines which also happened to be *MYCN-*amplified, a classical hallmark of aggressive and resistant neuroblastoma. Moreover, its efficacy appeared to be stratified according to XIAP and N-Myc expression status. A4 was also best tolerated by normal cell lines representing liver and bone marrow, which are common sites of neuroblastoma metastasis. Furthermore, our study provides the first proof-of-concept on the efficacy of XIAP-specific antagonist against neuroblastoma tumors *in vivo*, having determined the murine pharmacokinetic profile of A4 and demonstrated its utility in PDX models. Encouragingly, A4 remained stable with good availability in plasma, and was shown to prolong and improve the overall survival of high-risk neuroblastoma PDXs at the tested dose of 10 mg/kg. Though tumors did not show gross regression in size when A4 was used as a single agent, A4 showed evidence for synergism with standard-of-care cytotoxic agents in neuroblastoma cells representative of high-risk disease. Notably, A4 was able to reduce the doses of these cytotoxic agents when used in combination. This offers potential benefit in toxicity reduction if used in the treatment of high-risk neuroblastoma which currently involves intensive multimodal therapy. In contrast, we found that pan-IAP antagonists tested in this study including LCL-161, CUDC-427, Debio1143, and BV6 targeted mainly c-IAPs, induced high toxicity in normal cells and were ineffective toward XIAP-dependent*, MYCN*-amplified neuroblastomas.

A4 demonstrates promising efficacy in prolonging the survival of high-risk, *MYCN*-amplified neuroblastoma PDXs despite having weaker binding affinity for XIAP than Smac mimetic BV6. This indicates that the mechanism of action for A4 is distinct, and that degradation of XIAP is more important for therapeutic success than binding-mediated inhibition. Notably, both ARTS and A4 promote degradation of XIAP, whereas Smac and Smac-mimetics do not. This finding lends support to the development of more potent derivatives from first-generation A4.

Intriguingly, we showed that the ARTS mimetic A4 predominantly degrades XIAP while SMAC mimetic BV6 inhibits XIAP. Similar results were shown for other cancers (21). This difference in mechanism for suppressing XIAP resulted in different responses in neuroblastoma cells. Notably, similar observations have been described for receptor tyrosine kinase (RTK) targeting, where inhibitors had to be sustained at saturating concentration for extended periods to effect signaling suppression while equivalent effects could be achieved in a much shorter time via compounds that degrade RTK (34). It was thus postulated that XIAP could have another functional role in protecting from apoptosis, independent from the one disrupted by SMAC binding. Hence, besides the main binding pocket bound by SMAC mimetic BV6 (the IBM motif, located at the BIR3 domain of XIAP, also known as the SMAC-binding pocket), other XIAP domains could also exhibit functionality. In this regard, the RING domain of XIAP has previously been shown to function as an E3 ubiquitin ligase involved in ubiquitination (35). Upon release from the mitochondria, the main function of SMAC is to bind and inhibit XIAP. However, instead of inhibiting XIAP, XIAP-bound SMAC has been shown to be targeted by XIAP for ubiquitination and subsequent, degradation, thereby freeing XIAP (36). Therefore, the ability of XIAP to be bound and yet still catalyze ubiquitination suggests that XIAP is still functional even when bound by its endogenous inhibitor. This possibility is supported by a recent study on the development of specific nongenetic IAP-based protein erasers (SNIPER; ref. 37). SNIPERs consist of an IAP-binding site to the BIR3 domain and a ligand for the target protein. Using its IAP-binding site, SNIPERs are able to bind to IAPs and bring the target protein into close proximity for degradation by utilizing the E3 ligase function of IAPs’ RING domain. Indeed, XIAP could still be functionally protective in spite of binding to compounds that disrupt its binding and inhibition of caspases. However, this function remains to be investigated. In addition, the minimal or lack of XIAP degradation induced by BV6 could be attributed to its preferential binding to c-IAPs (20, 38). This could have resulted in sequestration of BV6 from binding and targeting XIAP and thus, triggering less apoptotic killing. To overcome the effects of sequestration, a higher dose would be required but may not be feasible due to the toxicity of BV6 to noncancerous cells.

**FIGURE 6:**
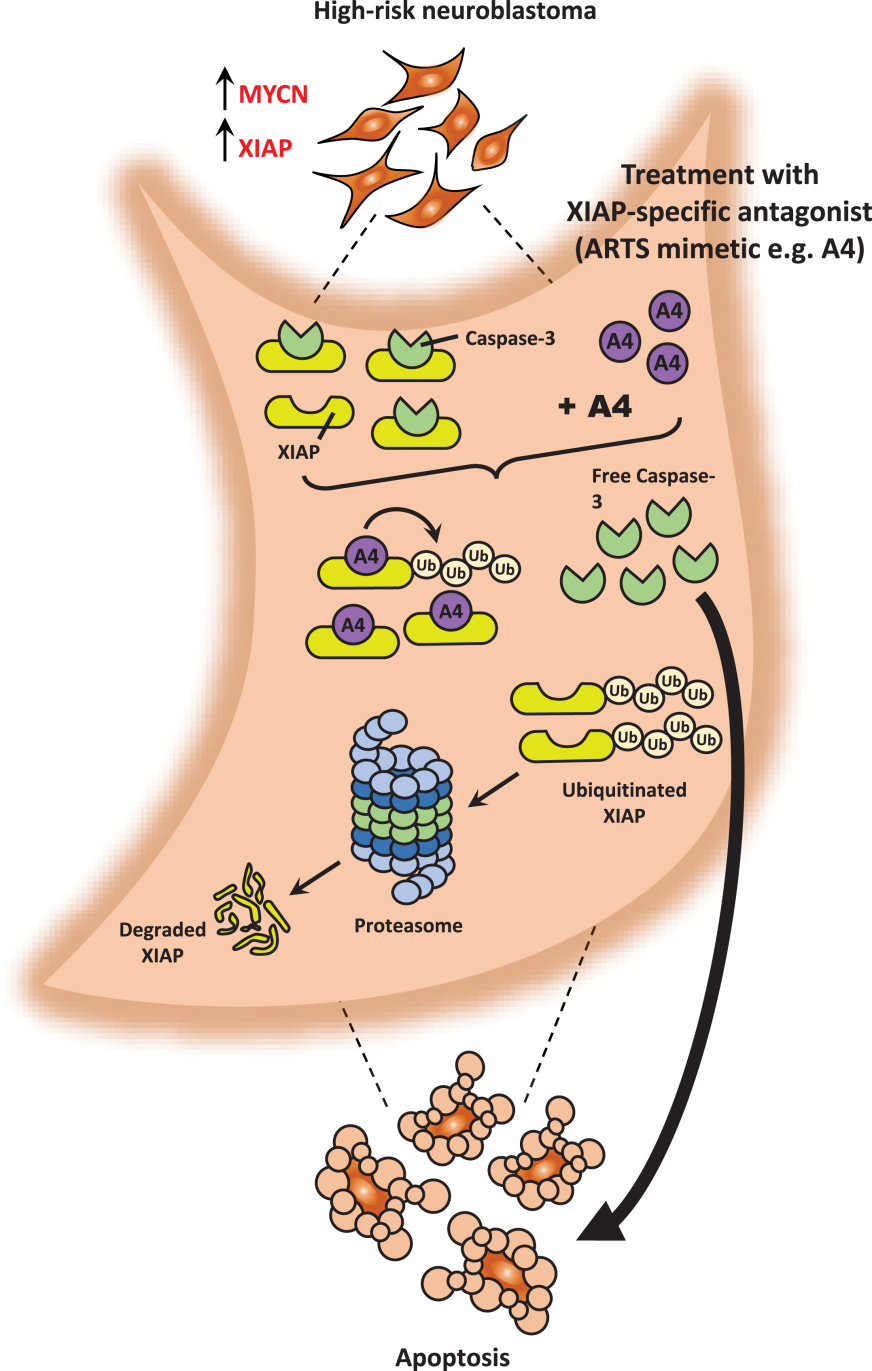
Mechanisms mediated by various IAP antagonists, pan-IAP antagonist (SMAC mimetic) BV6, and XIAP-specific antagonist (ARTS mimetic) A4 in high-risk neuroblastoma. In high-risk neuroblastoma, *MYCN* amplification is frequently associated with high XIAP expression. Given their dependency on XIAP for survival, these tumor cells are highly sensitive to treatment with A4 but not BV6. A4 binds and triggers the ubiquitination and rapid degradation of XIAP resulting in the release of caspases which execute effective apoptosis. (1) Conversely, BV6 preferentially binds c-IAPs over XIAP, resulting in possible sequestration of BV6 by c-IAPs and less caspase being freed from XIAP. BV6 rapidly triggers the autoubiquitination and degradation of c-IAPs which have minimal effect on neuroblastoma tumor cells. Furthermore, the binding and inhibition of XIAP by BV6 was also less effective in triggering apoptosis. (2) BV6-bound XIAP could still function independently on the inhibition binding site, (2a) thus allowing XIAP to bind to other proteins that could mediate antiapoptotic effects. As a result, overall BV6 mediates less or no apoptosis in these high-risk neuroblastoma tumor cells. Taken together, XIAP-specific antagonists outperform pan-IAP antagonists by specifically degrading and not inhibiting XIAP, thus presenting this as a potential novel treatment strategy for high-risk neuroblastoma.

In summary, binding and degradation of XIAP by ARTS mimetics is a novel and effective therapeutic strategy against high-risk neuroblastoma either alone or in combination with current standard-of-care agents (Fig. 6). Better understanding of this unique mechanism of XIAP degradation may facilitate the development of compounds with improved potency and efficacy than A4.

## Supplementary Material

Supplementary Methods Supplementary Figure Legends, Methods and Resources

Supplementary Data 1 Supplementary Data 1 to Fig 1B

Supplementary Data 2 Supplementary Data to Fig 4A

Supplementary Data 3 Supplementary Data 3 to Fig 4B

Supplementary Data - raw Western blot images Supplementary Data - raw Western blot images

Table S1 Supplementary Table S1, related to Figure 1. Table of densitometry values calculated from western blot to generate scatter plot in Figure 1A.

Table S2 Supplementary Table S2, related to Figure 2. Table of IC50 (μM) indicating the effect of individual IAP antagonists on viability of neuroblastoma and normal tissue-derived cells measured every 24 hours up to 72 hours.

Figure S1 Supplementary Figure S1, related to Figure 2. Importance of XIAP expression level in mediating sensitivity of neuroblastoma cells to A4, B3 and BV6.

Figure S2 Supplementary Figure S2, related to Figure 3. NMR analysis on 1H-15N-HSQC spectra of XIAP in the absence and presence of A4.

Figure S3 Supplementary Figure S3, related to Figure 3. Generation of luciferase-tagged XIAP using CRISPR knock-in gene editing.

Figure S4 Supplementary Figure S4, related to Figure 3. Time course of XIAP degradation in response to XIAP-specific (A4) and pan-IAP (BV6) antagonists.

Figure S5 Supplementary Figure S5, related to Figure 4. Pharmacokinetic profile of XIAP-specific antagonist A4.

Figure S6 Supplementary Figure S6, related to Figure 4. Immunoblot analysis of tumor tissue from other sets of PDXs treated with A4.

Figure S7 Supplementary Figure S7, related to Figure 5. Vincristine or topotecan works synergistically with and promotes effective dose reduction of XIAP-specific antagonist A4 in vitro.
