# Supplementary material for: Targeted Degradation of XIAP is Sufficient and Specific to Induce Apoptosis in MYCN-overexpressing High-risk Neuroblastoma
Source: Cancer Res Commun. 2023 Nov 22;3(11):2386–99. doi: 10.1158/2767-9764.CRC-23-0082 (PMC10681007; doi:10.1158/2767-9764.CRC-23-0082)
Supplement: Supplementary Methods — Supplementary Figure Legends, Methods and Resources [file crc-23-0082-s01.docx]

**Supplementary Figure Legends**

**Figure S1, related to Figure 2. Importance of XIAP expression level in mediating sensitivity of neuroblastoma cells to A4, B3 and BV6.**

**(A)** Graphical representation showing the fold change of caspase-3/7 activity relative to vehicle controls for pcDNA3 and overexpressed XIAP in KELLY neuroblastoma cells after treatment with 10 μM of either A4, B3 or BV6 at indicated timings (data were expressed as mean of one independent experiment conducted in duplicates). **(B)** Immunoblot analysis of c-IAP1, XIAP, PARP and cleaved caspase-3 expression in neuroblastoma cell line, SK-N-AS, after treatment with 10 µM of either A4, B3 or BV6 at indicated timings (three independent replicates).

**Figure S2, related to Figure 3. NMR analysis on 1H-15N-HSQC spectra of XIAP in the absence and presence of A4.**

**(A)** Assignment of the 1H-15NHSQC spectrum of XIAP. The assigned cross peaks in the spectrum were labeled with residue name and sequence number**.** **(B)** The ^1^H-^15^N-HSQC spectra of XIAP in the absence (black) and presence (red) of A4. The spectra were collected as described in Materials and Methods. Residues with chemical shift perturbations after A4 treatment were indicated with residue name and sequence number.

**Figure S3, related to Figure 3. Generation of luciferase-tagged XIAP using CRISPR knock-in gene editing.**

**(A)** Schematic representation of XIAP structure containing HiBiT and the combination with LgBiT resulted in active functional luciferase producing luminescence. **(B)** Graphical representation of luminescence measured in neuroblastoma cells, KELLY and BE(2)-C after CRISPR knock-in of HiBiT, a small subunit of highly sensitive luciferase. Three guide RNAs were used for optimization. Signal >3 folds represent positive signal. **(C)** Agarose gel analysis of neuroblastoma cells containing HiBiT using guide RNA 1. The detection of bands was performed via multiplex PCR using three primers as described in materials and methods. Presence of HiBiT was indicated by the presence of two bands at size 211bp and 139 bp. **(D)** Graphical representation of luminescence measured in neuroblastoma cells after stably transfection with large subunit LgBiT. The combination of HiBiT and LgBiT forms the active luciferase which produces luminescence. The absence of either subunit would result in negative luminescent signal.

**Figure S4, related to Figure 3. Time course of XIAP degradation in response to XIAP-specific (A4) and pan-IAP (BV6) antagonists.**

**(A)** Degradation profiles of endogenous XIAP in response to A4 treatment of neuroblastoma cells, BE(2)-C and KELLY. **(B)** Degradation profiles of endogenous XIAP in response to BV6 treatment of neuroblastoma cells, BE(2)-C and KELLY. Bottom – Degradation curve of BV6 at 8 hours. Cells were treated with increasing dose of A4 or BV6 and luminescence was measured continuously in real-time for 2 hours (A4) or 8 hours (BV6), respectively. Luminescence values were normalized to DMSO vehicle control and degradation profiles were generated using GraphPad Prism. Values were expressed as mean ± SD of three independent experiments. **(C)** Immunoblot analysis of XIAP expression upon BV6 treatment with (+) or without (-) 10 μM proteasomal inhibitor MG-132 in neuroblastoma cells, KELLY and BE(2)-C. Cells were pre-treated with or without MG-132 for 2 hours prior the addition of BV6 for 4 hours. β-actin is used as internal control.

**Figure S5, related to Figure 4. Pharmacokinetic profile of XIAP-specific antagonist A4.**

**(A-B)** Pharmacokinetic curve demonstrating the concentrations of A4 in mice plasma (A) and tumor (B) over time (mean ± SD). Neuroblastoma PDXs were treated with 10 mg/kg A4 via intraperitoneal injection. 150-200 μL of blood was taken from each mouse via cardiac puncture and tumors were harvested over the following time courses of 0, 0.5-, 1-, 2-, 4-, 24- and 48-hours post-dosing (n=3/time point). The concentrations of A4 in mice plasma were determined by LCMS/ MS in MRM positive mode.

**Figure S6, related to Figure 4. Immunoblot analysis of tumor tissue from other sets of PDXs treated with A4.**

**(A-B)** Immunoblot analysis of XIAP, cleaved PARP and cleaved caspase-3 expression in tumors harvested from treated neuroblastoma PDXs. Neuroblastoma PDXs were treated with 10 mg/kg A4 via intraperitoneal injection. Tumors were harvested over the following time courses of 0, 0.5, 1, 2, 4, 24 and 48 hours post-dosing. β-actin is used as internal control. **(C)** Average weight of vehicle-or A4-treated mice over the course of treatment.

**Figure S7, related to Figure 5. Vincristine or topotecan works synergistically with and promotes effective dose reduction of XIAP-specific antagonist A4 *in vitro*.**

**(A-B)** Right – Dose reduction index (DRI) demonstrating the fold differences of (A) vincristine or (B) topotecan effectively reducing the dose of A4 when used in combination. DRI was generated using CompuSyn software by Chou-Talalay. DRI_60-90_ represents the average dose reduction index at 60-90% cell death. DRI <1 denotes unfavorable dose reduction and DRI >1 denotes favorable dose reduction. Left – Dose response curves of A4 treated alone or in combination with (A) vincristine and (B) topotecan in BE(2)-C, KELLY and SK-N-AS neuroblastoma cells.

**Supplementary Methods**

**Western blotting**

Whole cell lysates harvested were lysed in EBC buffer (pH 8.0 of 50 mM Tris, 0.5% NP-40, 120 mM sodium chloride) containing protease inhibitors and protein concentration were quantified using the Bradford assay (Thermo Fisher Scientific). 60 -100 µg of proteins were separated by 12% gel SDS-PAGE followed by a wet-transfer onto PVDF membranes (BioRad). Membranes were probed overnight at 4°C with specific primary antibodies as stated in key resources table. Detection was performed using X-ray film with enhanced chemiluminescence method (Merck) Densitometric analysis of the blots for quantification of protein expression ratios was performed using ImageJ software.

**Tissue microarray (TMA), immunohistochemistry and fluorescence in-situ hybridization (FISH)**

TMA analysis was performed on neuroblastoma patients’ samples obtained from KK Women’s and Children’s Hospital. Ethical permission was obtained from the SingHealth Central Institutional Review Board (CIRB 2012/450/F, 2019/2136). TMAs of tumor specimens were constructed in triplicate from formalin-fixed paraffin-embedded (FFPE) tissue blocks using a 1 mm-wide diameter punch (Estigen) and a manual tissue-arraying instrument (Beecher Instruments). 4 µm-thick unstained sections of TMA blocks were treated with high pH H2 buffer (Leica Biosystem) for 20 minutes and stained with anti-XIAP antibody (sc-55550) (Santa Cruz Biotechnology) at dilution 1:500. DAB substrate was used as the chromogen and nuclei were counterstained with hematoxylin. The expression of XIAP on TMA was reviewed and scored by pathologist; with a scoring of 0 represents negative/null XIAP expression and a highest scoring of 3 represents high XIAP expression. Scoring results were tabulated and analyzed with the patients’ underlying clinical information

For each of the tumor specimens in the TMA, FISH assays were carried out on representative FFPE tumor sections selected by a pediatric pathologist, using MYCN SpectrumGreen and CEP2 SpectrumOrange DNA probes (Abbott Molecular). FFPE slides were baked at 56 °C overnight, and deparaffinized in xylene and 100% ethanol. Enzymatic digestion was carried out with Protease I Solution (Abbott Molecular), DNA probe mixture was applied to the target area and co-denatured, and hybridization carried out overnight at 37 °C. The slides were analyzed under an epifluorescence microscope and captured and processed using Isis software (Metasystems GmbH)

**Clonogenic assays**

200,000 cells were seeded in 6-well plate on the day of lentiviral transduction. Cells were transduced with lentivirus encoding either control or targeting XIAP, followed by 24 hours incubation and subsequent selection by puromycin for two weeks. Stable-selected cells were then harvested for staining with crystal violet containing methanol followed by repeated washing with water and drying. Pictures of stained cells were taken using a Brother DCP-L2540DW scanner.

**Lentivirus production and infection**

Lentiviruses targeting XIAP were generated by transfecting 293FT cells with sh*XIAP*-encoding plasmid (Sigma) and 3^rd^ generation lentiviral packaging plasmids (pLP1, pLP2, and pLP/VSVG) using Lipofectamine® 2000 (Thermo Fisher Scientific). Supernatants containing the lentivirus were collected and pelleted. sh*SCR* plasmid encoding non-targeting virus (SCR) was used as a negative control. sh*XIAP* and sh*SCR* plasmids were purchased from Sigma-Aldrich MISSION® shRNA libraries with the sequences stated in key resources table. Lentivirus produced was used to infect neuroblastoma cells followed by downstream experiments of western blotting, clonogenic and apoptotic assays.

**Generation of luciferase-tagged neuroblastoma cells using CRISPR/Cas-9 gene editing (Knock-in)**

Endogenous-tagging of XIAP with luciferase was performed using Promega’s NanoBiT luciferase technology. The NanoBiT luciferase consists of two subunits – HiBiT and LgBiT. HiBiT was first introduced at endogenous XIAP locus using CRISPR knock-in gene editing following Promega manufacturer’s instructions. Alt-R® S.p. Cas9 Nuclease V3, Alt-R® transactivating CRISPR RNA (tracrRNA), Alt-R® CRISPR RNA (crRNA), Ultramer single-stranded oligo DNA nucleotides (ssODN), and nuclease-free duplex buffer were purchased from Integrated DNA Technologies (6). The sequences used for CRISPR knock-in can be found in key resources table. Guide RNA (gRNA) was prepared by 5 minutes 95°C heating of reaction consisting of 1200 pmol of each crRNA and tracrRNA, and nuclease-free duplex buffer in a final volume of 50 μL, followed by cooling to room temperature. Ribonucleoprotein (RNP) complexes were prepared by incubating 120 pmol gRNA and 100 pmol Cas9 in a final volume of 10 μL for 10 minutes at room temperature. For nucleofection of neuroblastoma cells, 2 X 10^5^ cells were first resuspended in 20 μL 4D-Nucleofector reagent (Nucleofector SF Solution + Supplement) (Lonza) with the subsequent addition of 2.5 μL RNP complex and 100 pmol donor ssODN. The reaction was then subjected to electroporation with the Lonza 4D Nucleofector using program CA-137 for BE(2)-C and CM-130 for KELLY. Each sample was warmed and incubated with growth medium for 20 minutes before transferring the cell suspension to a 24-well plate. After recovering for a few days, the cells were assayed for HiBiT insertion using Nano-Glo HiBiT lytic detection system from Promega to determine the bioluminescence via Tecan Infinite®200 Pro. The detection of more than 3-fold luminescence measurement indicated the presence of HiBiT. The presence of HiBiT at XIAP locus was subsequently confirmed via multiplex PCR using three primers as stated in key resources table. After the confirmation, the HiBiT-containing cells were subsequent subjected to transfection with LgBiT expression vector (Promega) using FuGENE® HD transfection reagent following manufacturer’s instructions. Cells were then incubated for 24 hours, followed by hygromycin B (Thermo Fisher Scientific) selection for 1 to 2 weeks. Stably-transfected cells were harvested and assayed for HiBiT-LgBiT (NanoBiT luciferase) insertion using Nano-Glo live cell substrate from Promega to measure the bioluminescence via Tecan Infinite®200 Pro. Positive signals indicate successful tagging of luciferase to XIAP in neuroblastoma cells. Luciferase-tagged-XIAP neuroblastoma cells were used in subsequent downstream experiments including the monitoring of luminescence changes in real time after treatment with IAP antagonists. Kinetic measuring of bioluminescence was performed using Nano-Glo® Vivazine™ live cell substrate from Promega.

**NanoBRET™ Ubiquitination Assay**

The quantification of XIAP ubiquitination was performed via Promega’s NanoBRET ubiquitination assay. Following manufacturer’s instructions, HaloTag®-Ubiquitin expression vector was first introduced via transient transfection into stably-transfected luciferase-tagged XIAP neuroblastoma cells using FuGENE® HD transfection reagent. 24 hours post-transfection, the cells were replated into white 96-well plate for overnight in the presence or absence of fluorescent NanoBRET™ HaloTag® 618 ligand (which binds specifically for ubiquitin). The cells were then incubated with Nano-Glo® Vivazine™ live cell substrate for 1 hour before treatment with XIAP-specific antagonist, A4. Using luciferase-tagged-XIAP as donor protein and fluorescent Halo-tagged ligand as acceptor, dual-filtered luminescence was measured every 5 minutes for 6 hours using Tecan Infinite®200 Pro (donor at 460 nm and acceptor at 618 nm) and BRET ratio (values at 618 nm / values at 460 nm) was determined. BRET response curve was plotted using GraphPad Prism software after general normalization by subtracting no ligand BRET values.

**NanoBRET™ Target Engagement Assay**

The quantification and determination of XIAP target engagement with test compounds was performed via Promega’s NanoBRET XIAP target engagement assay. Following manufacturer’s instructions, NanoLuc-XIAP expression vector provided in the kit was transiently transfected into neuroblastoma cells using FuGENE® HD transfection reagent. 24 hours post-transfection, the cells were replated into white 96-well plate (Corning #3600) in the presence or absence of fluorescent tracer (which binds specifically to NanoLuc-XIAP). The cells were then treated with IAP antagonists for 30 minutes before the addition of substrate for measurement. Using NanoLuc-XIAP as donor protein and fluorescent tracer as acceptor, dual-filtered luminescence was measured via Tecan Infinite®200 Pro (donor at 460 nm and acceptor at 618 nm) and BRET ratio (values at 618 nm / values at 460 nm) was determined. BRET response curve was plotted using GraphPad Prism software after general normalization by subtracting no tracer BRET values.

**Pharmacokinetics analysis of XIAP-specific antagonist A4**

The testing dose of A4 10 mg/kg was injected intraperitoneally into the orthotopic neuroblastoma PDXs. 150-200 μL of blood was taken from mice via cardiac puncture and tumors were harvested over the following time courses of 0, 0.5, 1, 2, 4, 24 and 48 hours post-dosing (n=3/time point). The concentrations of A4 in mouse plasma and tumor samples were determined and validated by a highly sensitive liquid chromatography tandem mass spectrometry (LC–MS/MS) method with multiple reaction monitoring (MRM) mode. The LC-MS/MS system consisted of Agilent 1290 ultra high-performance liquid chromatography (UHPLC) connected in tandem to Sciex QTRAP 5500 mass spectrometer system. Chromatographic separation was optimized using a liquid–liquid extraction and a reversed phase separation on a Kinetex F5 column (100 mm x 2.1 mm, 2.6 μm) with isocratic elution. Ethyl indole-2-carboxylate was used as the internal standard (IS). MRM transitions 441.2/189.0 (A4) and 190.1/114.1 (IS) were monitored with a dwell time of 300 msec and analyst 1.6.2 software (Sciex) was used to quantify the peaks with 1/x2 weighted linear regression.

**Statistical analysis**

Statistical analysis was performed using GraphPad Prism software version 5.0 (La Jolla, CA). Data were expressed as the mean ± standard deviation (S.D) with S.D. represented by the vertical error bars in figures. Statistical analysis was performed using two-tailed student t-test with a p-value <0.05 considered statistically significant. For orthotopic mice model, log-rank (Mantel-cox test) derived from Kaplan-Meier survival plots was used for statistical comparisons between groups.

**Key Resources Table**

| REAGENT or RESOURCE | SOURCE | IDENTIFIER |
| --- | --- | --- |
| Antibodies | | |
| Mouse monoclonal β-actin (C4) | Santa Cruz | Cat# sc-47778; RRID: AB_2714189 |
| Rabbit monoclonal PARP | Cell Signaling Technology | Cat# 9542, RRID: AB_2160739 |
| Rabbit monoclonal N-Myc (D1V2A) | Cell Signaling Technology | Cat# 84406, RRID: AB_2800038 |
| Rabbit monoclonal XIAP (D2Z8W) | Cell Signaling Technology | Cat# 14334, RRID: AB_2784533 |
| Rabbit monoclonal cleaved caspase-3 (Asp175) (5A1E) | Cell Signaling Technology | Cat# 9654, RRID: AB_10694088 |
| Rabbit monoclonal c-IAP1 (D5G9) | Cell Signaling Technology | Cat# 7065, RRID: AB_10890862 |
| Goat anti-mouse IgG, HRP-linked Antibody | Cell Signaling Technology | Cat# 7076; RRID: AB_330924 |
| Goat anti-rabbit IgG, HRP-linked Antibody | Cell Signaling Technology | Cat# 7074; RRID: AB_2099233 |
| XIAP (A-7) antibody | Santa Cruz | Cat# sc-55550; RRID: AB_ 831459 |
| Bacterial and virus strains | | |
| 3^rd^ generation lentiviral packaging plasmids | Thermo Fisher Scientific | Cat# K4975-00 |
| Biological samples |  |  |
| *MYCN*-amplified neuroblastoma PDX model | This study | N/A |
| Neuroblastoma patients’ samples | This study | N/A |
| Chemicals, peptides, and recombinant proteins | | |
| BV6 | A gift from Genentech | N/A |
| CUDC-427 | A gift from Curis | N/A |
| LCL161 | A gift from Novartis | N/A |
| Debio 1143 | A gift from Debiopharm | N/A |
| A4 | A gift Prof. Sarit Larisch | N/A |
| B3 | A gift Prof. Sarit Larisch | N/A |
| MG132 | Selleck Chemicals | Cat# S2619 |
| Vincristine sulfate (Vincristine) | Korea United Pharm., Inc | NDC 61703-309-06 |
| Topotecan hydrochloride (Topotecan) | Accord Healthcare | NDC 16729‐151‐31 |
| Lipofectamine 2000 Transfection Reagent | Thermo Fisher Scientific | Cat# 11668019 |
| FuGENE® HD transfection reagent | Promega | Cat# E2311 |
| Alt-R® S.p. Cas9 Nuclease V3 | Integrated DNA Technologies | Cat# 1081058 |
| Critical commercial assays | | |
| RealTime-Glo™ MT Cell Viability Assay | Promega | Cat# G9713 |
| Caspase-Glo® 3/7 Assay | Promega | Cat# G8091 |
| Nano-Glo® HiBiT lytic detection system | Promega | Cat# N3030 |
| Nano-Glo® Vivazine™ live cell substrate | Promega | Cat# N2581 |
| NanoBRET™ Target Engagement In-Cell IAP Assay | Promega | Cat# CS1810C431 |
| NanoBRET™ Ubiquitination Starter Kit | Promega | Cat# ND2690 |
| Dead Cell Apoptosis Kit with Annexin V FITC and PI | Thermo Fisher Scientific | Cat# V13242 |
| Experimental models: Cell lines | | |
| SK-N-SH | ATCC | Cat# HTB-11; RRID: CVCL_0531 |
| SK-N-AS | ATCC | Cat# CRL-2137; RRID: CVCL_1700 |
| NB1 | Japanese Collection of Research Bioresources | Cat# JCRB0621  RRID: CVCL_1440 |
| CHP212 | ATCC | Cat# CRL-2273; RRID: CVCL_1125 |
| NLF | Kerafast | Cat# ECP008  RRID: CVCL_E217 |
| KELLY | Sigma-Aldrich | Cat# 92110411; RRID: CVCL_2092 |
| BE(2)-C | ATCC | Cat# CRL-2268; RRID: CVCL_0529 |
| IMR-32 | ATCC | Cat# CCL-127; RRID: CVCL_0346 |
| HS5 | ATCC | Cat# CRL-11882; RRID: CVCL_3720 |
| THLE3 | ATCC | Cat# CRL-11233; RRID: CVCL_3804 |
| Patient-derived neuroblastoma cells (NBL27-0218A, NBL16-0118, NBL01-1116, NBL07-0317, NBL01-1116) | Dr. Amos Loh Hong Pheng, via VIVA-KKH Paediatric Brain and Solid Tumour Programme | N/A |
| Experimental models: Organisms/strains | | |
| NOD.Cg-Prkdc^scid^/JInv | InVivos Pte Ltd (The Jackson Laboratory) | JAX: 001303 |
| Oligonucleotides | | |
| Multiplex HiBiT-XIAP Primer (5’ to 3’)  F: TGTTCCTTGTGGACATCTAGTC  R1: AAGTCAGTTCACATCACACATTC  R2: GCTAATCTTCTTGAACAGCCG | This study | N/A |
| C-terminal XIAP crRNA (Guide RNA1): TCTTAATCTAACTCTATAGT | This study | N/A |
| C-terminal Donor DNA template (ssODN):  GTTGACAAGTGTCCCATGTGCTACACAGTCATTACTTTCAAGCAAAAAATTTTTATGTCTGTGAGCGGCTGGCGGCTGTTCAAGAAGATTAGCTAATCTAACTCTATAGTAGGCATGTTATGTTGTTCTTATTACCCTGATTGAATGTGTGATGTG | This study | N/A |
| Alt-R® CRISPR-Cas9 tracrRNA | Integrated DNA Technologies | Cat# 1072532 |
| Recombinant DNA | | |
| XIAP Plasmid | Addgene | RRID: Addgene_11833 |
| XIAP shRNAs | Sigma-Aldrich MISSION® | TRCN0000231578; TRCN0000231579 |
| Non-Targeting shRNA Control | Sigma-Aldrich MISSION® | SHC202 |
| Software and algorithms | | |
| GraphPad Prism 5 | GraphPad Software | www.graphpad.com |
| ImageJ | NIH | https://imagej.nih.gov/ij/ |
