# Supplementary material for: Targeted Degradation of XIAP is Sufficient and Specific to Induce Apoptosis in MYCN-overexpressing High-risk Neuroblastoma
Source: Cancer Res Commun. 2023 Nov 22;3(11):2386–99. doi: 10.1158/2767-9764.CRC-23-0082 (PMC10681007; doi:10.1158/2767-9764.CRC-23-0082)
Supplement: Supplementary Data - raw Western blot images [file crc-23-0082-s05.pdf]

WB Raw Images

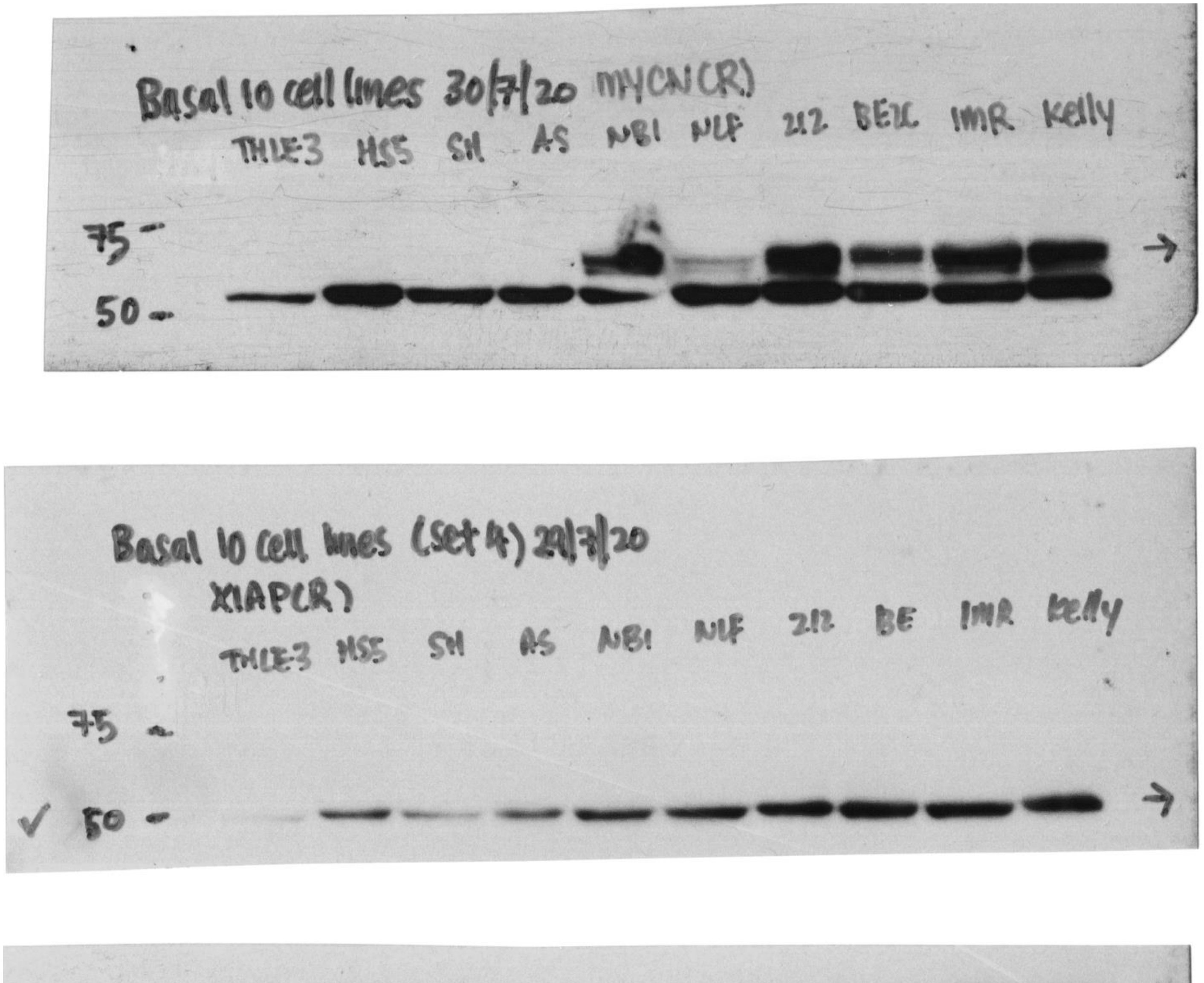

Fig. 1A

WB Raw Images

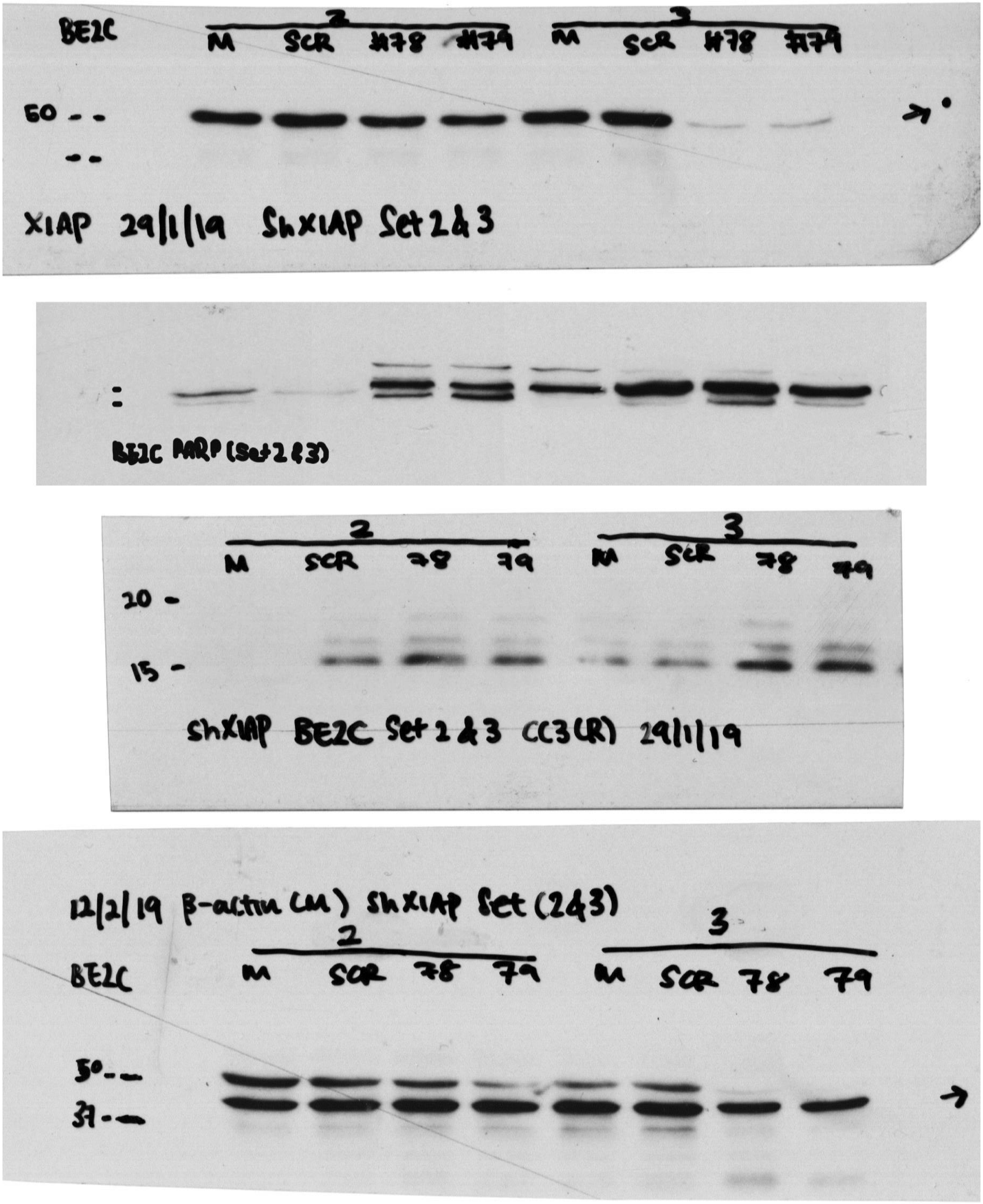

Fig. 1C – BE(2)-C

WB Raw Images

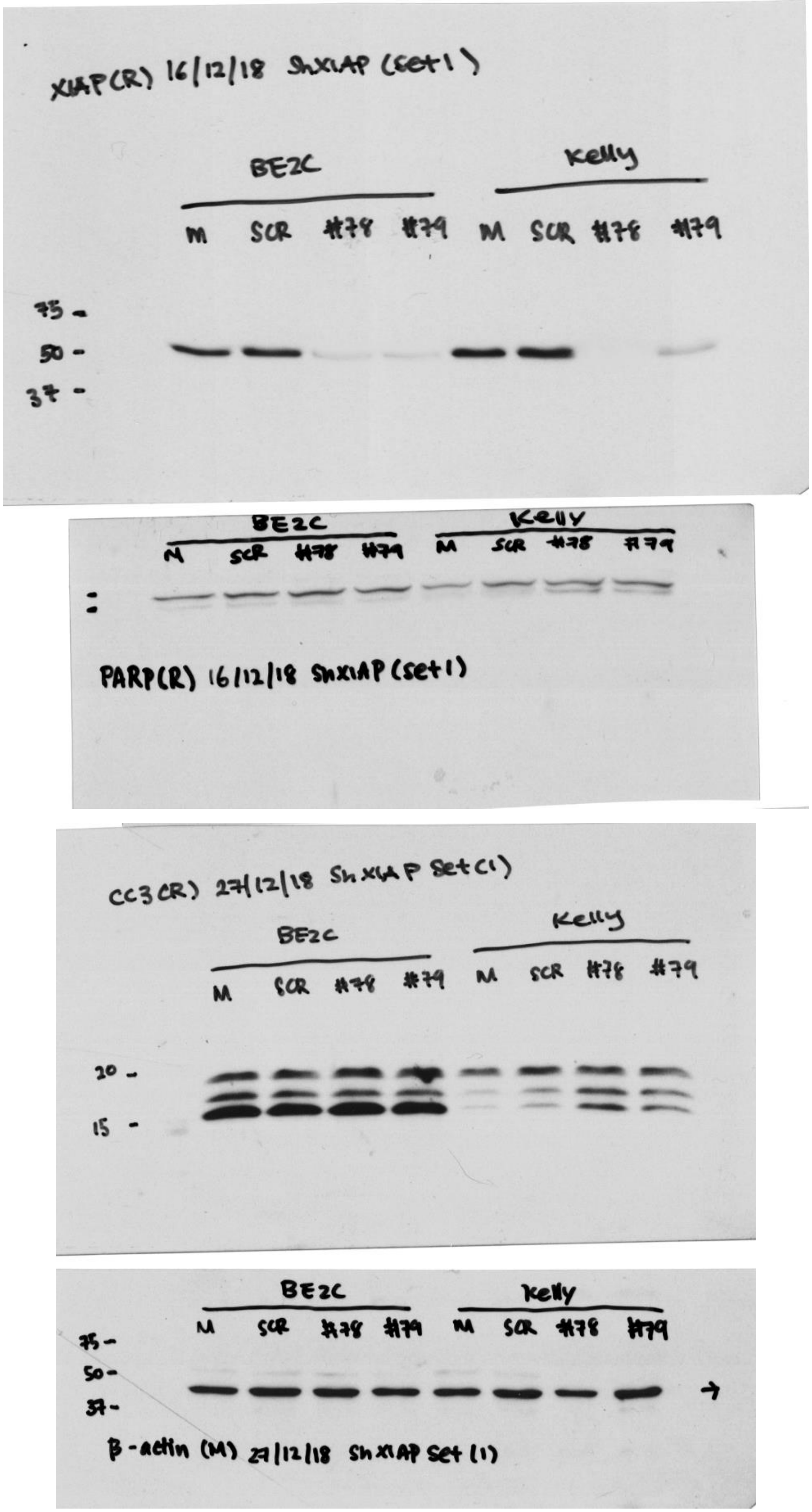

Fig. 1C – KELLY

WB Raw Images

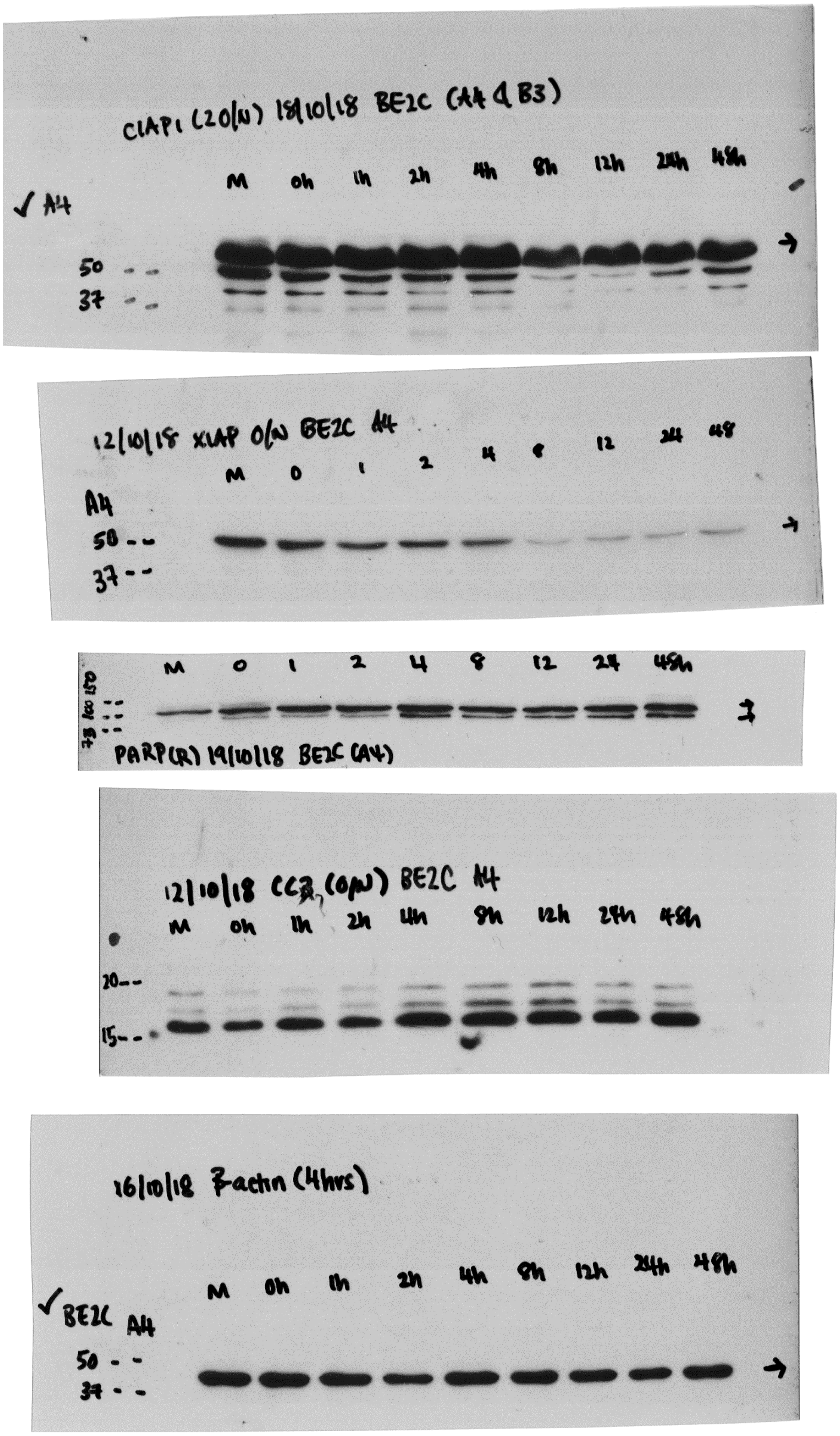

Fig. 2B – BE(2)-C (A4)

WB Raw Images

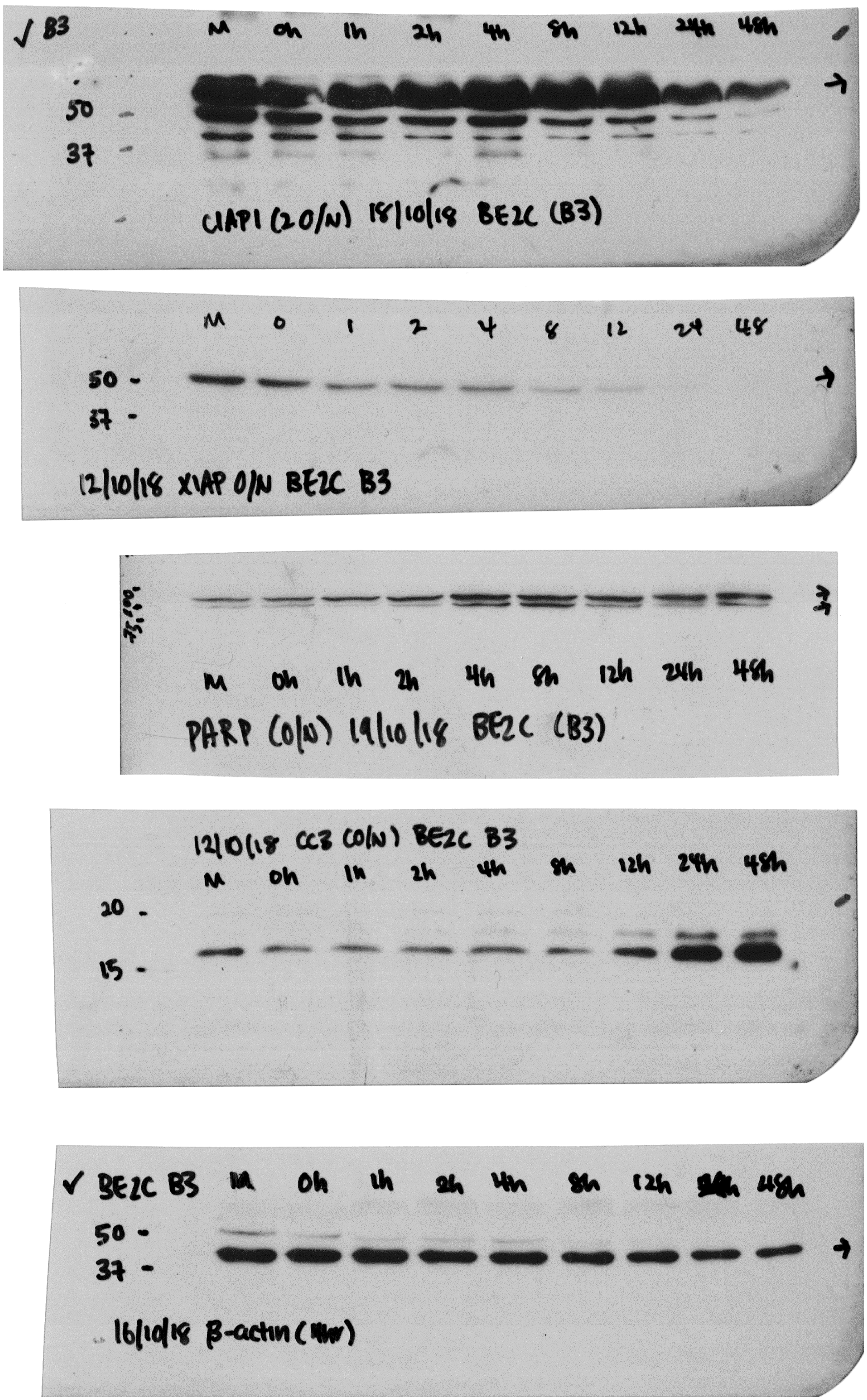

Fig. 2B – BE(2)-C (B3)

WB Raw Images

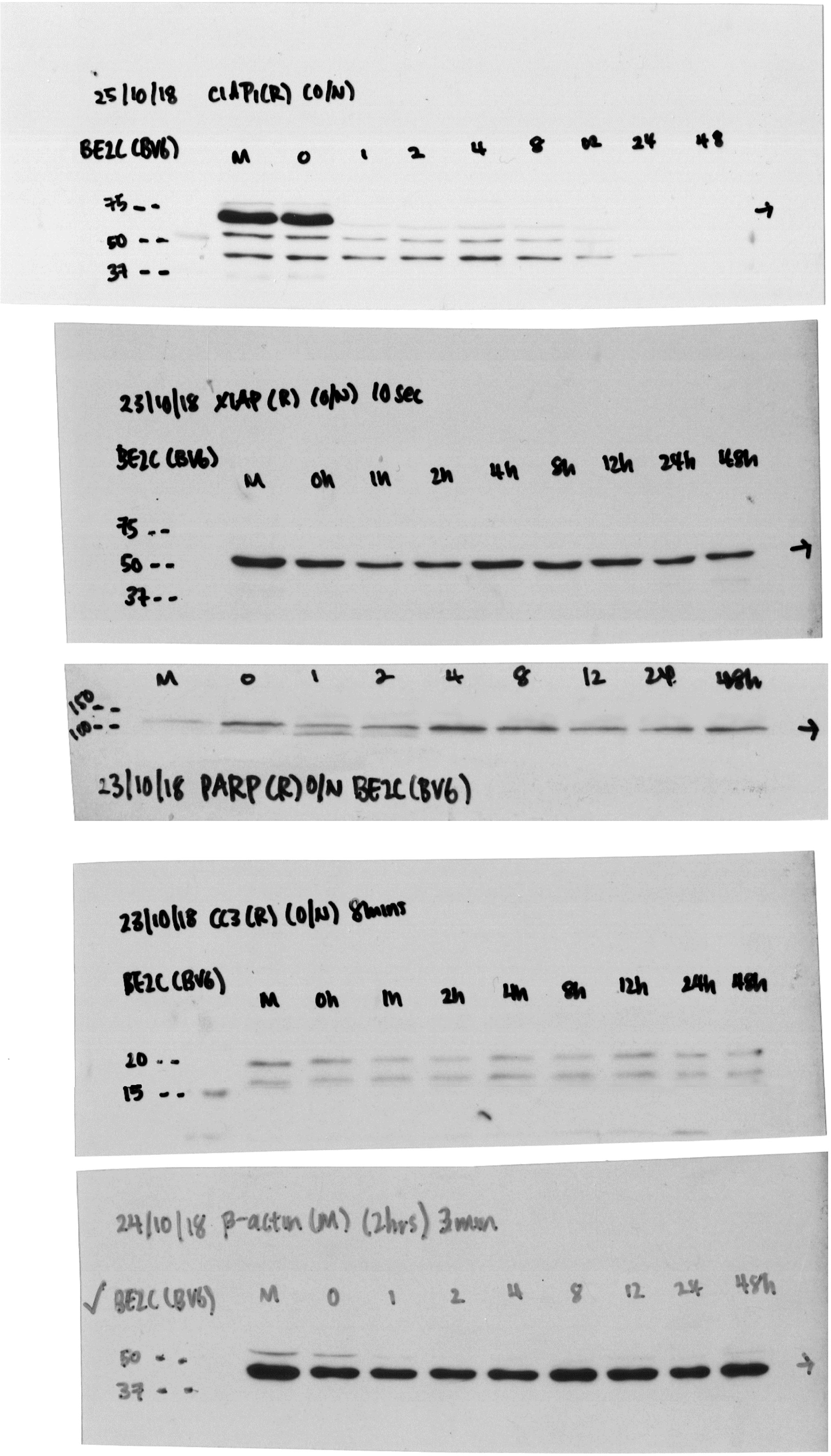

Fig. 2B – BE(2)-C (BV6)

WB Raw Images

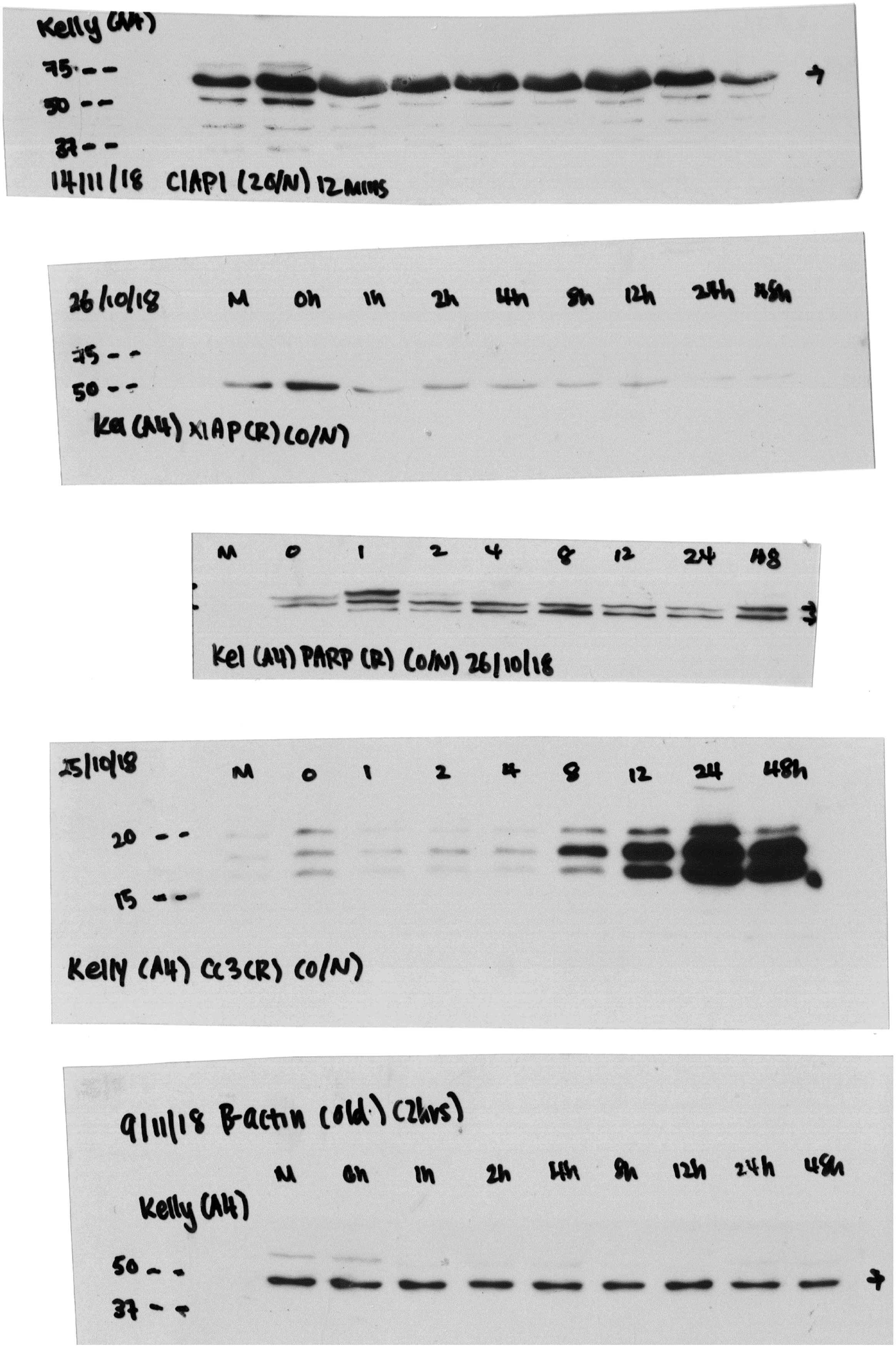

Fig. 2B – KELLY (A4)

WB Raw Images

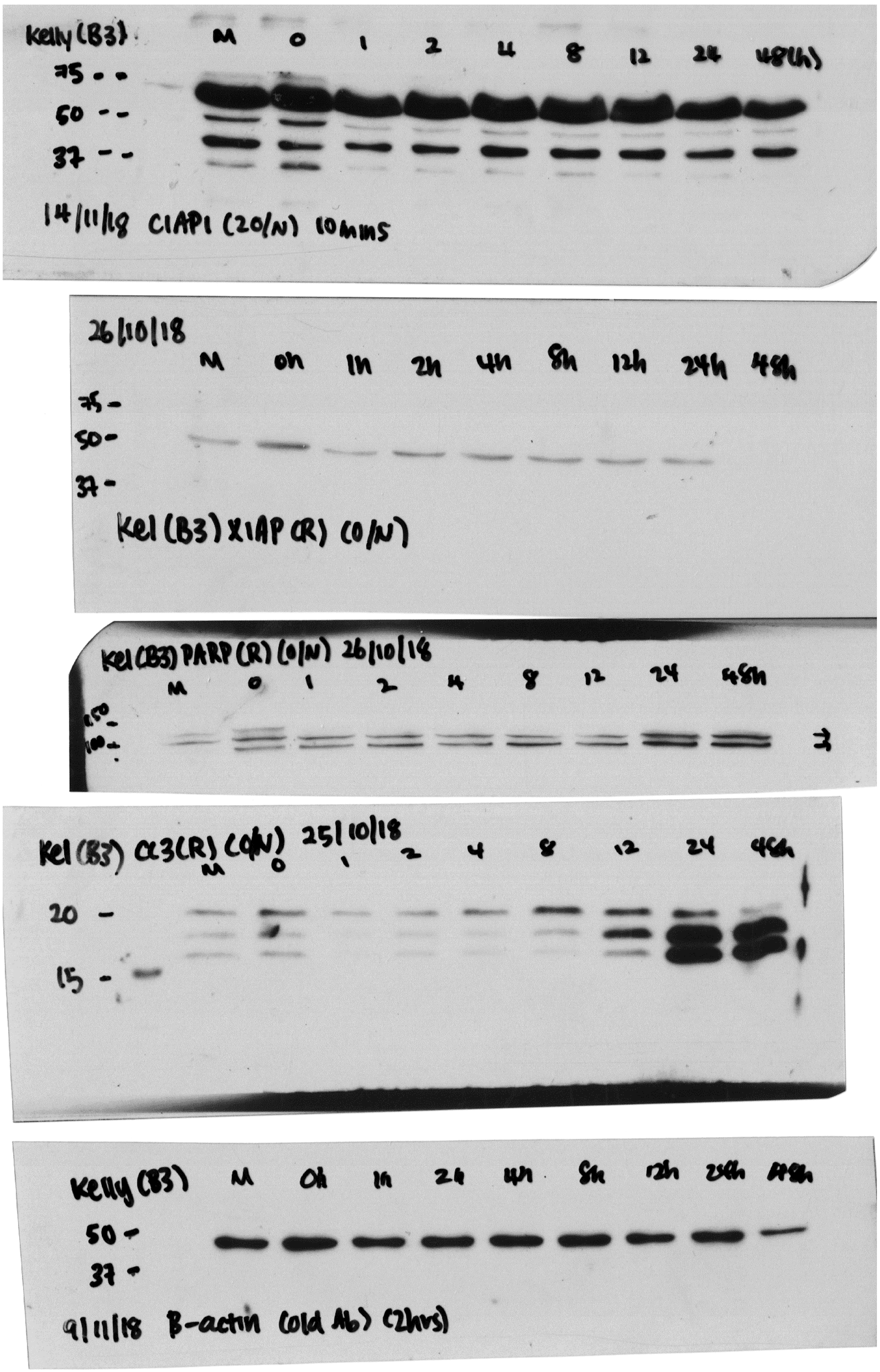

Fig. 2B – KELLY (B3)

WB Raw Images

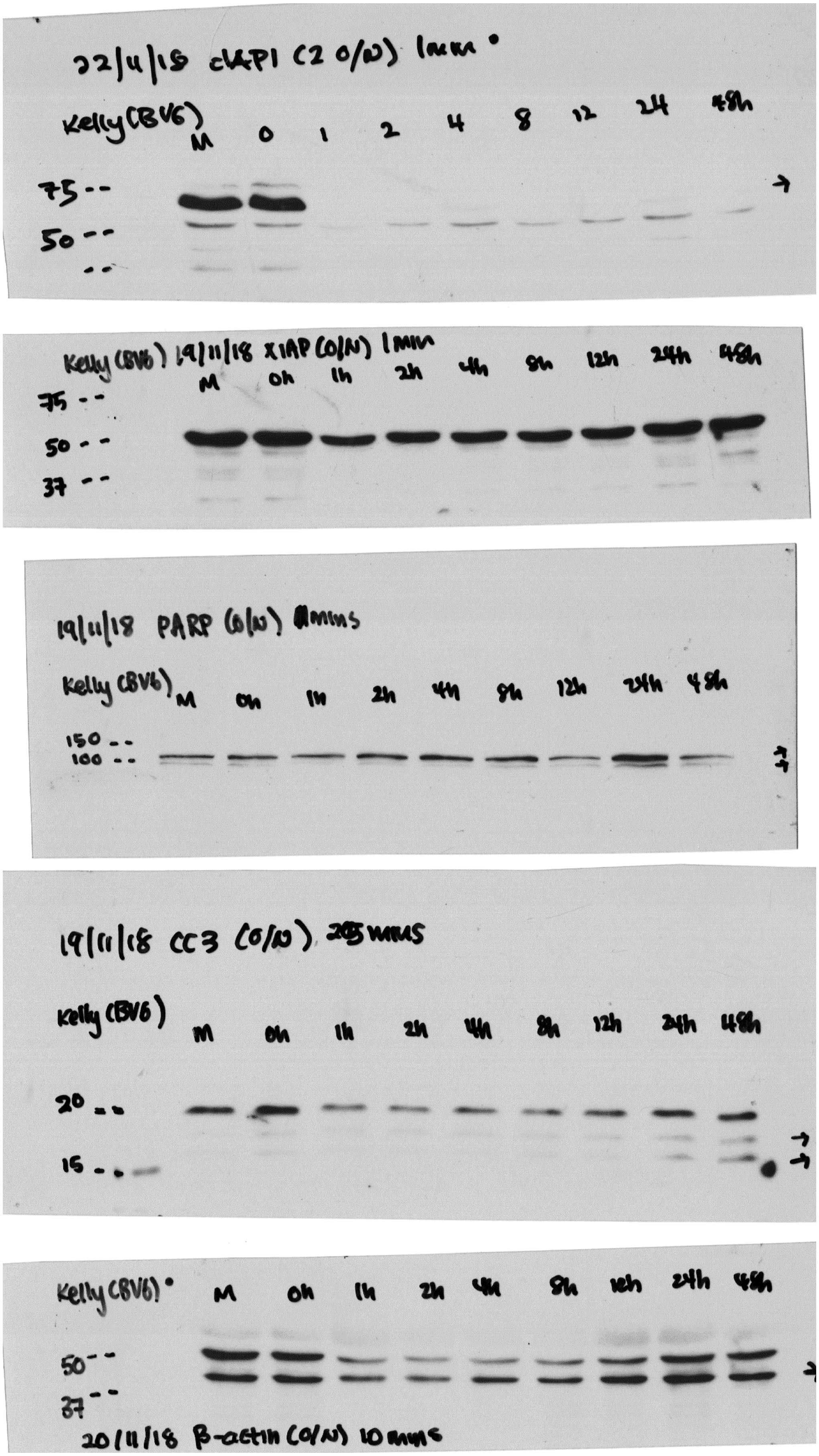

Fig. 2B – KELLY (BV6)

WB Raw Images

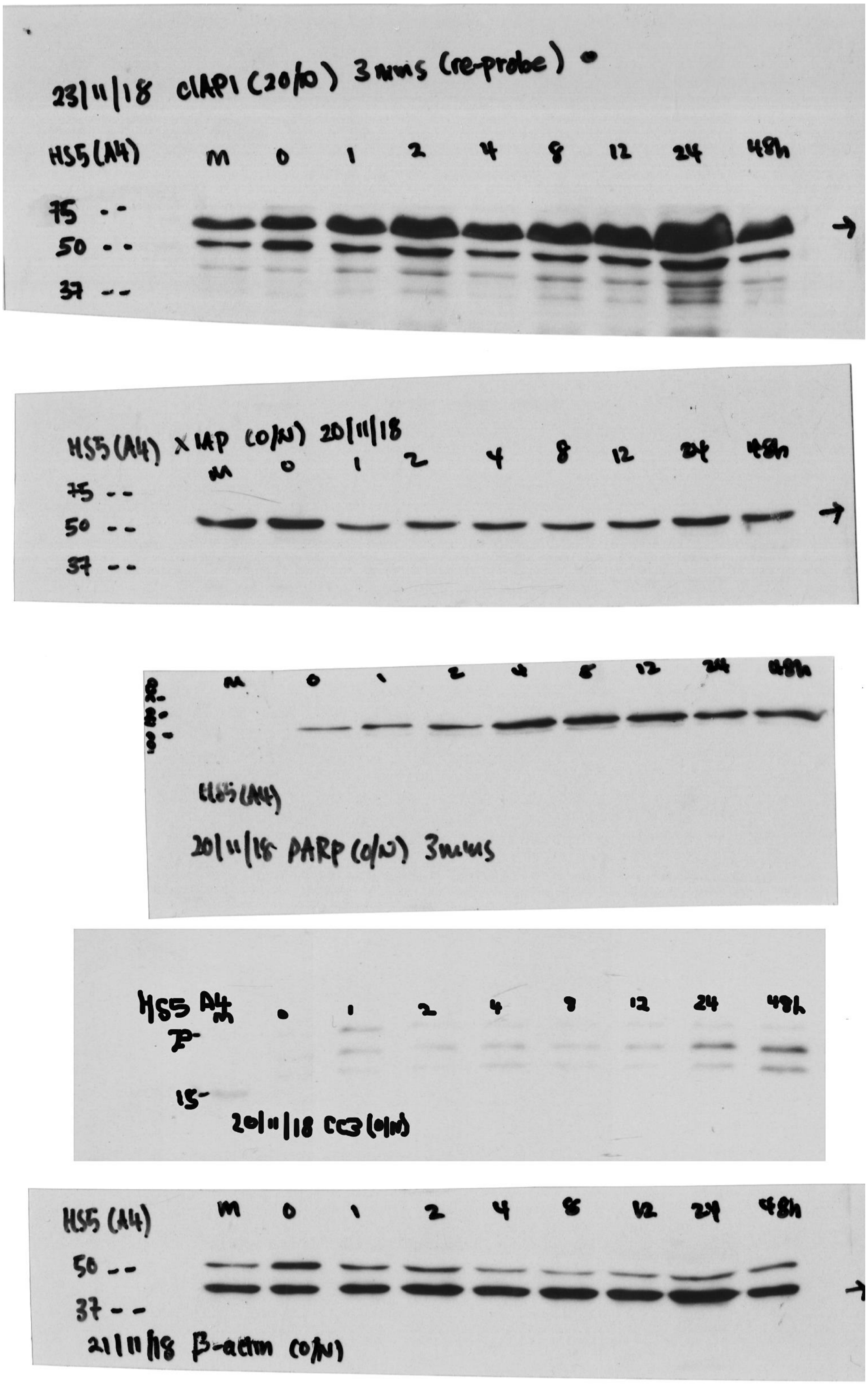

Fig. 2B – HS5 (A4)

WB Raw Images

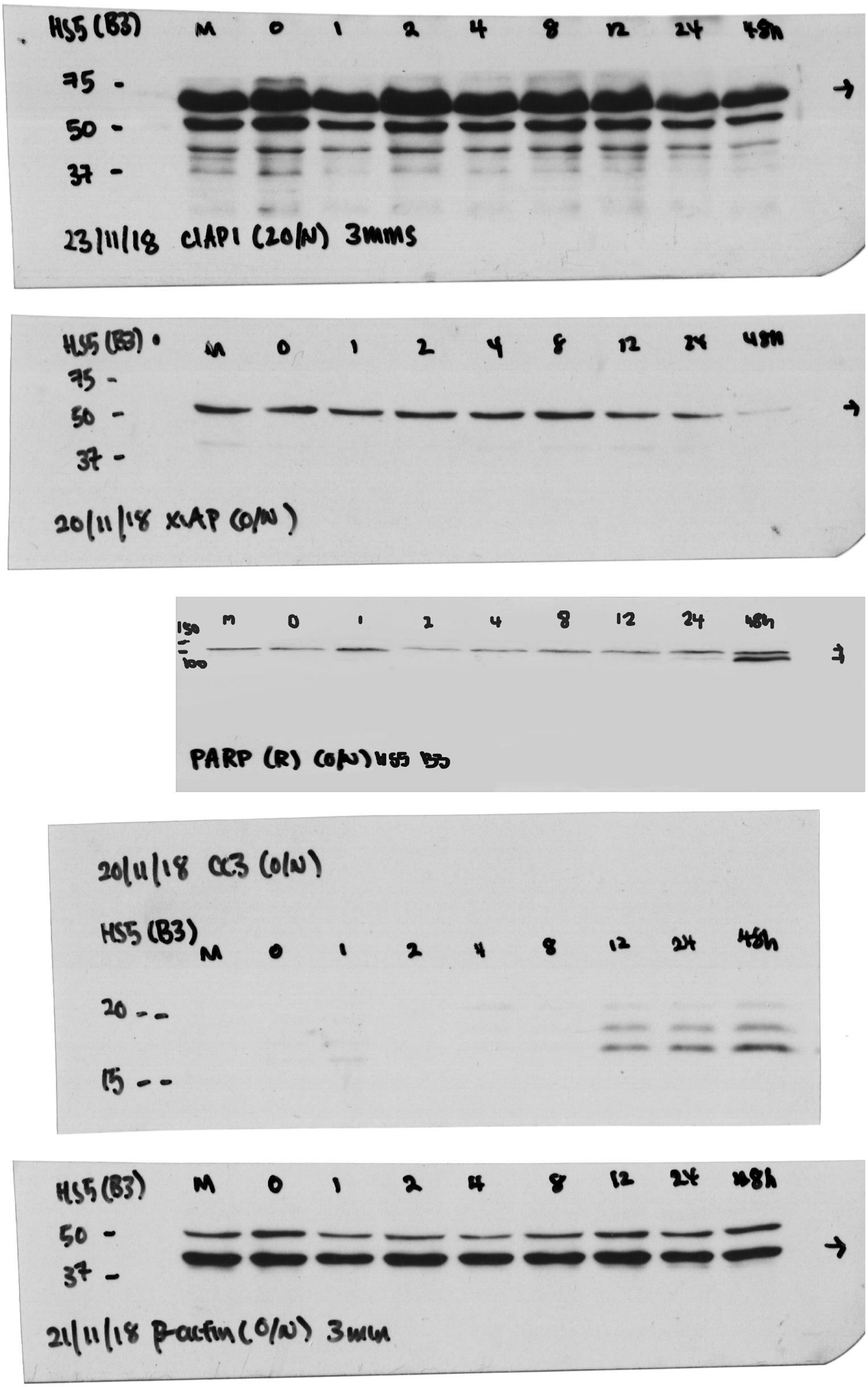

Fig. 2B – HS5 (B3)

WB Raw Images

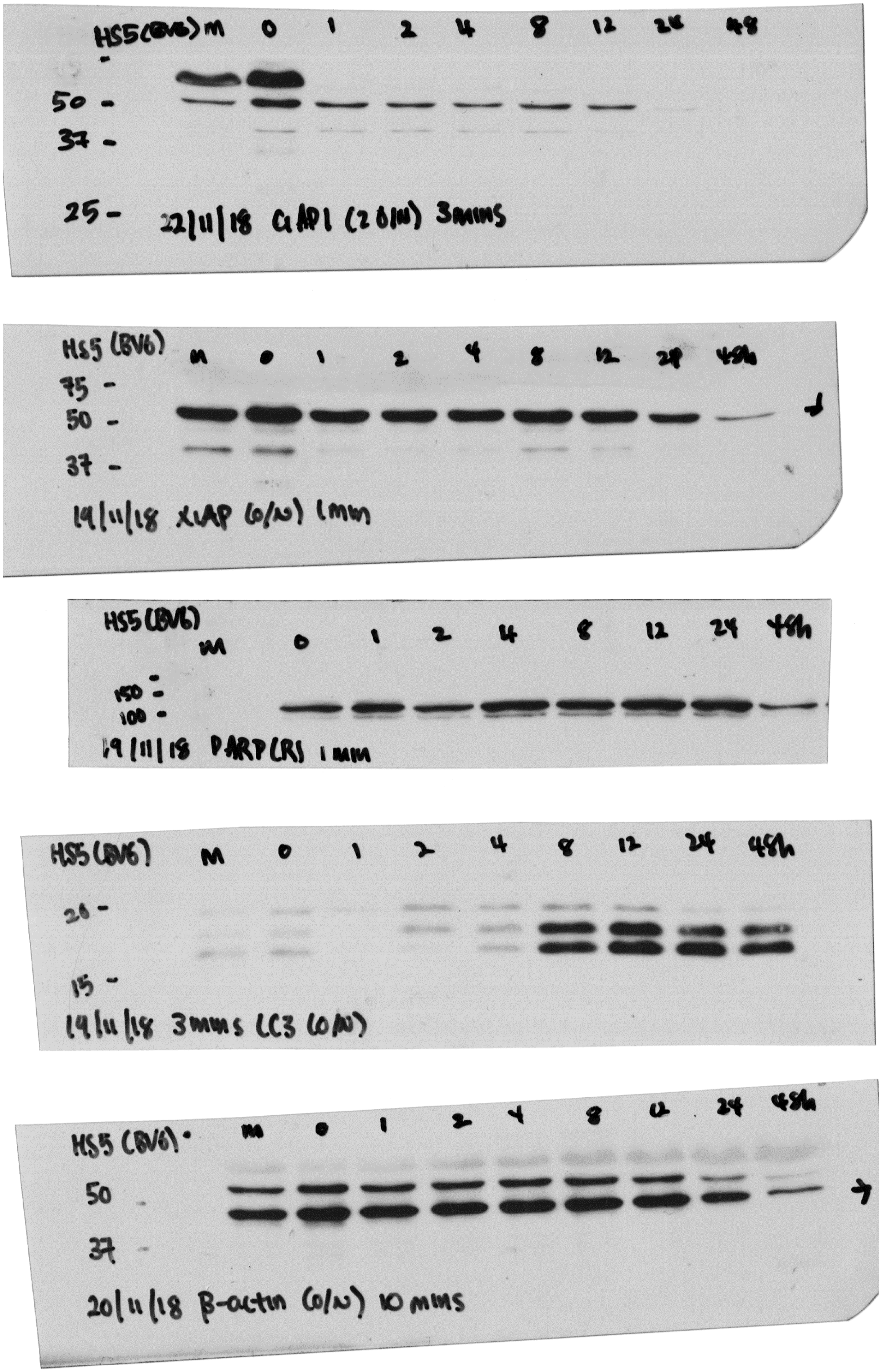

Fig. 2B – HS5 (BV6)

WB Raw Images

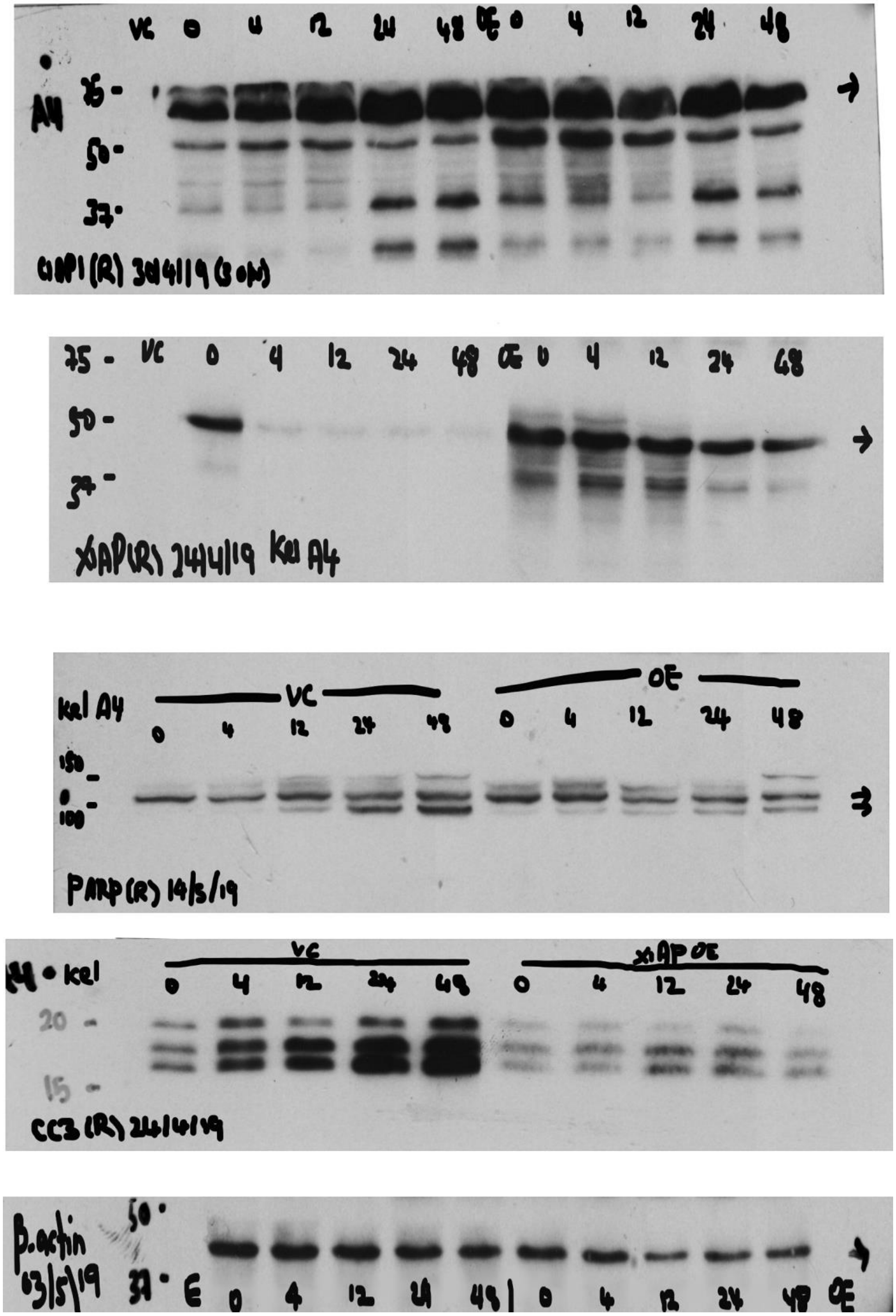

Fig. 2E – KELLY (A4)

WB Raw Images

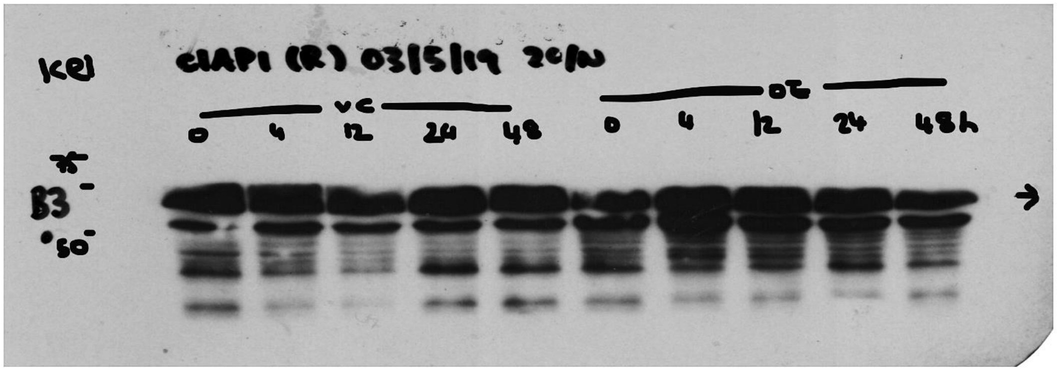

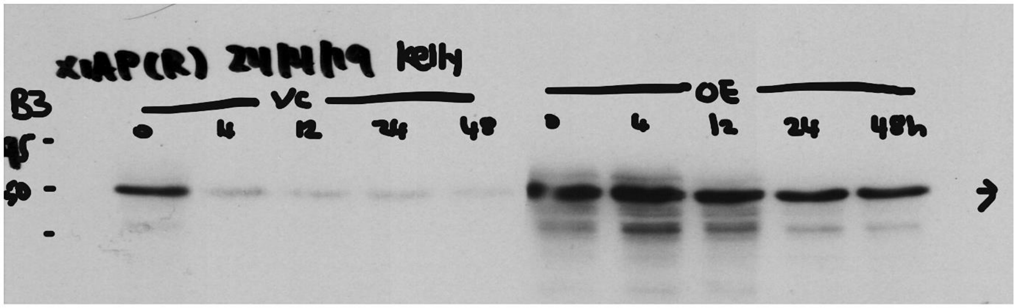

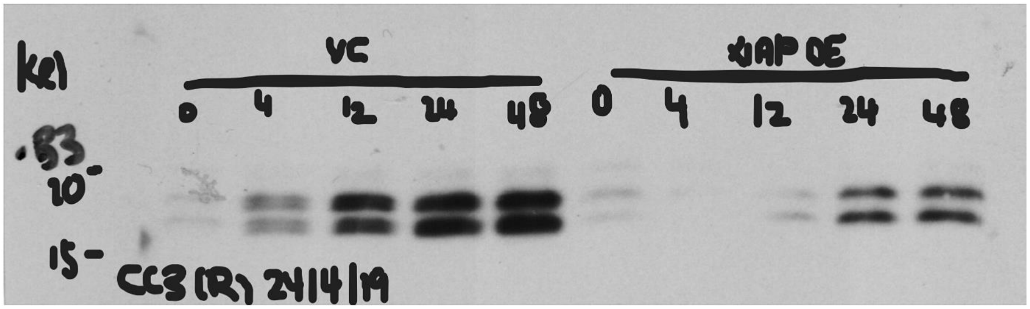

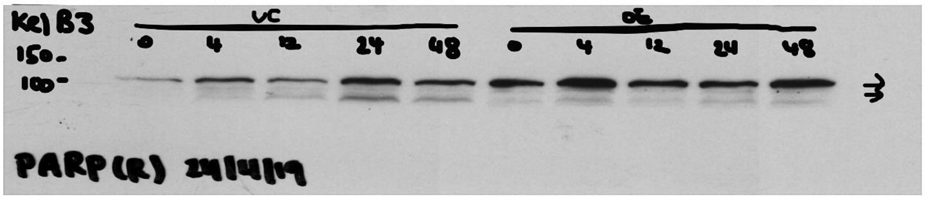

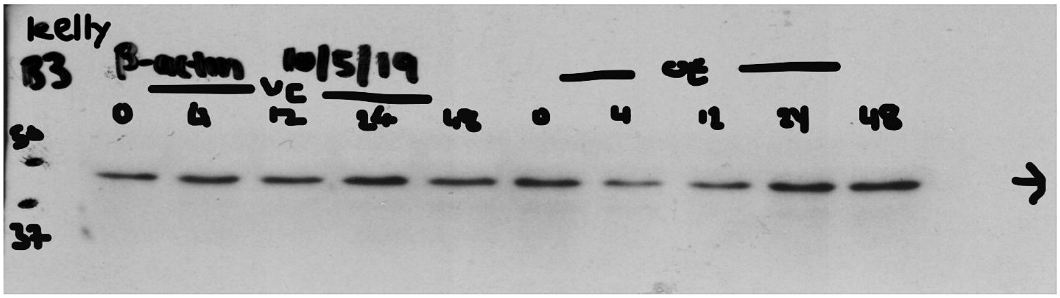

Fig. 2E – KELLY (B3)

WB Raw Images

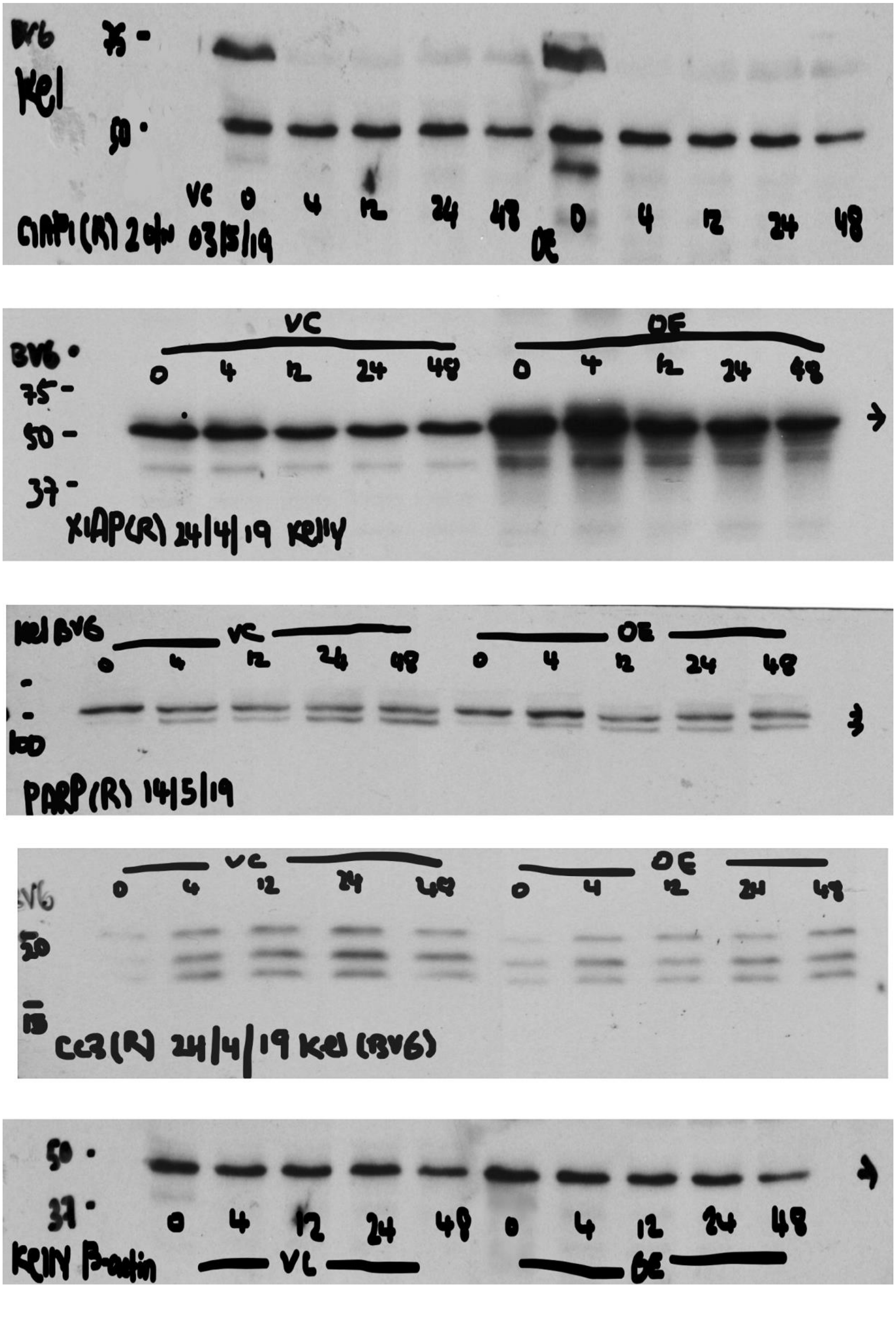

Fig. 2E – KELLY (BV6)

WB Raw Images

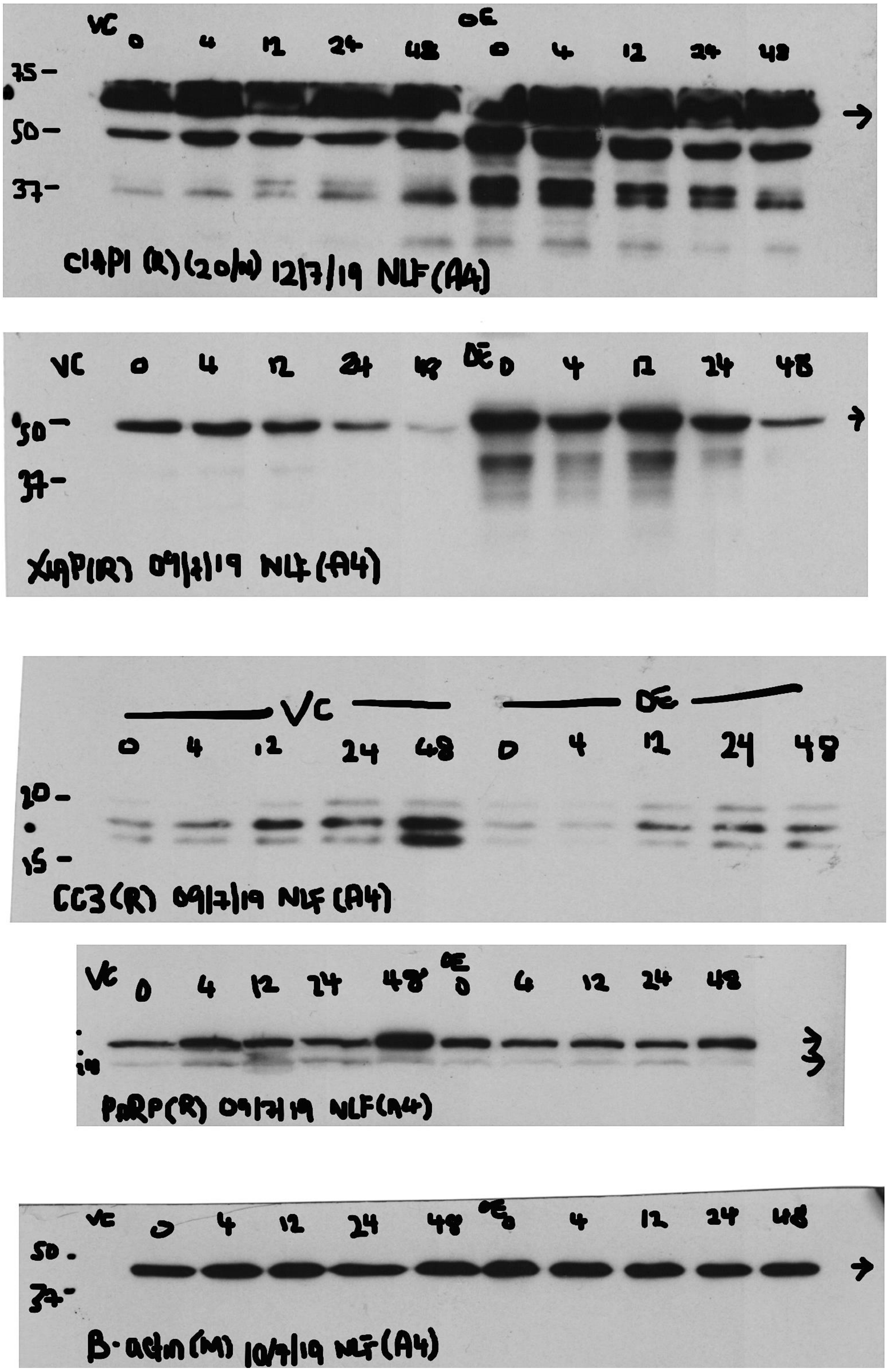

Fig. 2E – NLF (A4)

WB Raw Images

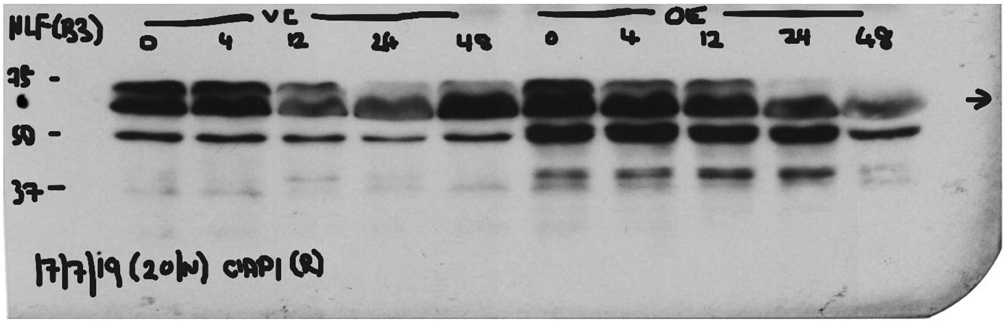

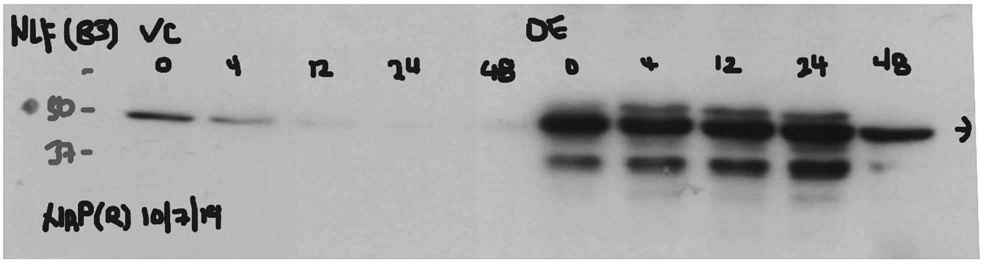

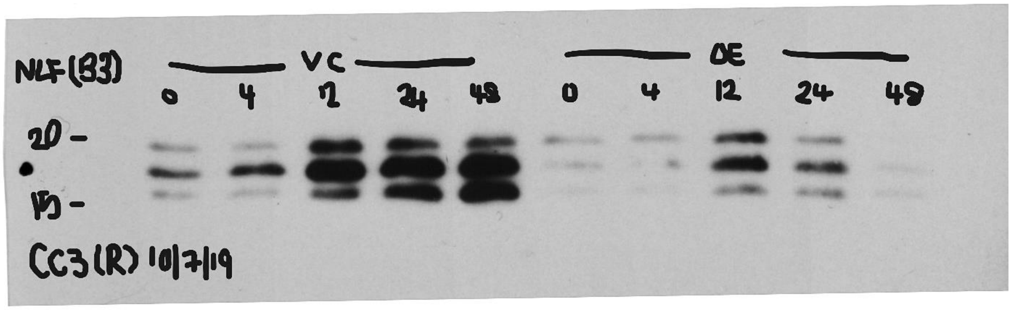

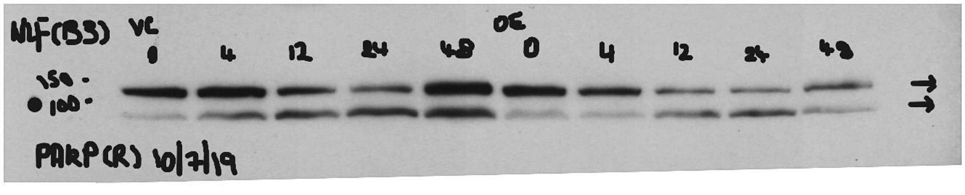

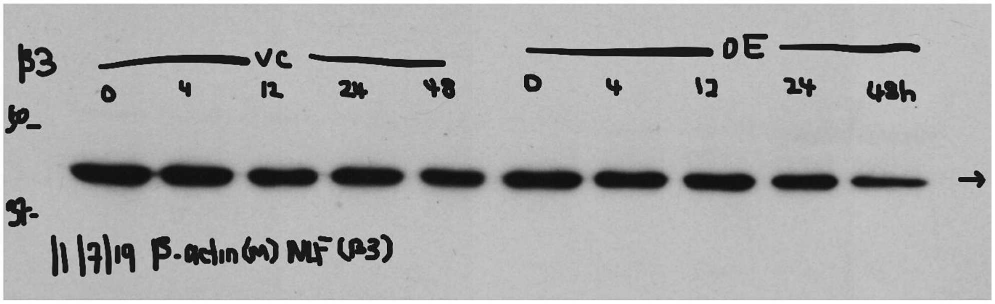

Fig. 2E – NLF (B3)

WB Raw Images

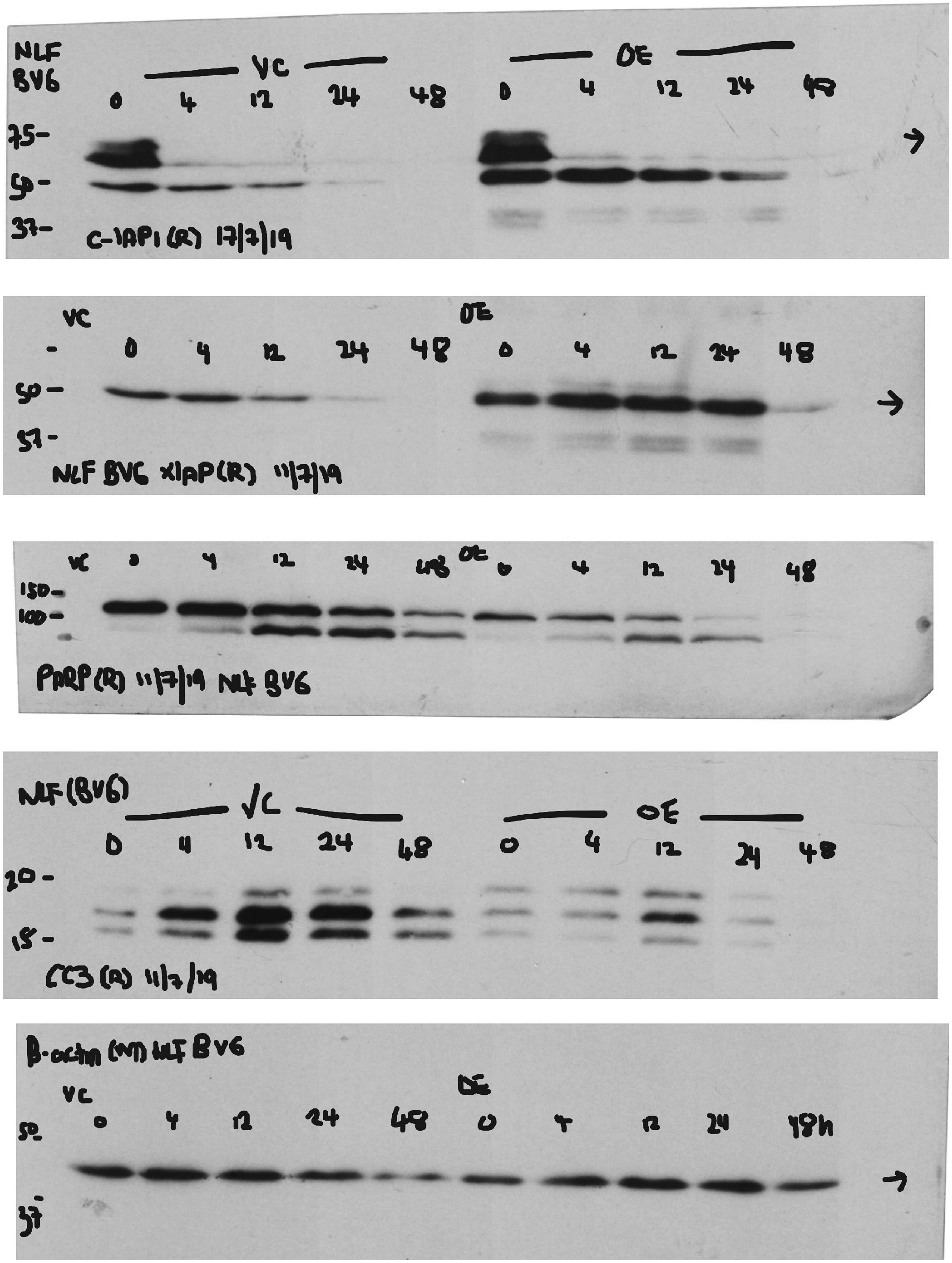

Fig. 2E – NLF (BV6)

WB Raw Images

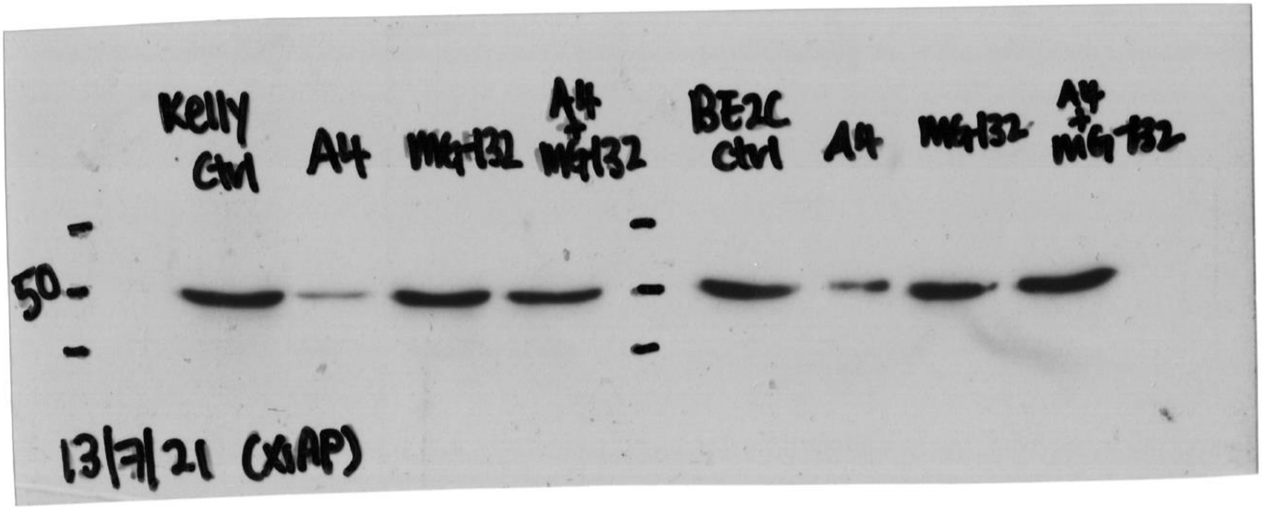

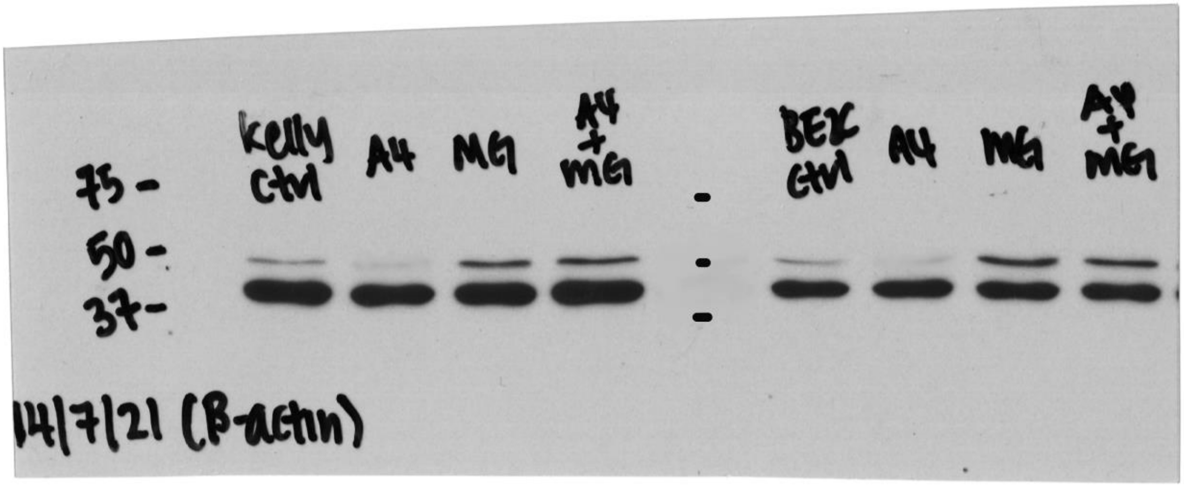

Fig. 3D

WB Raw Images

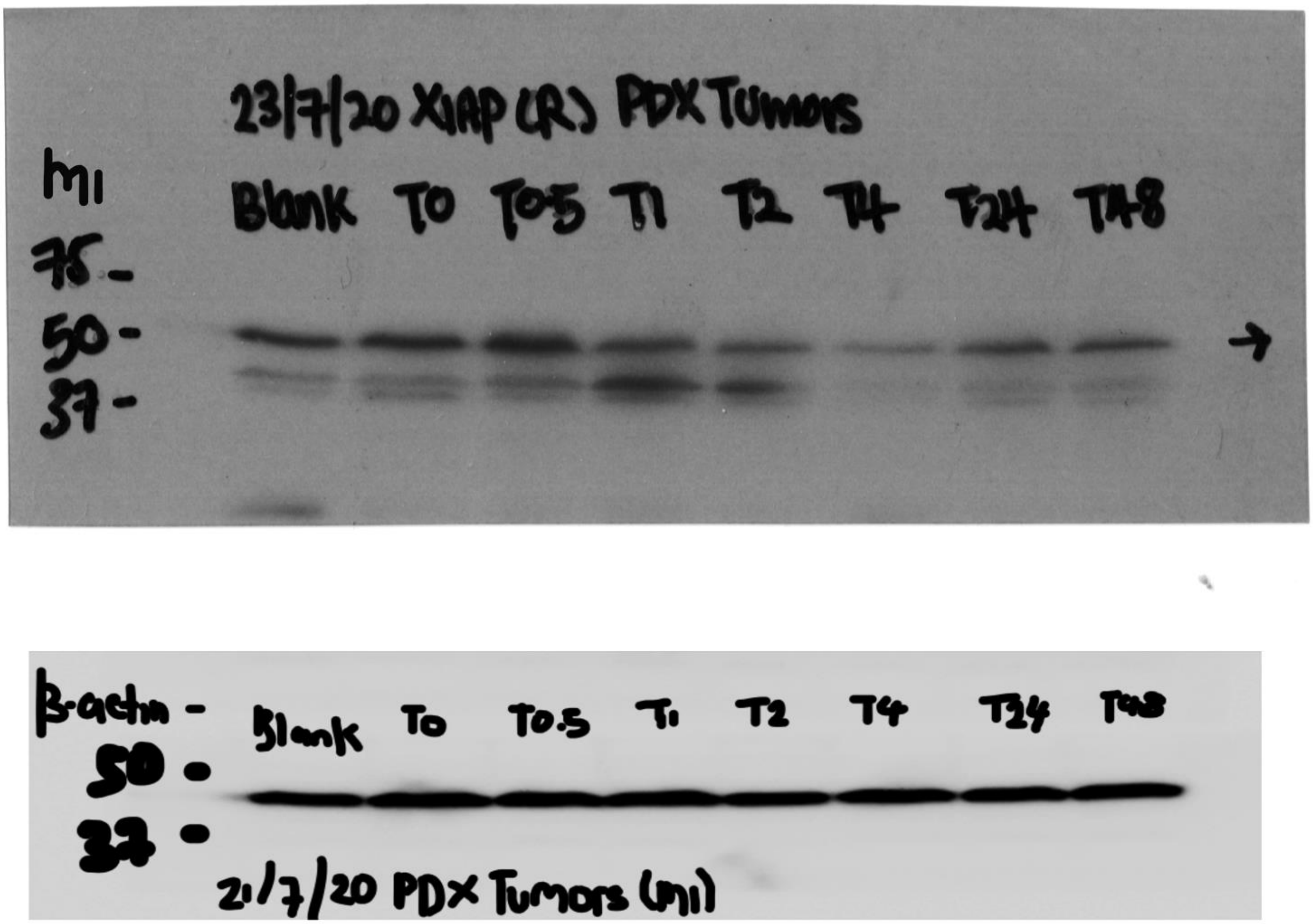

Fig. 4A
