# Supplementary material for: Targeted Degradation of XIAP is Sufficient and Specific to Induce Apoptosis in MYCN-overexpressing High-risk Neuroblastoma
Source: Cancer Res Commun. 2023 Nov 22;3(11):2386–99. doi: 10.1158/2767-9764.CRC-23-0082 (PMC10681007; doi:10.1158/2767-9764.CRC-23-0082)
Supplement: Table S1 — Supplementary Table S1, related to Figure 1. Table of densitometry values calculated from western blot to generate scatter plot in Figure 1A. [file crc-23-0082-s06.docx]

**Table S1, related to Figure 1. Table of densitometry values calculated from western blot to generate scatter plot in Figure 1A.**

| **Raw Densitometry Values** | | | | | | | | | | | |
| --- | --- | --- | --- | --- | --- | --- | --- | --- | --- | --- | --- |
|  | **THLE3** | **HS5** | **SK-N-SH** | **SK-N-AS** | **NB1** | **NLF** | **CHP212** | **BE(2)-C** | **IMR-32** | **KELLY** |  |
| **MYCN** |  |  |  |  | 10516.36 | 3920.84 | 15373.43 | 11946.13 | 18344.62 | 17140.60 |  |
| **XIAP** | 234.92 | 1972.77 | 915.70 | 1858.01 | 3625.77 | 4449.84 | 5289.77 | 6482.43 | 6306.60 | 6055.67 |  |
| **β-actin** | 3982.65 | 5849.72 | 6033.13 | 4876.06 | 5335.77 | 7178.01 | 6875.48 | 5825.36 | 9481.43 | 6437.60 |  |
|  | | | | | | | | | | | |
| **Densitometry ratio for scatter plot** | | | | | | | | | | | |
| **MYCN/ β-actin** | 0.00 | 0.00 | 0.00 | 0.00 | 1.97 | 0.55 | 2.24 | 2.05 | 1.93 | 2.66 |  |
| **XIAP/ β-actin** | 0.06 | 0.34 | 0.15 | 0.38 | 0.68 | 0.62 | 0.77 | 1.11 | 0.67 | 0.94 |  |
