# Supplementary material for: Targeted Degradation of XIAP is Sufficient and Specific to Induce Apoptosis in MYCN-overexpressing High-risk Neuroblastoma
Source: Cancer Res Commun. 2023 Nov 22;3(11):2386–99. doi: 10.1158/2767-9764.CRC-23-0082 (PMC10681007; doi:10.1158/2767-9764.CRC-23-0082)
Supplement: Table S2 — Supplementary Table S2, related to Figure 2. Table of IC50 (μM) indicating the effect of individual IAP antagonists on viability of neuroblastoma and normal tissue-derived cells measured every 24 hours up to 72 hours. [file crc-23-0082-s07.docx]

**Table S2, related to Figure 2. Table of IC_50_ (μM) indicating the effect of individual IAP antagonists on viability of neuroblastoma and normal tissue-derived cells measured every 24 hours up to 72 hours.**

| **24 hours**  **IAP Antagonists** | | | | | | |
| --- | --- | --- | --- | --- | --- | --- |
| **Cell lines** | **A4** | **B3** | **BV6** | **LCL161** | **CUDC-427** | **Debio 1143** |
| **BE(2)-C** | 5.9 | 7.6 | 21.2 | >60 | 64.9 | >100 |
| **IMR-32** | 11.9 | 9.3 | 7.4 | >60 | 59.0 | >100 |
| **KELLY** | 4.8 | 10.5 | 13.5 | 48.2 | 32.5 | 91 |
| **NB1** | 9.2 | 9.1 | 7 | >60 | 42.0 | >100 |
| **NLF** | 8.3 | 2.9 | 7.4 | 32.8 | 45.0 | >100 |
| **CHP212** | 11.2 | 6.5 | 10.4 | 42 | 57.6 | >100 |
| **SK-N-SH** | 15 | 5.5 | 12.4 | 55.4 | 54.1 | >100 |
| **SK-N-AS** | 3.5 | 6.5 | 4.6 | 34 | 33.2 | 68.5 |
| **NBL-07-0317** | 14.2 | 8.7 | 8.1 | 33.6 | 33.5 | >100 |
| **NBL01-1116** | 19.8 | 8.0 | 14.5 | 42 | 40.0 | >100 |
| **NBL16-0118** | 2.0 | 11.8 | 5.1 | 34 | 35.0 | >100 |
| **NBL27-0218A** | 5.9 | 30.8 | 5.9 | 39 | 32.8 | >100 |
| **THLE3** | 26.8 | 9.8 | 11.3 | >60 | 40.2 | >100 |
| **HS5** | 17.6 | 5.6 | 6.1 | 30 | 29.9 | >100 |
| **48 hours** | | | | | | |
| **BE(2)-C** | 4.5 | 6.7 | 16.8 | 42.5 | 60.9 | >100 |
| **IMR-32** | 7.9 | 5.8 | 5.8 | 30 | 46.5 | >100 |
| **KELLY** | 3.9 | 6.7 | 12.9 | 27.6 | 29.5 | 73.5 |
| **NB1** | 5.3 | 9.0 | 5.5 | 46.5 | 38.1 | >100 |
| **NLF** | 6.2 | 2.7 | 3.8 | 30.2 | 38.6 | 78.5 |
| **CHP212** | 7.7 | 5.7 | 9.9 | 33 | 50.1 | >100 |
| **SK-N-SH** | 12.9 | 5.3 | 10.5 | 44 | 40 | >100 |
| **SK-N-AS** | 5.2 | 6.3 | 3.2 | 22.5 | 26.9 | 50.5 |
| **NBL-07-0317** | 15.4 | 9.5 | 5.5 | 31.8 | 33 | 93 |
| **NBL01-1116** | 19.6 | 8.2 | 9.3 | 36.4 | 38 | >100 |
| **NBL16-0118** | 2.2 | 12 | 2.8 | 27.5 | 32 | >100 |
| **NBL27-0218A** | 7.0 | 26.6 | 3.6 | 32.5 | 30.4 | >100 |
| **THLE3** | 16 | 9.1 | 6.8 | 24 | 28.8 | 73 |
| **HS5** | 19.3 | 6.0 | 4.4 | 18 | 26.5 | 90 |
| **72 hours** | | | | | | |
| **BE(2)-C** | 3.7 | 7.0 | 16.5 | 32.5 | 55.6 | >100 |
| **IMR-32** | 6.5 | 4.8 | 4.3 | 19 | 40.6 | 64.5 |
| **KELLY** | 3.3 | 6.1 | 10.1 | 21.2 | 26 | >100 |
| **NB1** | 4.6 | 8.4 | 3.8 | 27.4 | 36.9 | 64.5 |
| **NLF** | 5.9 | 4.9 | 3.7 | 28.5 | 30.2 | >100 |
| **CHP212** | 7.5 | 5.1 | 9.3 | 31.2 | 50 | >100 |
| **SK-N-SH** | 11.6 | 4.9 | 9.7 | 33 | 33.5 | 35.5 |
| **SK-N-AS** | 4.7 | 5.1 | 2.6 | 15.7 | 22.3 | 85 |
| **NBL-07-0317** | 15.7 | 9.9 | 4.8 | 30 | 32 | >100 |
| **NBL01-1116** | 20.3 | 8.1 | 8.5 | 36 | 38 | >100 |
| **NBL16-0118** | 2.4 | 12.2 | 2.3 | 22.5 | 30 | >100 |
| **NBL27-0218A** | 8.5 | 21.1 | 2.6 | 28.5 | 28.4 | 50 |
| **THLE3** | 12.6 | 10.2 | 5.2 | 14.5 | 29.5 | 80 |
| **HS5** | 16.6 | 6.4 | 3.6 | 15.5 | 23.8 | >100 |
