# Supplementary material for: Targeted Degradation of XIAP is Sufficient and Specific to Induce Apoptosis in MYCN-overexpressing High-risk Neuroblastoma
Source: Cancer Res Commun. 2023 Nov 22;3(11):2386–99. doi: 10.1158/2767-9764.CRC-23-0082 (PMC10681007; doi:10.1158/2767-9764.CRC-23-0082)
Supplement: Figure S1 — Supplementary Figure S1, related to Figure 2. Importance of XIAP expression level in mediating sensitivity of neuroblastoma cells to A4, B3 and BV6. [file crc-23-0082-s08.pdf]

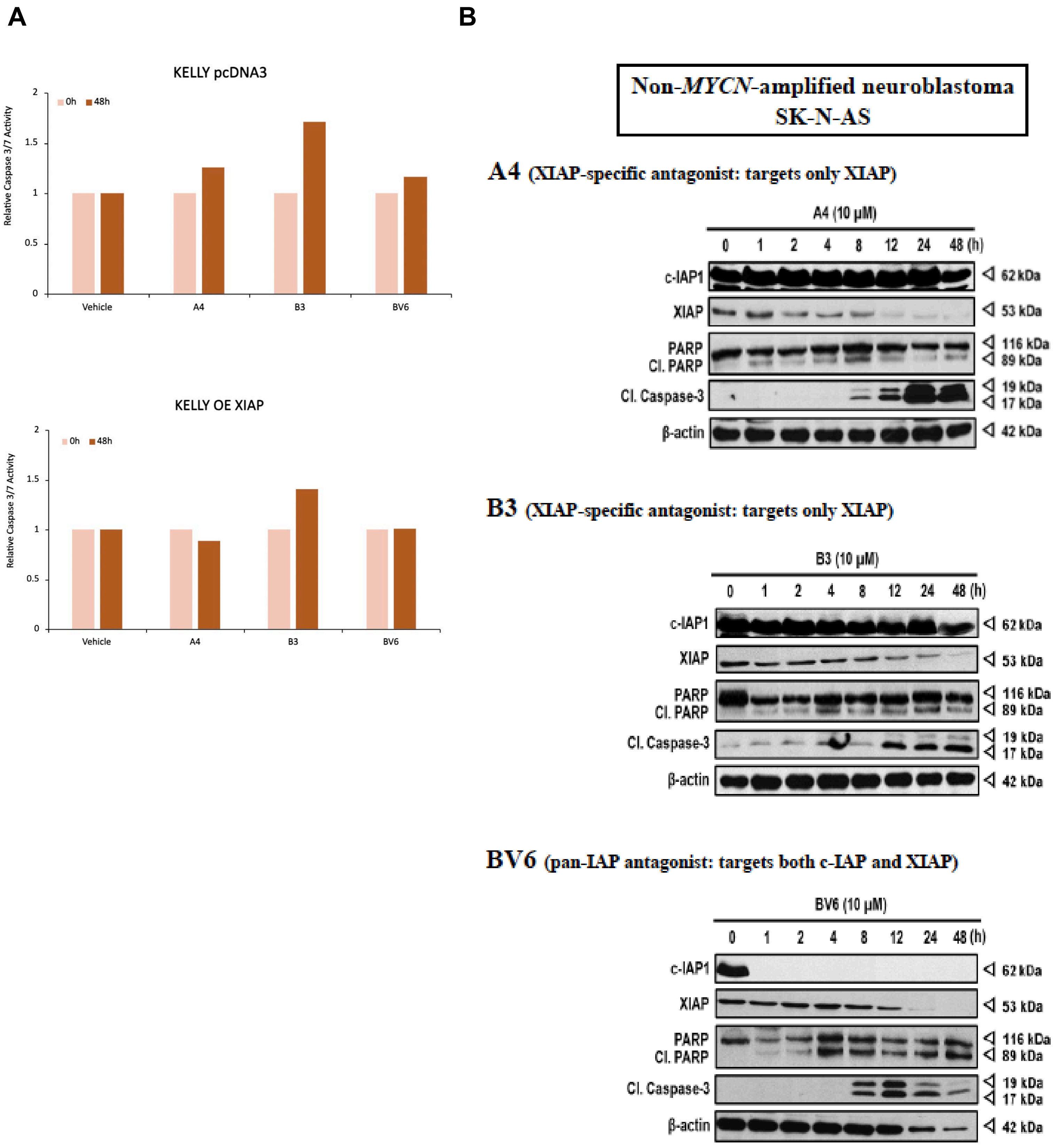

Figure S1, related to Figure 2
