# Supplementary material for: Targeted Degradation of XIAP is Sufficient and Specific to Induce Apoptosis in MYCN-overexpressing High-risk Neuroblastoma
Source: Cancer Res Commun. 2023 Nov 22;3(11):2386–99. doi: 10.1158/2767-9764.CRC-23-0082 (PMC10681007; doi:10.1158/2767-9764.CRC-23-0082)
Supplement: Figure S2 — Supplementary Figure S2, related to Figure 3. NMR analysis on 1H-15N-HSQC spectra of XIAP in the absence and presence of A4. [file crc-23-0082-s09.pdf]

A

1H-15N-HSQC spectrum of XIAP

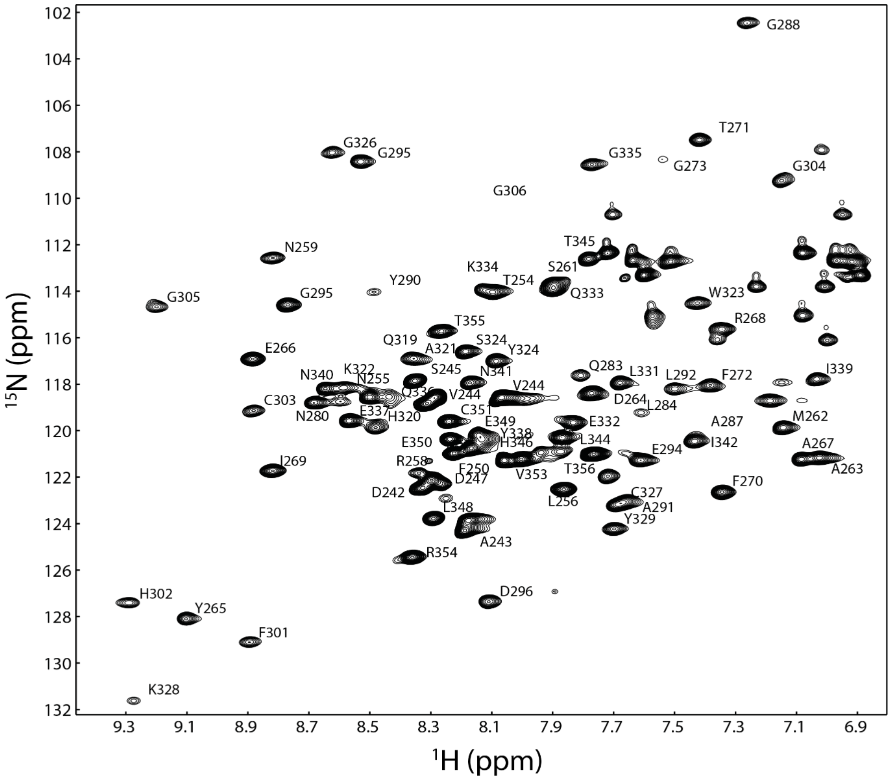

B

1H-15N-HSQC spectra of XIAP in the absence (black) or presence (red) of A4 compound

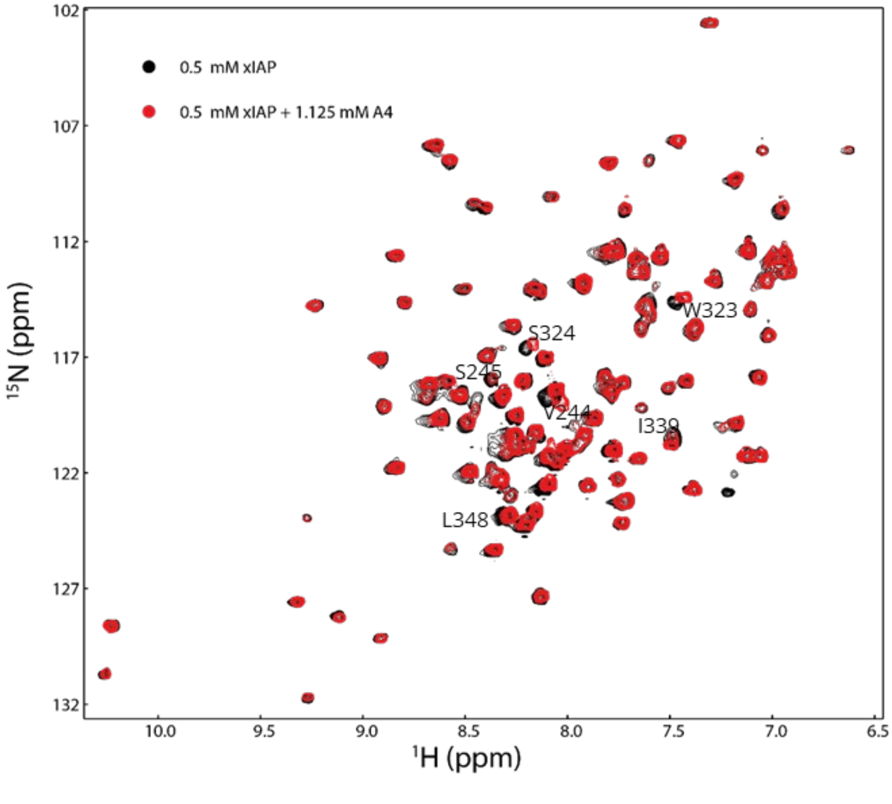

Figure S2, related to Figure 3
