# Supplementary figures and images for: Targeted Degradation of XIAP is Sufficient and Specific to Induce Apoptosis in MYCN-overexpressing High-risk Neuroblastoma
Source: Cancer Res Commun. 2023 Nov 22;3(11):2386–99. doi: 10.1158/2767-9764.CRC-23-0082 (PMC10681007; doi:10.1158/2767-9764.CRC-23-0082)

**A**

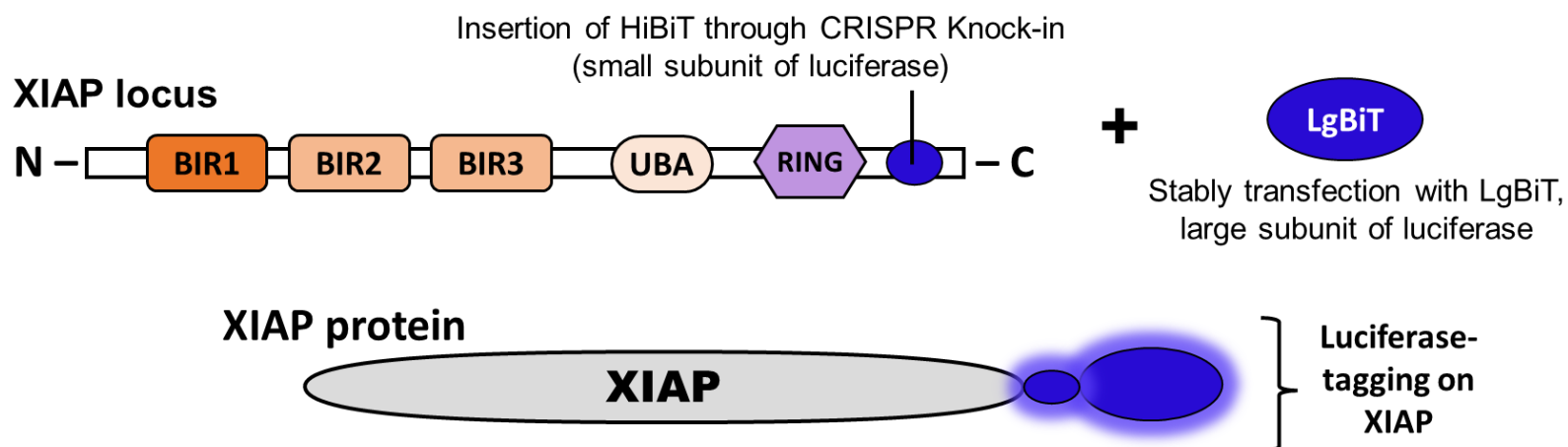

**B**

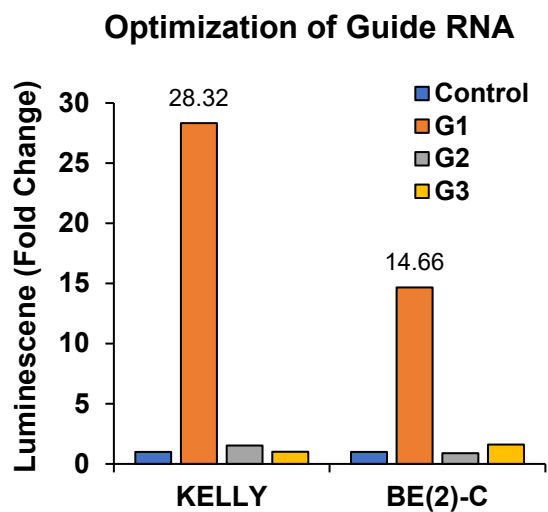

**C**

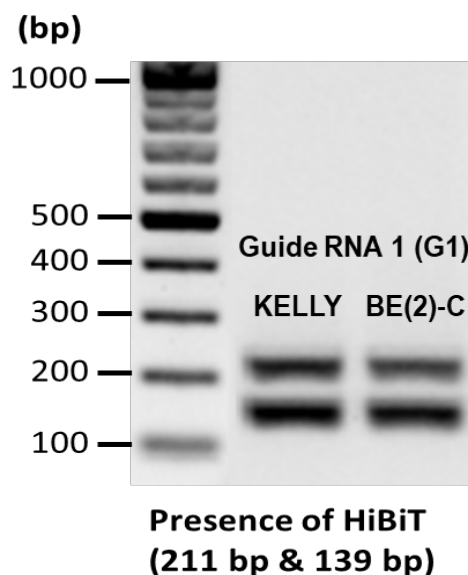

**D**

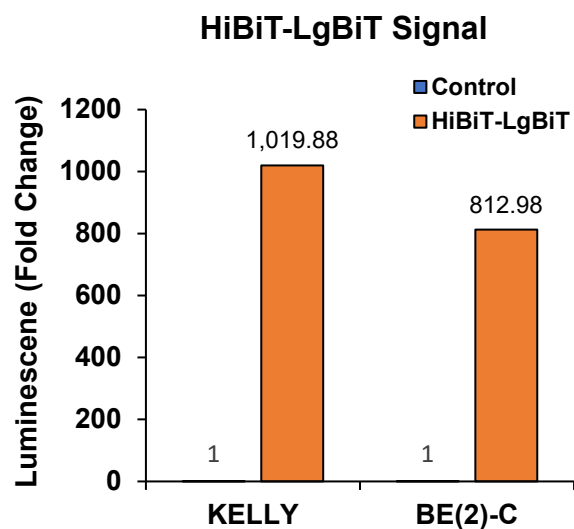

**Figure S3, related to Figure 3**

Supplement: Figure S3 — Supplementary Figure S3, related to Figure 3. Generation of luciferase-tagged XIAP using CRISPR knock-in gene editing. [file crc-23-0082-s10.pdf]

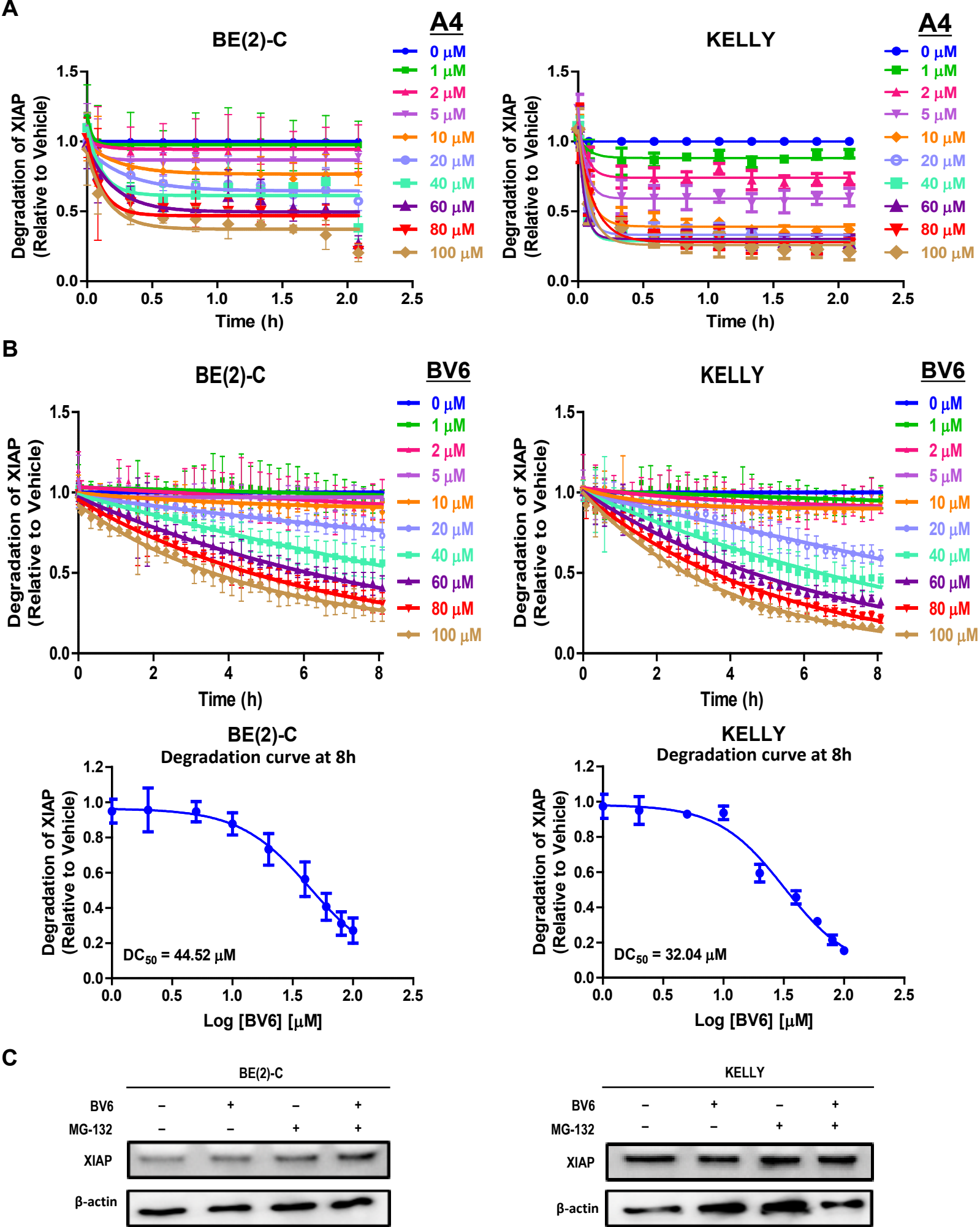

Figure S4, related to Figure 3

Supplement: Figure S4 — Supplementary Figure S4, related to Figure 3. Time course of XIAP degradation in response to XIAP-specific (A4) and pan-IAP (BV6) antagonists. [file crc-23-0082-s11.pdf]

A

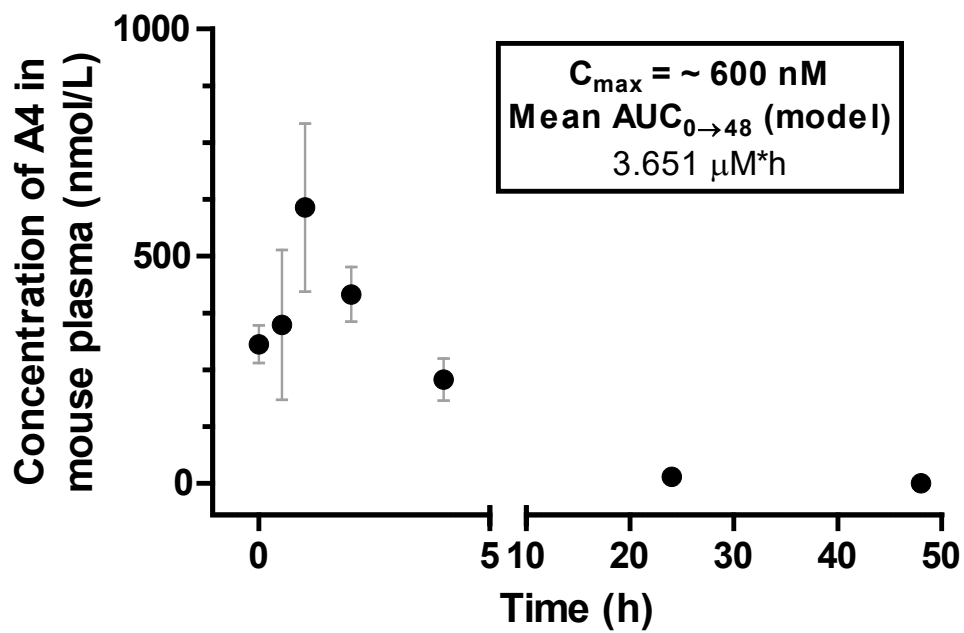

B

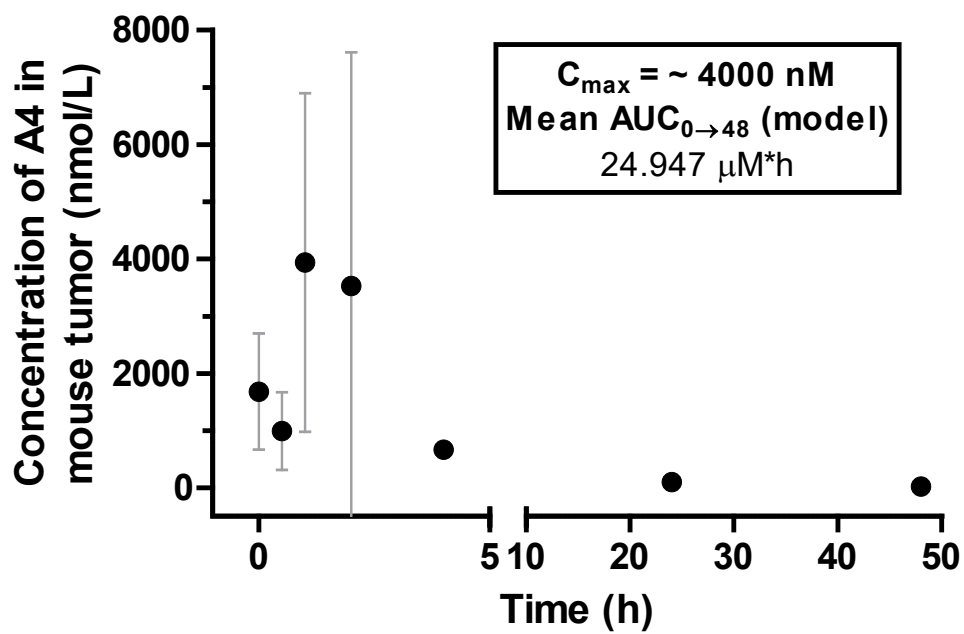

Figure S5, related to Figure 4

Supplement: Figure S5 — Supplementary Figure S5, related to Figure 4. Pharmacokinetic profile of XIAP-specific antagonist A4. [file crc-23-0082-s12.pdf]

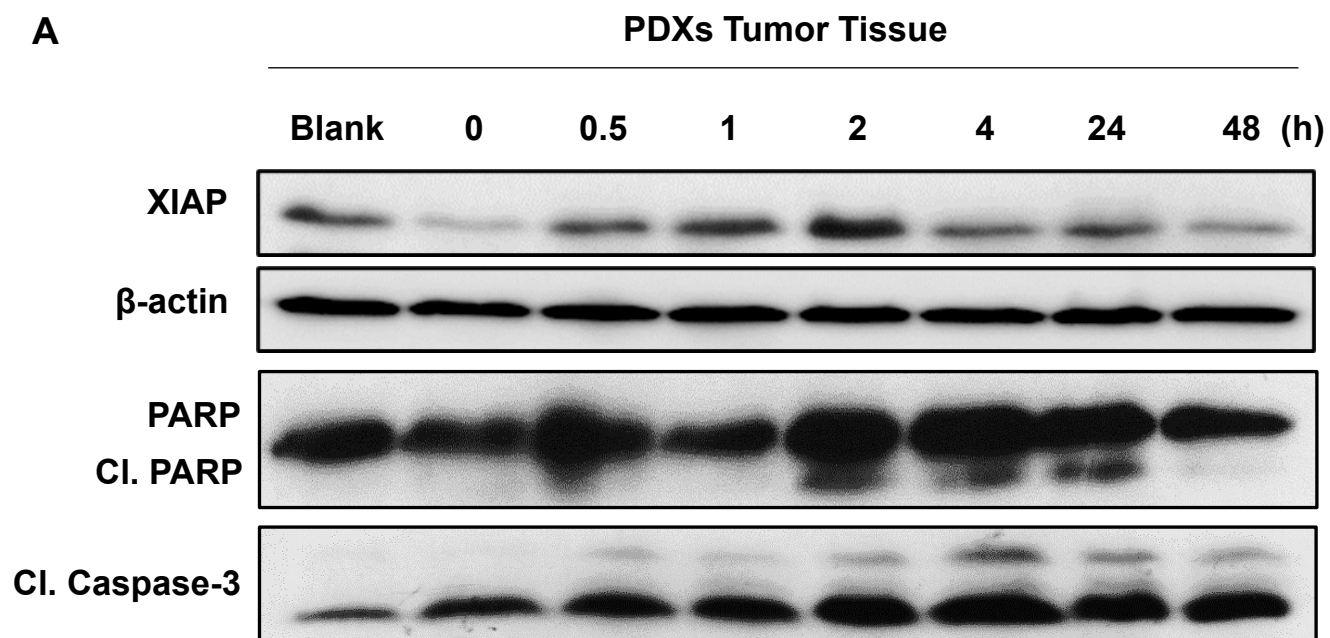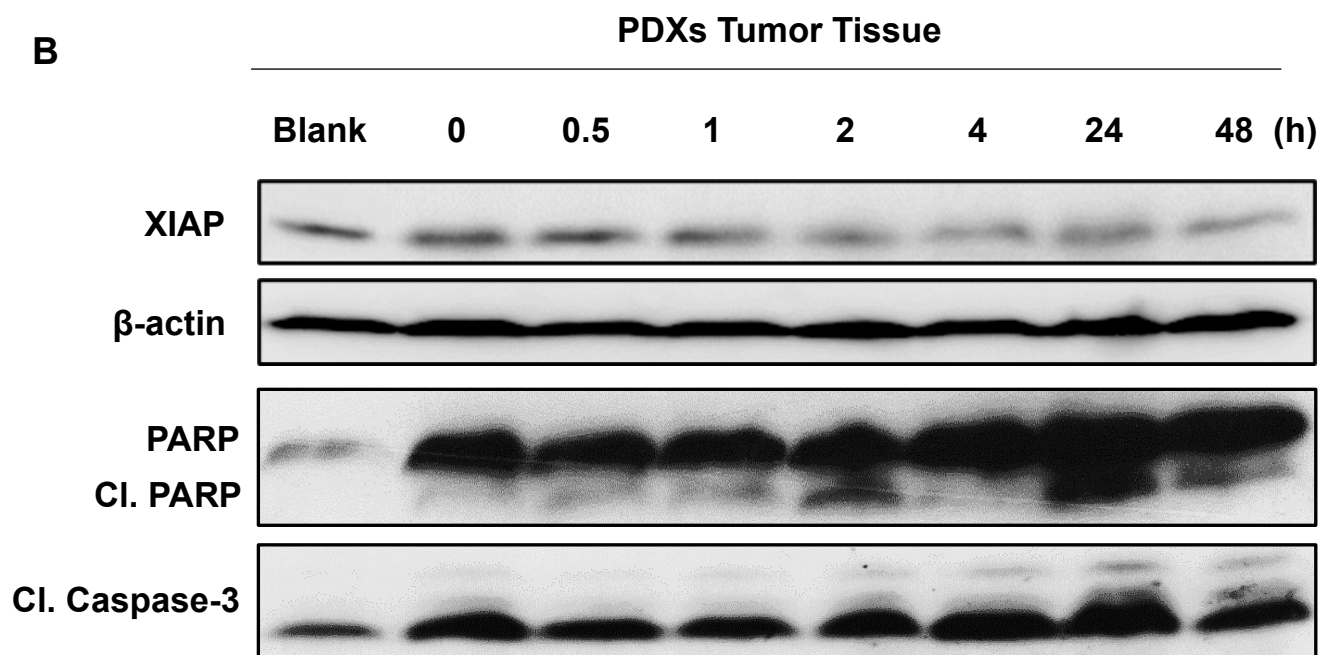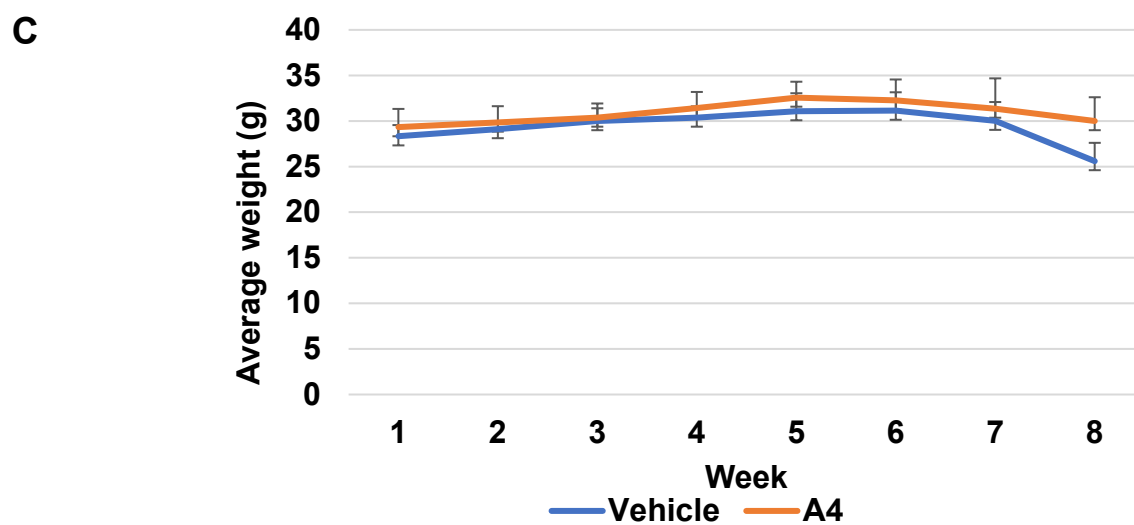

**Figure S6, related to Figure 4**

Supplement: Figure S6 — Supplementary Figure S6, related to Figure 4. Immunoblot analysis of tumor tissue from other sets of PDXs treated with A4. [file crc-23-0082-s13.pdf]
