# Supplementary material for: Targeted Degradation of XIAP is Sufficient and Specific to Induce Apoptosis in MYCN-overexpressing High-risk Neuroblastoma
Source: Cancer Res Commun. 2023 Nov 22;3(11):2386–99. doi: 10.1158/2767-9764.CRC-23-0082 (PMC10681007; doi:10.1158/2767-9764.CRC-23-0082)
Supplement: Figure S7 — Supplementary Figure S7, related to Figure 5. Vincristine or topotecan works synergistically with and promotes effective dose reduction of XIAP-specific antagonist A4 in vitro. [file crc-23-0082-s14.pdf]

A

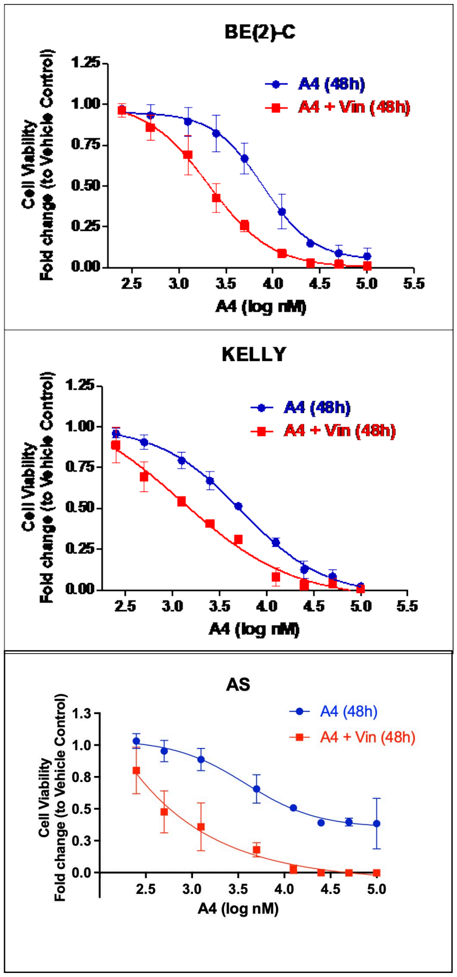

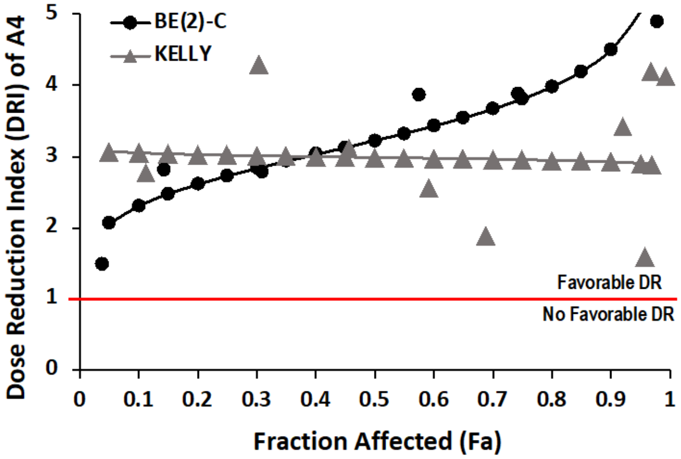

Cell lines	Dose Reduction Index (DRI) of A4	
	DRI_{60-90}	±SD
BE(2)-C	3.871	0.378
KELLY	2.948	0.018

B

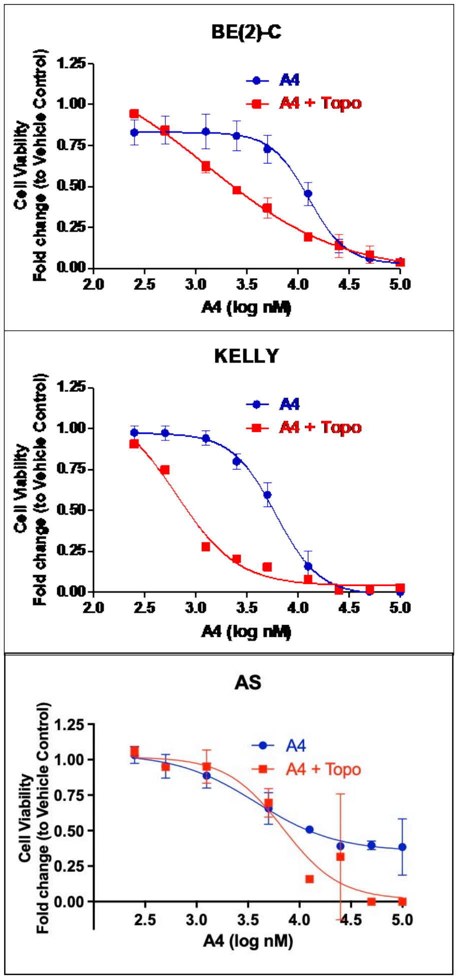

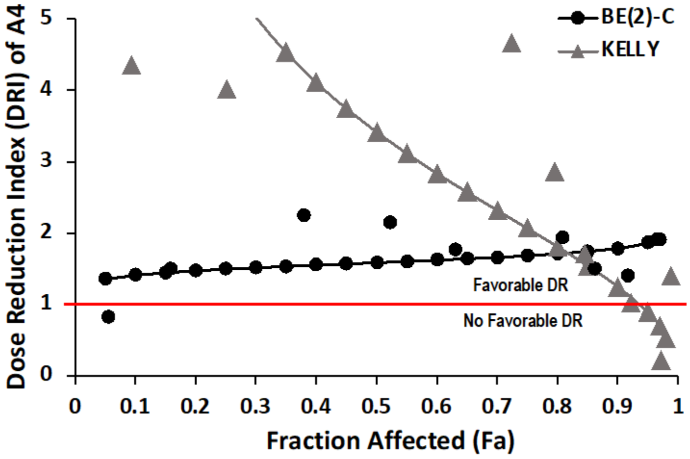

Cell lines	Dose Reduction Index (DRI) of A4	
	DRI_{60-90}	±SD
BE(2)-C	1.696	0.058
KELLY	2.598	0.565

Figure S7, related to Figure 5
